# Supplementary material for: Discovery of Tryptanthrin and Its Derivatives and Its Activities against NSCLC In Vitro via Both Apoptosis and Autophagy Pathways
Source: Int J Mol Sci. 2023 Jan 11;24(2):1450. doi: 10.3390/ijms24021450 (PMC9861433; doi:10.3390/ijms24021450)
Supplement: Supplementary file 1 [file ijms-24-01450-s001.zip › ijms-2066139-supplementary.pdf]

Discovery of tryptanthrin and its derivatives against NSCLC in vitro via both apoptosis and autophagy pathways

Ya-Yu Zou [1,†], Guang-Long Zhang [2,†], Cheng-Peng Li [1], Hai-Tao Long [1], Dan-Ping Chen [1], Zhu-Rui Li,[1] Gui-Ping Ouyang[1,2,3], Wen-Jing Zhang [1], Yi Zhang [1], and Zhen-Chao Wang [1,2,3*]

1 College of Pharmacy, Guizhou University, Guiyang 550025, China;
2 State Key Laboratory Breeding Base of Green Pesticide and Agricultural Bioengineering, Key Laboratory of Green Pesticide and Agricultural Bioengineering, Ministry of Education, Guiyang, 550025, China;
3 Guizhou Engineering Laboratory for Synthetic Drugs, Guizhou University, Guiyang, 550025, China.
* Correspondence: wzc.4884@163.com (Z.C.W.); Tel./Fax: +86-851-8830-8717 (Z.C.W.)
† These authors contributed equally to this work.

Content

1. General preparation for preparing intermediates 3a-i and 4a-i. 3

2. Synthesis of the target compounds A1–9 and B1–9 3

3. Synthesis of 4-azatryptanthrin derivatives C1–5 3

4. Synthesis of 4-azatryptanthrin derivatives D1–5...4

5. The data of title compounds. 4

6. ^{1}H NMR, ^{13}C NMR, ^{19}F NMR and HRMS spectra for target compounds. 12

7. Antiproliferative activity of compound C1 against normal cell lines. 58

8. Effects of C1 on the normal cell line HEK-293 58

9. Effects of C1 on expressions of EGFR, p-EGFR, Akt, p-Akt... 58

10. ADME properties 59

11. Reference... 60

1. General preparation for preparing intermediates 3a-i and 4a-i.

The syntheses of intermediates **3a-i** and **4a-i** referred to the literature [1, 2]. Isatins derivatives [3] ware synthesized by the most common procedure for the Sandmeyer process which utilizes a mixture of chloral hydrate, corresponding aromatic amine compounds, hydroxylamine, hydrochloric Acid, sulfate-saturated aqueous and concentrated sulfuric acid heating system. Substituted indigo anhydride was obtained by oxidation of m-chloroperoxybenzoic acid added in batches under ice bath conditions.

2. Synthesis of the target compounds A1−9 and B1−9

As outlined in Scheme 1, title compounds **A1−9** were prepared by condensation of the unsubstituted isatin with the substituted isatoic anhydride, using acetonitrile as solvent, triethylamine as catalyst, then subjected to reflux at 120°C for 3-5 h, after cooling, the resulting precipitates were filtrated, washed with MeOH (3×10 mL) to provide the A series compounds. Then, the target compounds of **B1−9** were obtained by condensation of the unsubstituted isatoic anhydride with the substituted isatin. The crude product was further purified to obtain the final target compounds **A1−9** and **B1−9**.

3. Synthesis of 4-azatryptanthrin derivatives C1−5

To a N,N-Dimethylformamide (DMF)solution of 2-aminonicotinic acid (0.69 g, 5.0 mmol), the corresponding O-Benzotriazole-N,N,N',N'-tetramethyl-uronium-hexafluorophosphate (HBTU, 1.90 g, 5.0 mmol), N-

methylmorpholine (NMM,1.01 g,10 mmol) were added. In another flask, to a solution of substituent isatin (5.0 mmol) in corresponding DMF was added 1,8-Diazabicyclo[5.4.0]undec-7-ene(DBU) (2.0 mL) stirring 30 min at room temperature. After that, add dropwise to another flask. The reaction mixture was stirred at room temperature overnight. Reaction completion was determined by TLC (DCM/MeOH (v/v) = 20:1). When it was completed, the reaction was quenched with water. The aqueous phase was extracted with chloroform three times (3×40 mL). The combined organic layer was washed successively with distilled water (50 mL) and dried over anhydrous sodium sulfate, filtered and solvent removed in vacuo and the residue was then purified on a silica gel column eluted to obtain compounds **C1–5.**

4. Synthesis of 4-azatryptanthrin derivatives D1–5

A solution of compound C3 (0.28 g, 1.0 mmol), potassium carbonate (0.41 g, 3.0 mmol) and corresponding aliphatic amines in N-methyl-2-pyrrolidone solution (5 mL) was heated at 90^{0}C for 3 h. When the reaction was completed, the mixture was filtered, and the filter cake was washed with water (10 mL) and ethanol (10 mL), successively. Then, the residue was purified by chromatography on a column of silica gel (with 10:1 dichloromethane / methanol) as the eluent to afford **D1-5** as a red solid.

5. The data of title compounds.

1-fluoroindolo[2,1-b]quinazoline-6,12-dione (A1). Yellow solid; yield=79%;

M.P. >300°C; ^{1}H NMR (400 MHz, DMSO-d_6) δ 8.48 (d, J = 7.9 Hz, 1H), 8.00 – 7.84 (m, 3H), 7.79 (d, J = 8.0 Hz, 1H), 7.59 – 7.41 (m, 2H); ^{13}C NMR (101 MHz, DMSO-d_6) δ 181.19, 149.63, 146.27, 133.20, 129.21, 123.60, 120.11, 118.87, 118.21, 116.18; ^{19}F NMR (376 MHz, DMSO-d$_6$) δ -110.23 (dd, J = 11.3, 5.4 Hz); HRMS calcd for $[M+H]^+$ $C_{15}H_7O_2N_2F$: 267.0564 found 267.0561.

2-fluoroindolo[2,1-b]quinazoline-6,12-dione (A2). Yellow solid; yield=82%; M.P. >300°C; ^{1}H NMR (400 MHz, DMSO-d_6) δ 8.49 (d, J = 8.0 Hz, 1H), 8.05 (dt, J = 8.2, 4.0 Hz, 2H), 7.94 – 7.78 (m, 3H), 7.51 (t, J = 7.5 Hz, 1H); ^{13}C NMR (101 MHz, DMSO-d_6) δ 146.11, 138.35, 133.20, 133.11, 127.48, 125.52, 123.54, 123.30, 122.02, 118.04, 113.42, 113.18. ^{19}F NMR (376 MHz, DMSO-d_6) δ -108.98 (dt, J = 8.4, 4.2 Hz); HRMS calcd for $[M+H]^+$ $C_{15}H_7O_2N_2F$: 267.0564 found 267.0557.

2-chloroindolo[2,1-b]quinazoline-6,12-dione (A3). Yellow solid; yield=68%; M.P. 260-261°C; ^{1}H NMR (400 MHz, DMSO-d_6) δ 8.47 (s, 1H), 8.28 (s, 1H), 7.95 (d, J = 41.7 Hz, 4H), 7.51 (s, 1H); ^{13}C NMR (126 MHz, Chloroform-d) δ 182.60, 165.64, 157.36, 148.97, 145.67, 144.35, 139.20, 137.82, 130.13, 125.81, 121.88, 120.56, 118.53, 117.79, 116.28; HRMS calcd for $[M+H]^+$ $C_{15}H_7O_2N_2Cl$: 283.0269 found 283.0261.

2-bromoindolo[2,1-b]quinazoline-6,12-dione (A4). Yellow solid; yield=72%; M.P. >300°C; ^{1}H NMR (400 MHz, DMSO-d_6) δ 8.48 (s, 1H), 8.41 (s, 1H), 8.13 (s, 1H), 7.91 (s, 3H), 7.51 (s, 1H); ^{13}C NMR (101 MHz, DMSO-d_6) δ 180.70, 157.53, 152.83, 150.43, 149.21, 147.17, 146.50, 141.20, 133.26, 128.98, 123.59, 120.21, 119.89, 118.3; HRMS calcd for $[M+H]^+$ $C_{15}H_7O_2N_2Br$: 326.9769 found 326.9753.

2-methylindolo[2,1-b]quinazoline-6,12-dione (A5). Yellow solid; yield=85%; M.P. 256-258°C; ^{1}H NMR (400 MHz, Chloroform-d) δ 8.65 (d, J = 8.1 Hz, 1H), 8.25 (s, 1H), 7.93 (dd, J = 7.9, 4.6 Hz, 2H), 7.80 (t, J = 7.8 Hz, 1H), 7.67 (dd, J = 8.2, 2.1 Hz, 1H), 7.44 (t, J = 7.5 Hz, 1H), 2.58 (s, 3H); ^{13}C NMR (101 MHz, DMSO-d_6) δ 182.93, 158.12, 146.38, 144.91, 144.84, 140.75, 138.16, 136.74, 130.26, 127.31,

127.05, 125.14, 123.54, 122.78, 117.50; HRMS calcd for $[M+H]^+$ $C_{16}H_{10}O_2N_2$: 263.0815 found 267.0803.

2-methoxyindolo[2,1-b]quinazoline-6,12-dione (A6). Yellow solid, yield=81%. M.P. 261-263°C; ^{1}H NMR (400 MHz, DMSO-d_6) δ 8.49 (d, J = 8.0 Hz, 1H), 7.93 – 7.84 (m, 3H), 7.73 (d, J = 3.0 Hz, 1H), 7.57 – 7.45 (m, 2H), 3.97 (s, 3H); ^{13}C NMR (101 MHz, DMSO-d_6) δ 180.47, 156.65, 146.04, 142.96, 118.92, 118.73, 115.69, 115.49, 115.03, 114.83, 107.77, 107.53; HRMS calcd for $[M+H]^+$ $C_{16}H_{10}O_3N_2$: 279.0764 found 279.0758.

2-nitroindolo[2,1-b]quinazoline-6,12-dione (A7). Yellow solid; yield=76%; M.P.>300°C; ^{1}H NMR (400 MHz, DMSO-d_6) δ 8.97 (d, J = 2.7 Hz, 1H), 8.68 (dd, J = 8.8, 2.7 Hz, 1H), 8.50 (d, J = 8.0 Hz, 1H), 8.19 (d, J = 8.9 Hz, 1H), 7.93 (dd, J = 14.2, 7.5 Hz, 2H), 7.54 (t, J = 7.5 Hz, 1H); ^{13}C NMR (101 MHz, DMSO-d_6) δ 182.60, 165.64, 157.36, 145.67, 144.35, 139.20, 137.82, 130.13, 125.81, 121.88, 120.56, 118.53, 117.79, 116.28; HRMS calcd for $[M-H]^-$ $C_{15}H_7O_4N_3$: 292.0364 found 292.0367.

1-chloroindolo[2,1-b]quinazoline-6,12-dione (A8). Yellow solid; yield=72%; M.P. >300°C; ^{1}H NMR (400 MHz, DMSO-d_6) δ 8.49 (d, J = 8.0 Hz, 1H), 7.94 – 7.83 (m, 4H), 7.76 (dd, J = 7.5, 1.7 Hz, 1H), 7.50 (td, J = 7.5, 0.9 Hz, 1H); ^{13}C NMR (101 MHz, DMSO-d_6) δ 182.82, 156.40, 149.55, 146.45, 145.90, 138.31, 135.43, 134.33, 132.80, 130.04, 127.45, 125.22, 122.61, 120.69, 117.66; HRMS calcd for $[M+H]^+$ $C_{15}H_7O_2N_2Cl$: 283.0269 found 283.0260.

2,3-difluoroindolo[2,1-b]quinazoline-6,12-dione (A9). Yellow solid;

yield=70%; M.P. >300°C; ^{1}H NMR (400 MHz, DMSO-*d*$_6$) δ 8.47 (d, *J* = 7.9 Hz, 1H), 8.30 (dd, *J* = 10.3, 8.5 Hz, 1H), 8.15 (dd, *J* = 11.0, 7.3 Hz, 1H), 8.00 – 7.77 (m, 2H), 7.51 (dd, *J* = 7.9, 7.0 Hz, 1H); ^{13}C NMR (101 MHz, DMSO-*d*$_6$) δ 182.60, 167.69, 165.64, 157.36, 148.97, 144.35, 139.20, 137.82, 130.13, 125.81, 121.88, 118.71, 117.79, 116.28; ^{19}F NMR (376 MHz, DMSO-*d*$_6$) δ -127.22 (ddd, *J* = 22.8, 11.0, 8.4 Hz), -133.38 (ddd, *J* = 22.5, 10.4, 7.3 Hz); HRMS calcd for $[2M-H]^-$ $C_{15}H_6O_2N_2F_2$: 567.0711 found 567.0728.

10-fluoroindolo[2,1-b]quinazoline-6,12-dione (B1). Yellow solid; yield=78%; M.P. 263-264°C; ^{1}H NMR (400 MHz, DMSO-*d*$_6$) δ 8.27 (dd, *J* = 7.6, 1.2 Hz, 1H), 7.93 – 7.87 (m, 2H), 7.75 – 7.67 (m, 3H), 7.52 – 7.45 (m, 1H); ^{13}C NMR (101 MHz, Chloroform-*d*) δ 181.46, 157.93, 146.54, 144.52, 137.76, 135.34, 133.33, 130.92, 130.58, 127.63, 125.26, 123.64, 123.14, 119.23; ^{19}F NMR (376 MHz, DMSO-*d*$_6$) δ -105.04 (dd, J = 11.2, 3.6 Hz); HRMS calcd for $[M+H]^+$ $C_{15}H_7O_2N_2F$: 267.0564 found 267.0557.

8-fluoroindolo[2,1-b]quinazoline-6,12-dione (B2). Yellow solid; yield 92%; M.P. 274-275°C; ^{1}H NMR (400 MHz, DMSO-*d*$_6$) δ 8.45 (dd, *J* = 8.8, 4.2 Hz, 1H), 8.28 (dt, *J* = 7.9, 1.1 Hz, 1H), 7.93 – 7.89 (m, 2H), 7.76 (dd, *J* = 7.1, 2.7 Hz, 1H), 7.73 – 7.65 (m, 2H); ^{13}C NMR (101 MHz, DMSO-*d*$_6$) δ 182.18, 161.77, 159.82, 158.12, 146.90, 145.84, 142.86, 135.82, 130.50, 127.48, 124.56, 124.37, 123.74, 119.38, 112.04; ^{19}F NMR (376 MHz, DMSO-*d*$_6$) δ -114.52. HRMS calcd for $[M-H]^-$ $C_{15}H_7O_2N_2F$: 265.0408 found 265.0411.

8-chloroindolo[2,1-b]quinazoline-6,12-dione (B3). Yellow solid; yield=93%; M.P. 291-292°C; ^{1}H NMR (400 MHz, DMSO-*d*$_6$) δ 8.43 (d, *J* = 8.6 Hz, 1H), 8.32 – 8.23 (m, 1H), 7.94 – 7.91 (m, 3H), 7.88 (dd, *J* = 8.5, 2.2 Hz, 1H), 7.71 (dt, *J* = 8.2, 4.3 Hz, 1H); ^{13}C NMR (101 MHz, Chloroform-*d*) δ 181.46, 157.93, 146.54, 144.52,

137.76, 135.34, 133.33, 130.92, 130.58, 127.63, 125.26, 123.64, 123.14, 119.23; HRMS calcd for $[M+H]^+$ $C_{15}H_7O_2N_2Cl$: 283.0269 found 283.0258.

8-bromoindolo[2,1-b]quinazoline-6,12-dione (B4). Yellow solid; yield=87%; M.P. 281-282°C; ^{1}H NMR (400 MHz, DMSO-*d*$_6$) δ 8.42 (dd, *J* = 8.3, 0.7 Hz, 1H), 8.33 (d, *J* = 7.8 Hz, 1H), 8.11 – 8.01 (m, 2H), 7.99 – 7.92 (m, 2H), 7.76 (dt, *J* = 8.2, 4.2 Hz, 1H); ^{13}C NMR (101 MHz, Chloroform-*d*) δ 181.37, 157.95, 146.52, 144.94, 140.68, 138.51, 135.39, 130.93, 130.63, 128.25, 127.65, 123.61, 123.37, 120.74, 119.54; HRMS calcd for $[M+H]^+$ $C_{15}H_7O_2N_2Br$: 326.9769 found 326.9755.

8-methylindolo[2,1-b]quinazoline-6,12-dione (B5). Yellow solid; yield=84%; M.P.272-273°C; ^{1}H NMR (400 MHz, DMSO-*d*$_6$) δ 8.38 – 8.30 (m, 2H), 7.97 – 7.92 (m, 2H), 7.77 – 7.64 (m, 3H), 2.42 (s, 3H); ^{13}C NMR (101 MHz, DMSO-*d*$_6$) δ 183.01, 158.01, 146.96, 145.71, 138.60, 137.15, 135.54, 130.36, 130.28, 127.36, 125.25, 123.83, 122.79, 117.27, 20.90; HRMS calcd for $[M+H]^+$ $C_{16}H_{10}O_2N_2$: 263.0815 found 263.0802.

8-methoxyindolo[2,1-b]quinazoline-6,12-dione (B6). Yellow solid; yield=91%; M.P. 257-259°C; ^{1}H NMR (400 MHz, Chloroform-*d*) δ 8.53 (d, *J* = 8.8 Hz, 1H), 8.44 (dd, *J* = 8.0, 1.5 Hz, 1H), 8.03 (dd, *J* = 8.2, 1.2 Hz, 1H), 7.89 – 7.82 (m, 1H), 7.68 (td, *J* = 7.6, 1.2 Hz, 1H), 7.39 (d, *J* = 2.7 Hz, 1H), 7.32 (dd, *J* = 8.8, 2.8 Hz, 1H), 3.91 (s, 3H); ^{13}C NMR (101 MHz, Chloroform-*d*) δ 182.70, 158.75, 157.74, 146.63, 144.77, 140.47, 134.94, 130.72, 130.24, 127.43, 125.05, 123.92, 122.98, 119.17, 108.40, 56.01; HRMS calcd for $[M+H]^+$ $C_{16}H_{10}O_3N_2$: 279.0764 found 279.0761.

8-nitroindolo[2,1-b]quinazoline-6,12-dione (B7). Yellow solid; yield 83%; M.P. 290-292°C; ^{1}H NMR (400 MHz, DMSO-*d6*) δ 8.42 (dd, *J* = 8.3, 0.7 Hz, 1H), 8.33 (d, *J* = 7.8 Hz, 1H), 8.11 – 8.01 (m, 2H), 7.99 – 7.92 (m, 2H), 7.76 (dt, *J* = 8.2, 4.2 Hz, 1H); ^{13}C NMR (101 MHz, DMSO-*d6*) δ 145.28, 140.19, 135.84, 130.47, 127.53

(d, *J* = 10.3 Hz), 119.51 (d, *J* = 18.3 Hz); HRMS calcd for $[M-H]^-$ $C_{15}H_7O_4N_3$: 292.0364 found 292.0378.

7-chloroindolo[2,1-b]quinazoline-6,12-dione (B8). Yellow solid; yield=87%; M.P. >300°C; ^{1}H NMR (400 MHz, DMSO-d_6) δ 8.48 (dd, *J* = 8.1, 0.7 Hz, 1H), 8.33 (d, *J* = 7.8 Hz, 1H), 7.99 – 7.94 (m, 2H), 7.85 (t, *J* = 8.1 Hz, 1H), 7.75 (ddd, *J* = 8.2, 4.8, 3.7 Hz, 1H), 7.52 (dd, *J* = 8.2, 0.7 Hz, 1H); ^{13}C NMR (101 MHz, DMSO-d_6) δ 180.04, 158.16, 147.69, 146.84, 138.88, 135.85, 131.85, 130.41, 130.38, 128.45, 127.51, 123.54, 119.62, 116.17; HRMS calcd for $[M+H]^+$ $C_{15}H_7O_2N_2Cl$: 283.0269 found 283.0259.

8,9-difluoroindolo[2,1-b]quinazoline-6,12-dione (B9). Yellow solid; yield=75%; M.P. 278-279°C; ^{1}H NMR (400 MHz, DMSO-d_6) δ 8.39 (dd, *J* = 10.4, 6.5 Hz, 1H), 8.28 (d, *J* = 7.9 Hz, 1H), 8.10 (t, *J* = 8.3 Hz, 1H), 7.95 – 7.91 (m, 2H), 7.72 (ddd, *J* = 8.3, 5.4, 2.9 Hz, 1H); ^{13}C NMR (101 MHz, DMSO-d_6) δ 180.89, 157.99, 146.77, 145.31, 135.99, 130.65, 130.52, 127.48, 123.41, 114.84, 114.64, 107.67, 107.43; ^{19}F NMR (376 MHz, DMSO-d_6) δ -121.21 – -121.27 (m), -121.29 – -121.34 (m), -138.95 (ddd, *J* = 20.7, 8.9, 6.7 Hz); HRMS calcd for $[M+H]^+$ $C_{15}H_6O_2N_2F_2$: 285.0470 found 285.0461.

pyrido[2',3':4,5]pyrimido[1,2-a]indole-5,11-dione (C1). Yellow solid; yield=65%; M.P. >300°C; ^{1}H NMR (400 MHz, DMSO-d_6) δ 9.04 (dd, *J* = 4.6, 2.2 Hz,1H), 8.67 (dd, *J* = 7.8, 2.0 Hz, 1H), 8.41 (d, *J* = 8.0 Hz,1H), 7.88 (d, *J* = 7.4 Hz,2H), 7.71 (dd, *J* = 7.8, 4.6 Hz,1H), 7.47 (t, *J* = 7.4Hz,1H); ^{13}C NMR (101 MHz, DMSO-d_6) δ 182.76, 158.58, 157.70, 156.58, 148.08, 146.21, 138.42, 136.79, 127.75,

125.41, 125.20, 122.63, 119.69, 117.40; HRMS calcd for $[M+H]^+$ $C_{14}H_7O_2N_3$: 250.0611 found 250.0610.

7-fluoropyrido[2',3':4,5]pyrimido[1,2-a]indole-5,11-dione (C2). Yellow solid; yield=34%; M.P. >300°C; ^{1}H NMR (400 MHz, DMSO-d_6) δ 9.03 (dd, J = 4.6, 1.8 Hz, 1H), 8.66 (dd, J = 7.8, 1.9 Hz, 1H), 7.75 (m, 2H), 7.71 (dd, J = 8.0, 4.6 Hz, 1H), 7.52 (ddt, J = 11.8, 7.5, 3.7 Hz, 1H); ^{13}C NMR (101 MHz, DMSO-d_6) δ 182.22, 157.41, 156.64, 150.90, 148.18, 137.24, 129.87, 129.81, 127.35, 127.12, 126.44, 125.34, 121.65, 119.74; ^{19}F NMR (376 MHz, DMSO-d_6) δ -105.35 (d, J = 3.5 Hz), -105.38 (d, J = 3.7 Hz); HRMS calcd for $[M+H]^+$ $C_{14}H_6O_2N_3F$: 268.0517 found 268.0514.

10-chloropyrido[2',3':4,5]pyrimido[1,2-a]indole-5,11-dione (C3). Yellow solid; yield=68%; M.P.>300°C; ^{1}H NMR (400 MHz, DMSO-d_6) δ 9.10 (dd, J = 4.6, 2.0 Hz, 1H), 8.73 (dd, J = 7.9, 2.0 Hz, 1H), 8.46 (dd, J = 8.1, 0.7 Hz, 1H), 7.88 (t, J = 8.1 Hz, 1H), 7.77 (dd, J = 7.9, 4.6 Hz, 1H), 7.56 (dd, J = 8.2, 0.8 Hz, 1H); ^{13}C NMR (101 MHz, DMSO-d_6) δ 179.86, 158.54, 157.59, 156.84, 147.65, 147.36, 139.10, 136.95, 132.06, 128.85, 125.31, 119.60, 119.44, 116.08; HRMS calcd for $[M+H]^+$ $C_{14}H_6O_2N_3Cl$: 284.0211 found 284.0219.

9-methylpyrido[2',3':4,5]pyrimido[1,2-a]indole-5,11-dione (C4). Yellow solid; yield=57%; M.P. >300°C; ^{1}H NMR (400 MHz, Chloroform-d) δ 9.11 (s, 1H), 8.78 (d, J = 7.9 Hz, 1H), 8.48 (d, J = 8.1 Hz, 1H), 7.75 (s, 1H), 7.69 – 7.50 (m, 2H), 2.50 (s, 3H); ^{13}C NMR (101 MHz, Chloroform-d) δ 181.80, 156.41, 151.52, 147.60, 147.14, 140.57, 139.00, 138.15, 136.60, 125.84, 124.64, 117.61, 21.14; HRMS calcd for $[M+H]^+$ $C_{15}H_9O_2N_3$: 264.0768 found 264.0766.

9-methoxypyrido[2',3':4,5]pyrimido[1,2-a]indole-5,11-dione (C5). Red solid; yield=52%; M.P. 289-290°C; ^{1}H NMR (400 MHz, Chloroform-d) δ 9.10 (dd, J = 4.6, 2.0 Hz, 1H), 8.76 (dd, J = 8.0, 2.0 Hz, 1H), 8.50 (d, J = 8.8 Hz, 1H), 7.62 (dd, J = 7.9, 4.6 Hz, 1H), 7.41 (d, J = 2.7 Hz, 1H), 7.33 (dd, J = 8.8, 2.8 Hz, 1H), 3.93 (s, 3H); ^{13}C NMR (101 MHz, Chloroform-d) δ 181.72, 159.13, 157.81, 157.47, 156.33,

147.26, 139.85, 136.53, 124.98, 124.67, 122.95, 119.83, 119.05, 108.78, 56.07; HRMS calcd for [M+H]$^+$ $C_{15}H_9O_3N_3$: 280.0717 found 280.0713.

10-morpholinopyrido[2',3':4,5]pyrimido[1,2-a]indole-5,11-dione (D1). Red solid; yield=53%; M.P.>300°C; ^{1}H NMR (400 MHz, DMSO-*d*$_6$) *δ* 9.31 – 8.94 (m, 1H), 8.77 (d, *J* = 7.8 Hz, 1H), 8.11 (d, *J* = 7.6 Hz, 1H), 7.72 – 7.45 (m, 2H), 6.86 (d, *J* = 8.6 Hz, 1H), 4.62 – 3.75 (m, 4H), 3.49 (t, *J* = 4.6 Hz, 4H); ^{13}C NMR (101 MHz, DMSO-*d*$_6$) *δ* 178.08, 151.86, 146.94, 139.12, 132.67, 132.58, 123.31, 123.07, 115.44, 113.11, 112.87, 110.48, 108.59, 66.78, 51.09. HRMS calcd for [M-H]$^-$ $C_{18}H_{15}O_3N_5$: 348.1097 found 348.1084.

10-(4-methylpiperazin-1-yl)pyrido[2',3':4,5]pyrimido[1,2-a]indole-5,11-dione (D2). Red solid; yield=59%; M.P. 272-273°C; ^{1}H NMR (400 MHz, Chloroform-*d*) *δ* 9.09 (dd, *J* = 4.6, 2.0 Hz, 1H), 8.76 (dd, *J* = 7.9, 2.0 Hz, 1H), 8.06 (d, *J* = 7.6 Hz, 1H), 7.68 – 7.47 (m, 2H), 6.87 (d, *J* = 8.7 Hz, 1H), 3.53 (d, *J* = 5.1 Hz, 4H), 2.84 – 2.57 (m, 4H), 1.61 (s, 3H); ^{13}C NMR (101 MHz, Chloroform-*d*) *δ* 179.04, 158.22, 147.98, 146.77, 145.75, 144.86, 135.09, 130.54, 129.94, 127.28, 123.41, 112.98, 112.74, 106.26, 54.72, 49.71, 49.64, 46.02; HRMS calcd for [2M-H]$^-$ $C_{19}H_{18}O_2N_6$: 723.2910 found 723.2945.

10-(piperazin-1-yl)pyrido[2',3':4,5]pyrimido[1,2-a]indole-5,11-dione (D3). Red solid; yield=65%; M.P. >300°C; ^{1}H NMR (400 MHz, DMSO-d6) δ 9.06 (dd, *J* = 4.6, 2.0 Hz, 1H), 8.70 (dd, *J* = 7.9, 2.0 Hz, 1H), 7.95 (d, *J* = 7.6 Hz, 1H), 7.79 – 7.61 (m, 2H), 7.03 (d, *J* = 8.6 Hz, 1H), 3.44 (d, *J* = 5.1 Hz, 4H), 3.17 – 3.03 (m, 4H); ^{13}C NMR (101 MHz, Chloroform-*d*) *δ* 177.81, 152.18, 146.81, 138.88, 132.60, 132.51, 123.25, 123.01, 115.76, 113.05, 112.81, 110.25, 108.02, 52.18, 46.04; HRMS calcd for [M-H]$^-$ $C_{18}H_{16}O_2N_6$: 347.1251 found 347.1230.

10-(4-(2-hydroxyethyl)piperazin-1-yl)pyrido[2',3':4,5]pyrimido[1,2-a]indole-5,11-dione (D4). Red solid; yield=55%; M.P.239-240°C; ^{1}H NMR (400 MHz, Chloroform-*d*) δ 9.10 (dd, *J* = 4.7, 2.0 Hz, 1H), 8.77 (dd, *J* = 7.8, 2.0 Hz, 1H), 8.08 (d, *J* = 7.6 Hz, 1H), 7.72 – 7.46 (m, 2H), 6.87 (d, *J* = 8.6 Hz, 1H), 3.72 (t, *J* = 5.4 Hz, 2H), 3.64 – 3.30 (m, 4H), 2.93 – 2.75 (m, 4H), 2.70 (t, *J* = 5.4 Hz, 2H); ^{13}C NMR (101 MHz, DMSO-d_6) δ 178.12, 158.57, 157.79, 156.42, 151.64, 146.92, 139.08, 136.75, 119.30, 116.58, 107.39, 60.64, 59.06, 53.56, 50.77; HRMS calcd for $[M+Na]^+$ $C_{20}H_{20}O_3N_6$: 415.1489 found 415.1472.

10-(pyrrolidin-1-yl)pyrido[2',3':4,5]pyrimido[1,2-a]indole-5,11-dione (D5). Purple red solid; yield=71%; M.P. 244-245°C; ^{1}H NMR (400 MHz, Chloroform-*d*) δ 9.07 (dd, *J* = 4.6, 2.0 Hz, 1H), 8.74 (dd, *J* = 7.9, 2.0 Hz, 1H), 7.88 (d, *J* = 7.4 Hz, 1H), 7.55 (dd, *J* = 7.9, 4.6 Hz, 1H), 7.43 (dd, *J* = 8.9, 7.5 Hz, 1H), 6.68 (d, *J* = 8.9 Hz, 1H), 3.74 – 3.66 (m, 4H), 2.12 – 2.05 (m, 4H); ^{13}C NMR (101 MHz, Chloroform-*d*) δ 176.72, 158.41, 157.76, 156.24, 152.29, 148.24, 146.24, 138.50, 136.60, 123.87, 119.06, 116.48, 109.54, 106.88, 52.40, 26.03, 24.06; HRMS calcd for $[M-H]^-$ $C_{18}H_{15}O_2N_5$: 332.1153 found 332.1142.

6. ^{1}H NMR, ^{13}C NMR, ^{19}F NMR and HRMS spectra for target compounds.

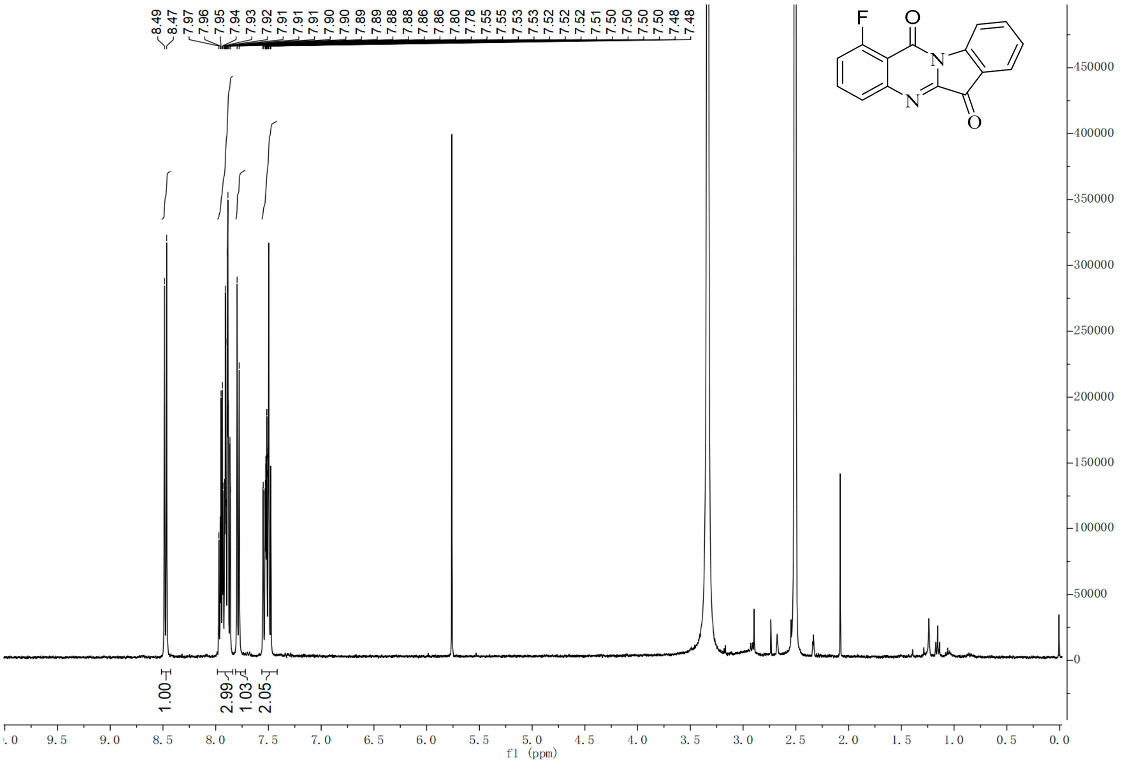

Figure S1. ^{1}H NMR Spectrum (DMSO-d_6, 400 MHz) of **A1**.

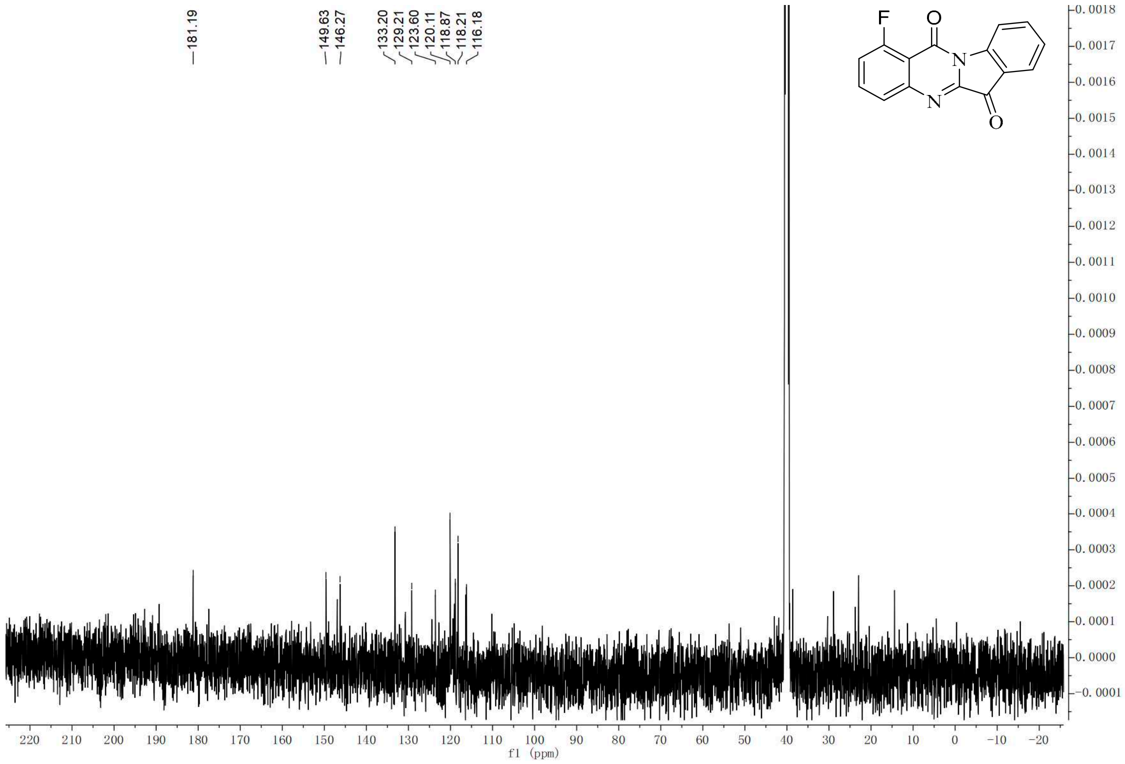

Figure S2. ^{13}C NMR Spectrum (DMSO-d_6, 101 MHz) of **A1**.

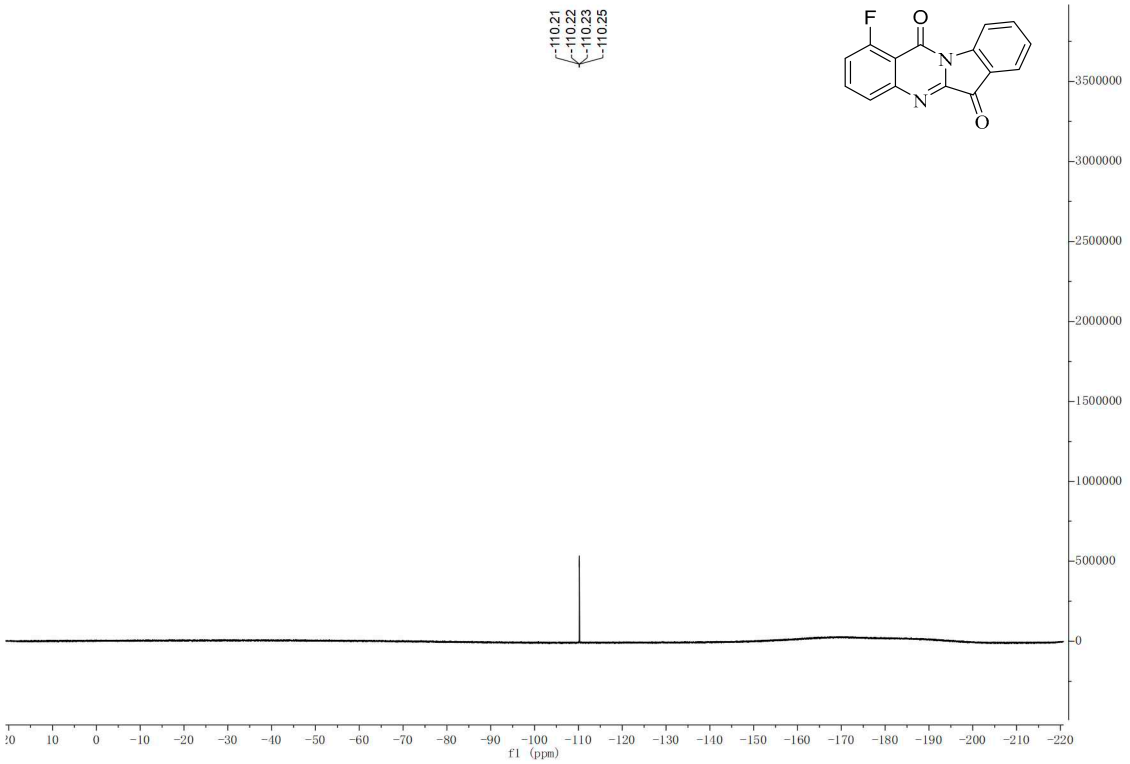

Figure S3. ^{19}F NMR Spectrum (DMSO-d_6, 376 MHz) of **A1**.

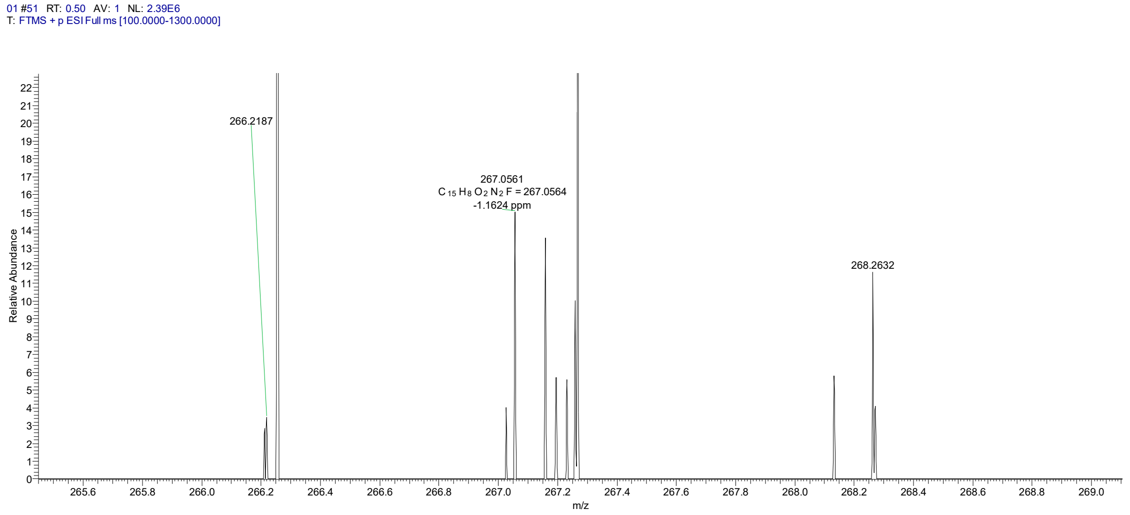

Figure S4. **A1** HRMS for $C_{15}H_7O_2N_2F$ $[M+H]^+$. Calcd. 267.0564. Found 267.0561.

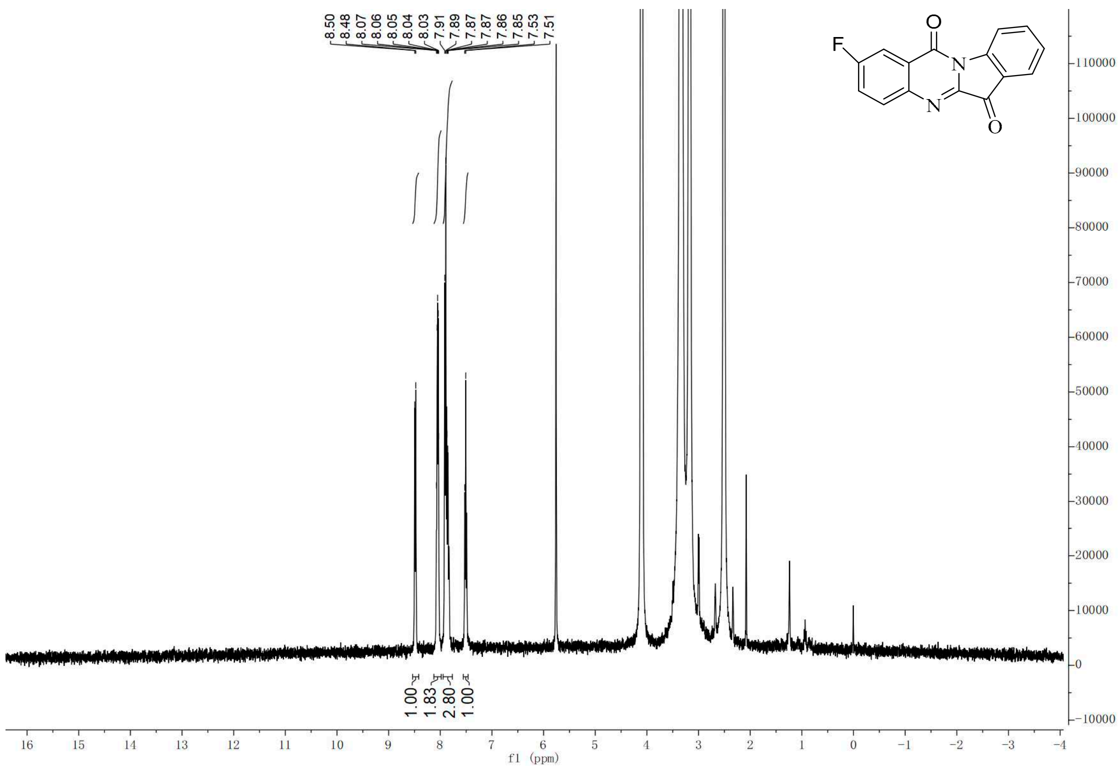

Figure S5. ^{1}H NMR Spectrum (DMSO-d_6, 400 MHz) of **A2**.

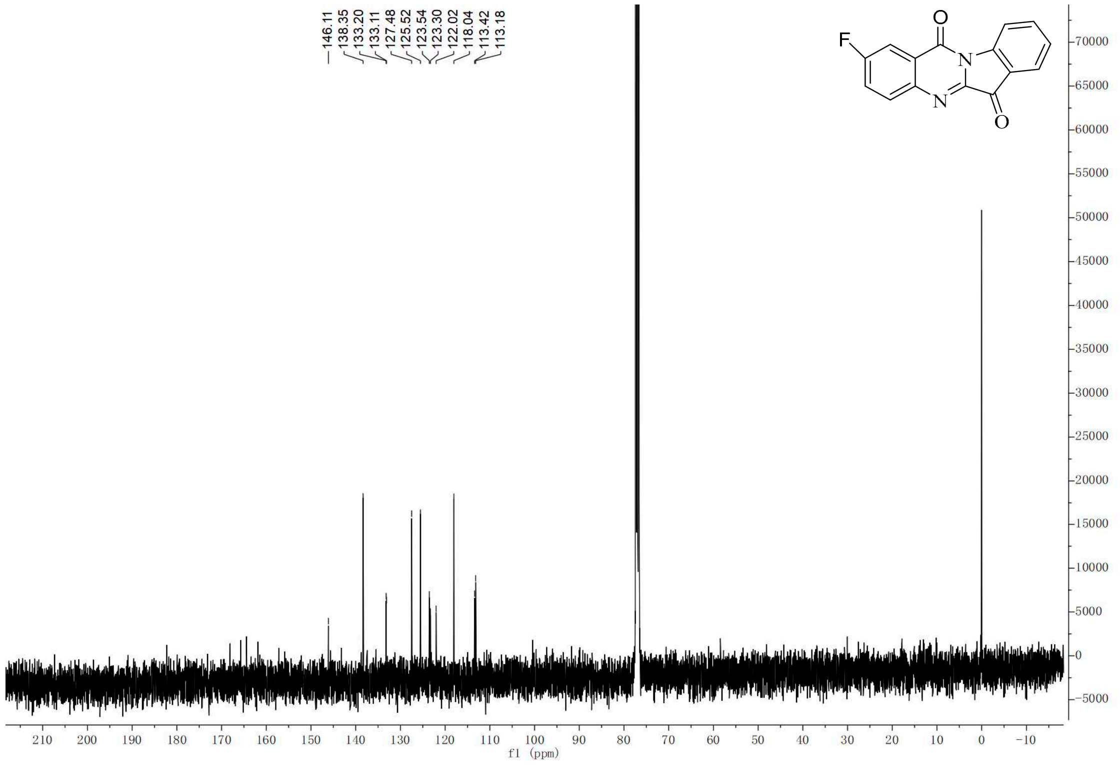

Figure S6. ^{13}C NMR Spectrum (DMSO-d_6, 101 MHz) of **A2**.

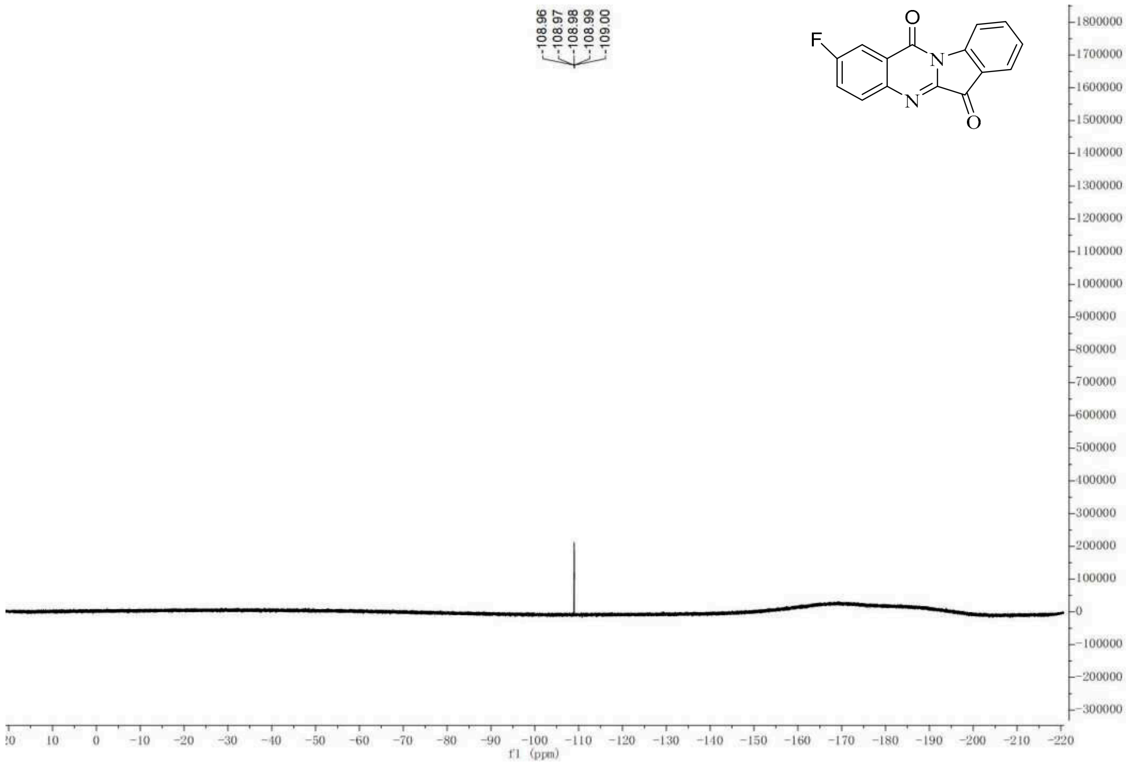

Figure S7. ^{19}F NMR Spectrum (DMSO-d_6, 376 MHz) of **A2**.

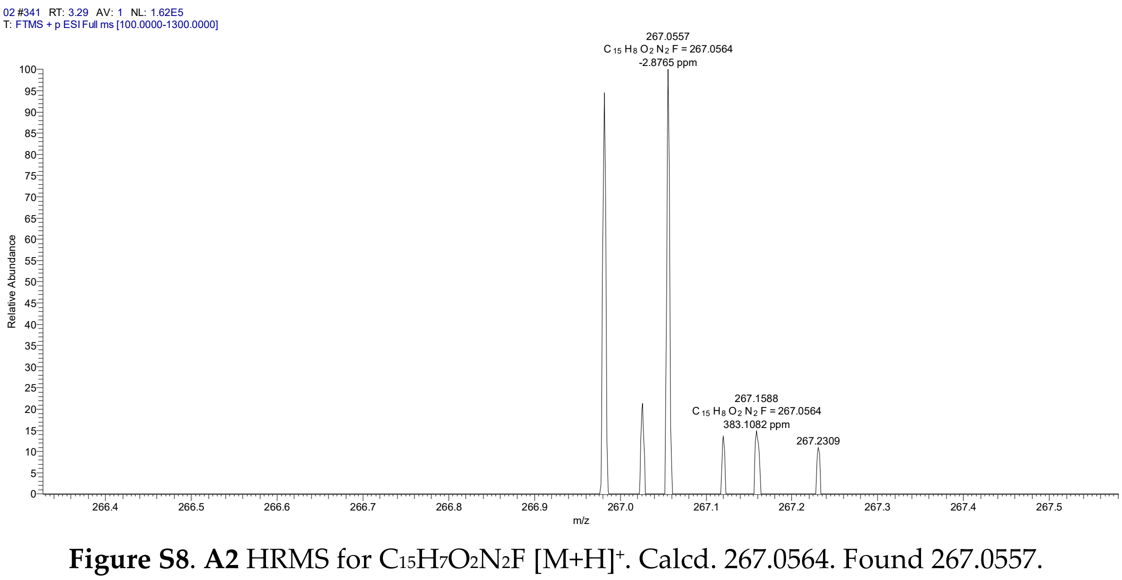

Figure S8. **A2** HRMS for $C_{15}H_7O_2N_2F$ $[M+H]^+$. Calcd. 267.0564. Found 267.0557.

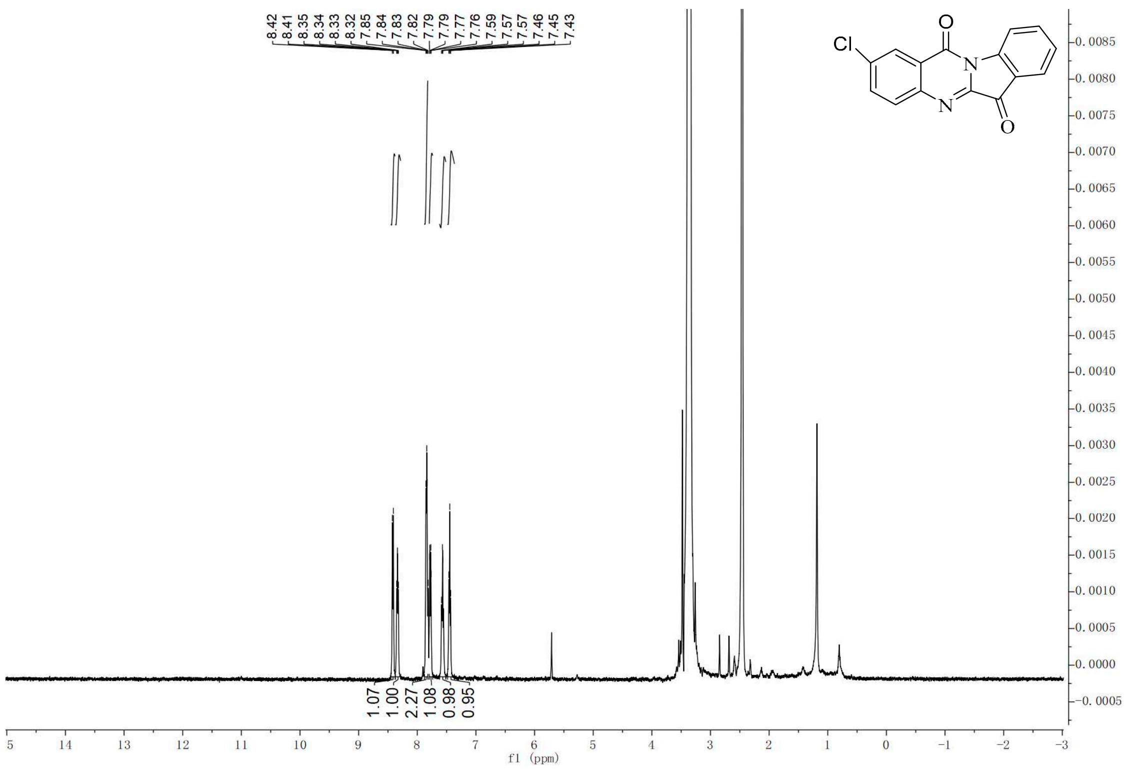

Figure S9. ^{1}H NMR Spectrum (DMSO-d_6, 400 MHz) of **A3**.

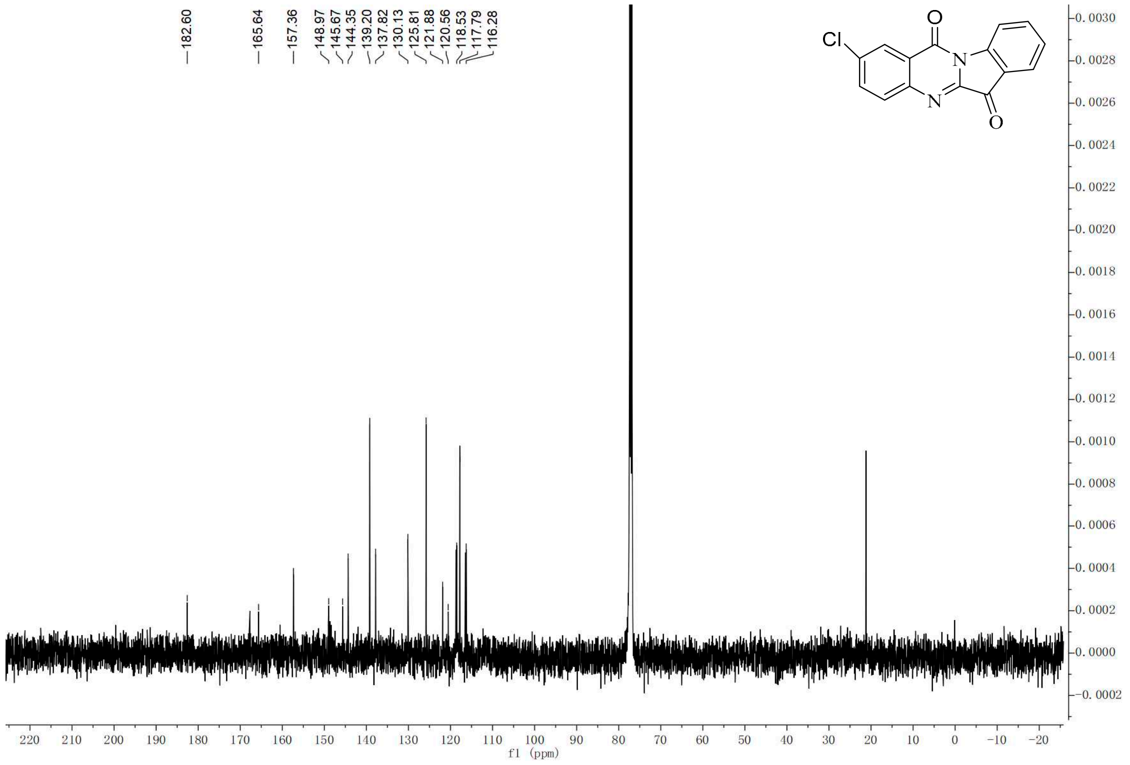

Figure S10. ^{13}C NMR Spectrum (CDCl3-d_6, 101 MHz) of **A3**.

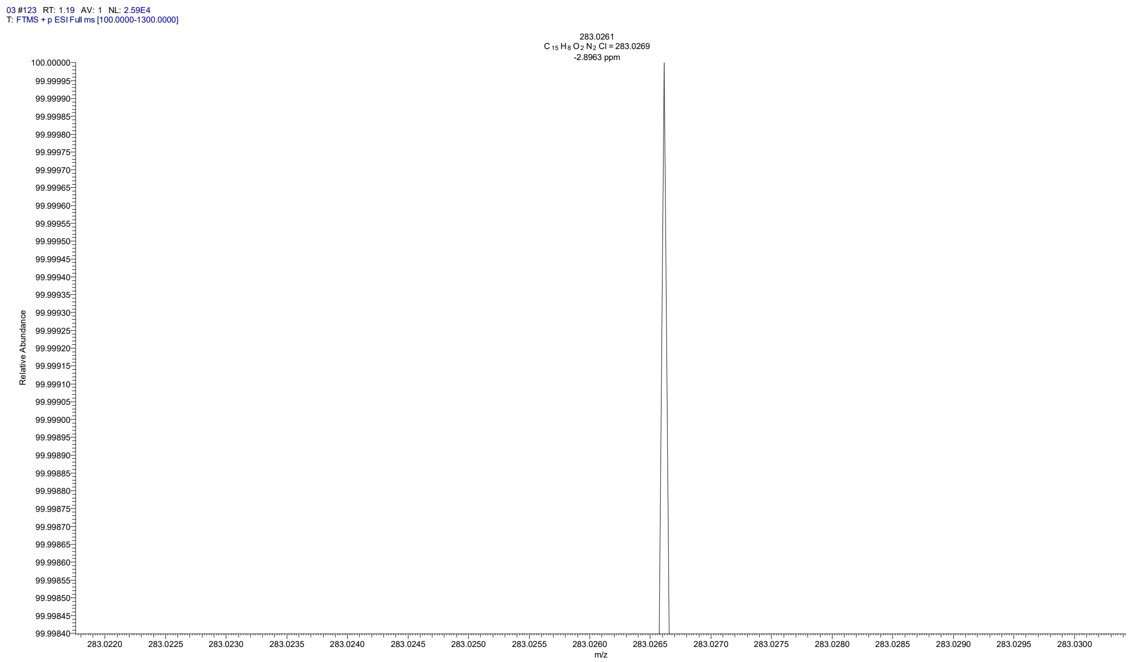

Figure S11. **A3** HRMS for $C_{15}H_7N_2O_2Cl$ $[M+H]^+$. Calcd. 283.0269. Found 283.0261.

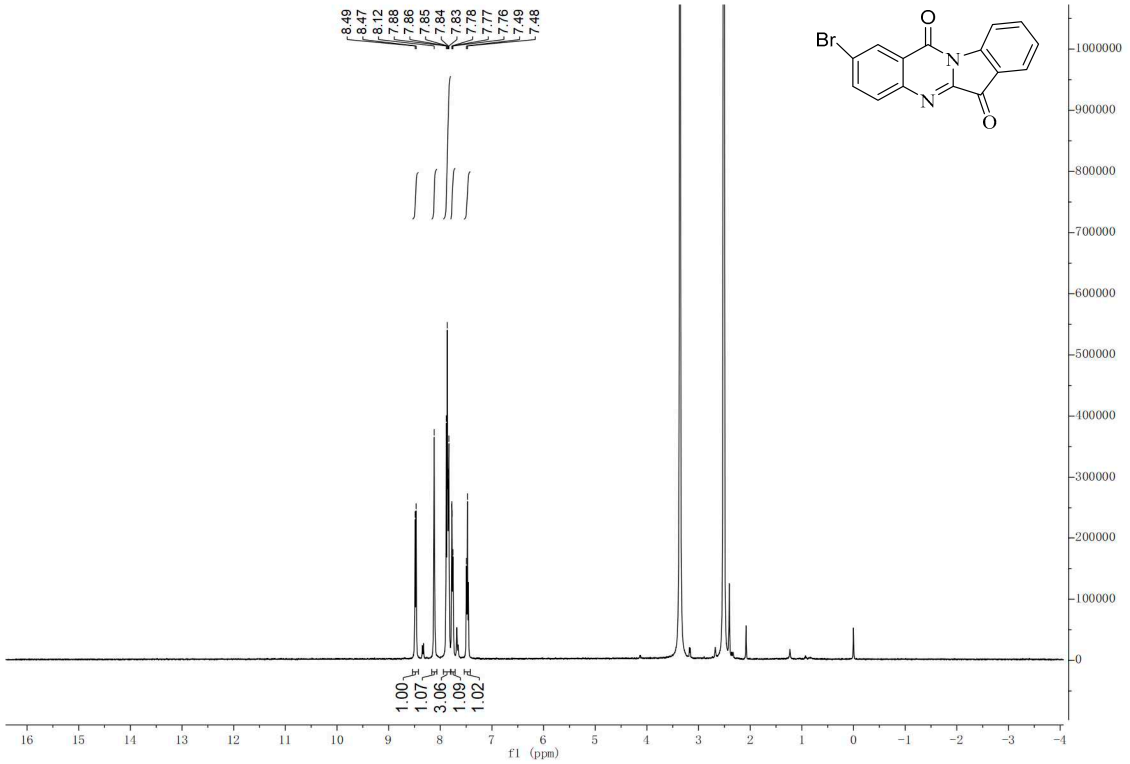

Figure S12. ^{1}H NMR Spectrum (DMSO-d_6, 400 MHz) of **A4**.

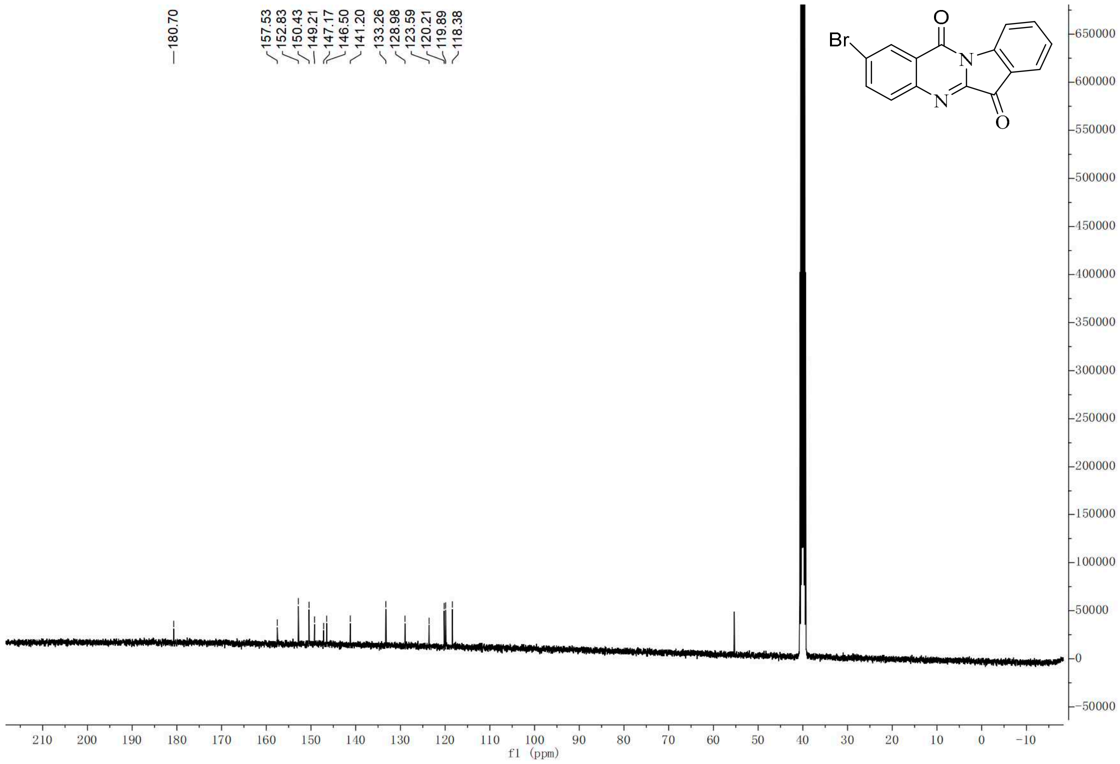

Figure S13. ^{13}C NMR Spectrum (DMSO-d_6, 101 MHz) of **A4**.

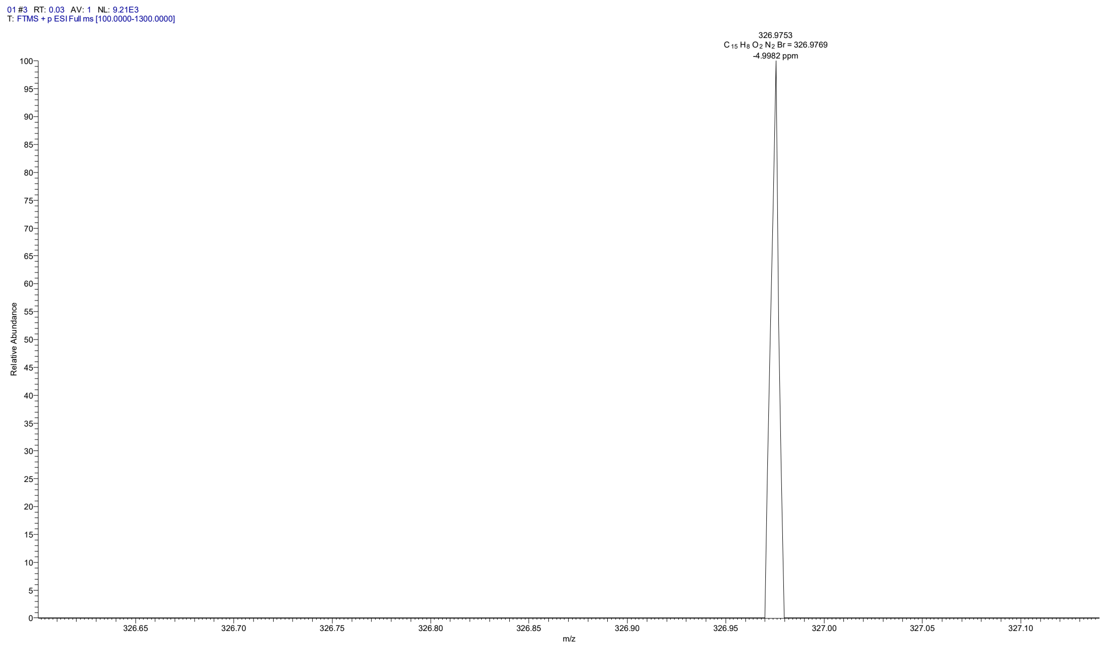

Figure S14. **A4** HRMS for $C_{15}H_7BrN_2O_2$ [M+H]$^+$. Calcd. 326.9769. Found 326.9753**.**

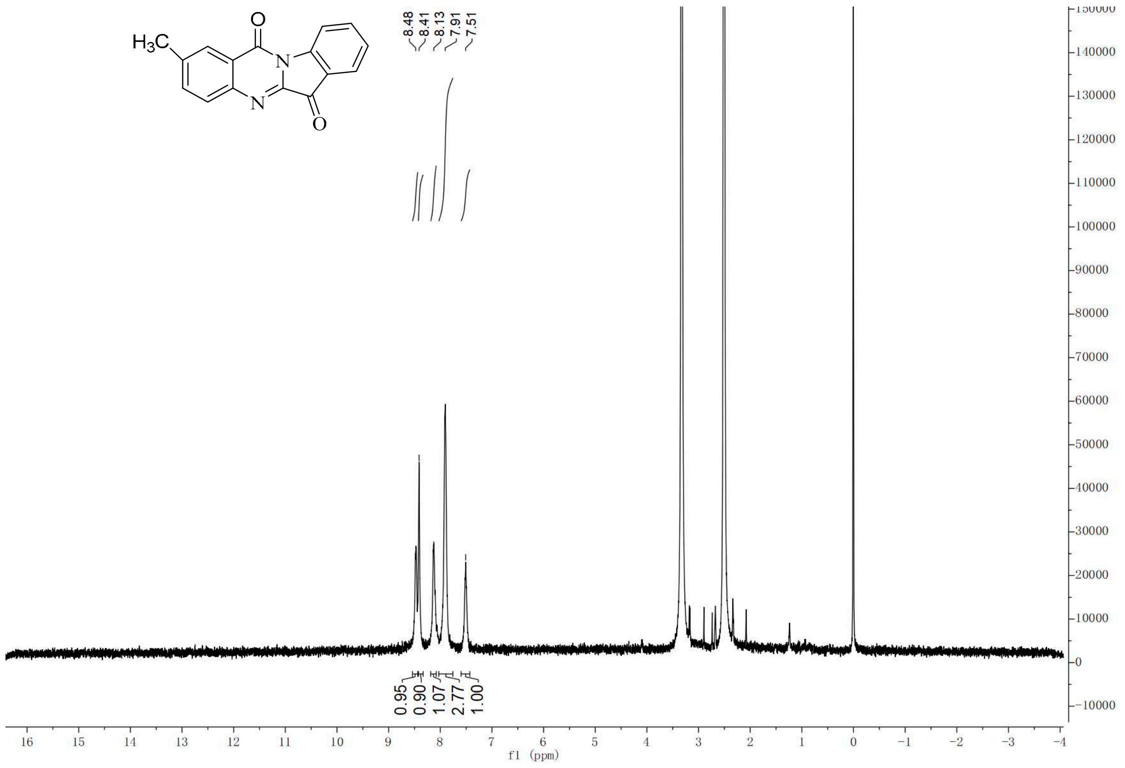

Figure S15. ^{1}H NMR Spectrum ($CDCl_3$-d_6, 400 MHz) of **A5**.

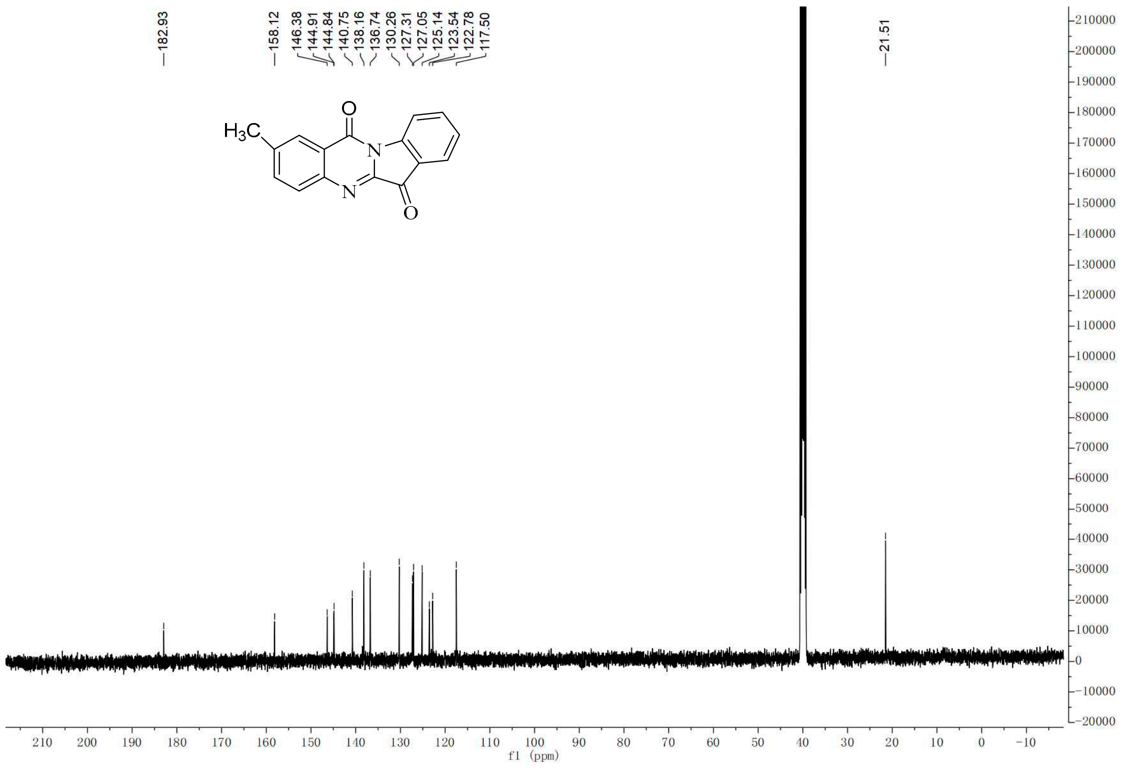

Figure S16. ^{13}C NMR Spectrum (DMSO-d_6, 101 MHz) of **A5**.

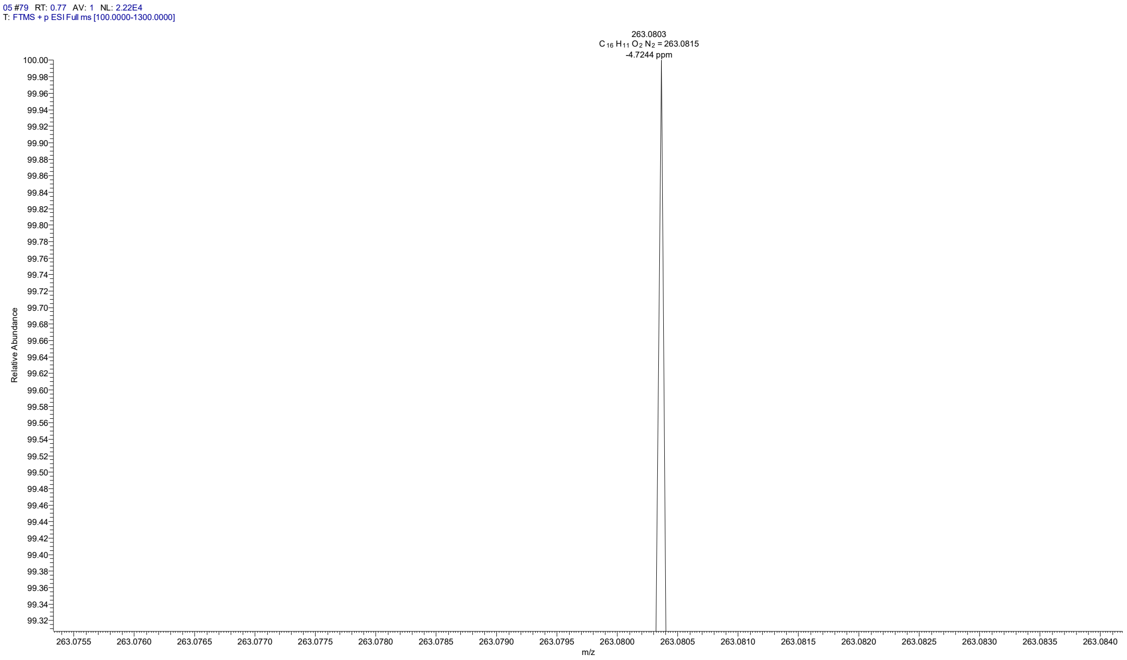

Figure S17. **A5** HRMS for $C_{16}H_{10}O_2N_2$ $[M+H]^+$. Calcd. 263.0815. Found 267.0803.

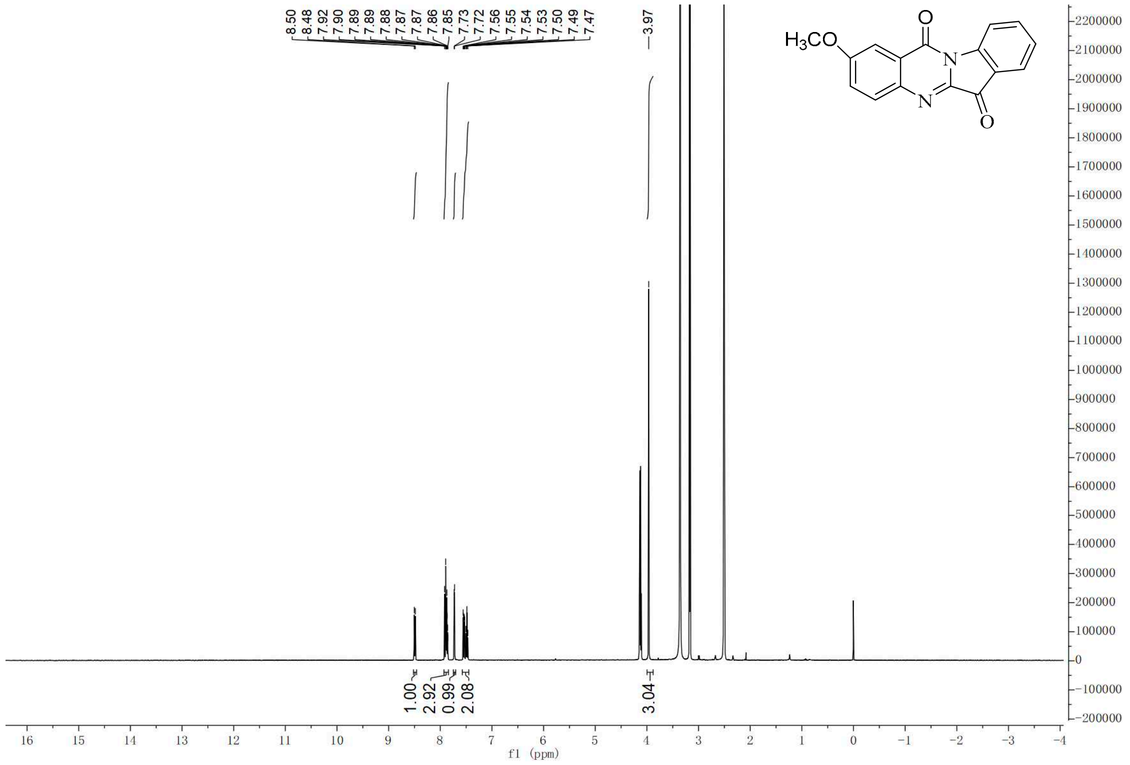

Figure S18. ^{1}H NMR Spectrum (DMSO-d_6, 400 MHz) of **A6**.

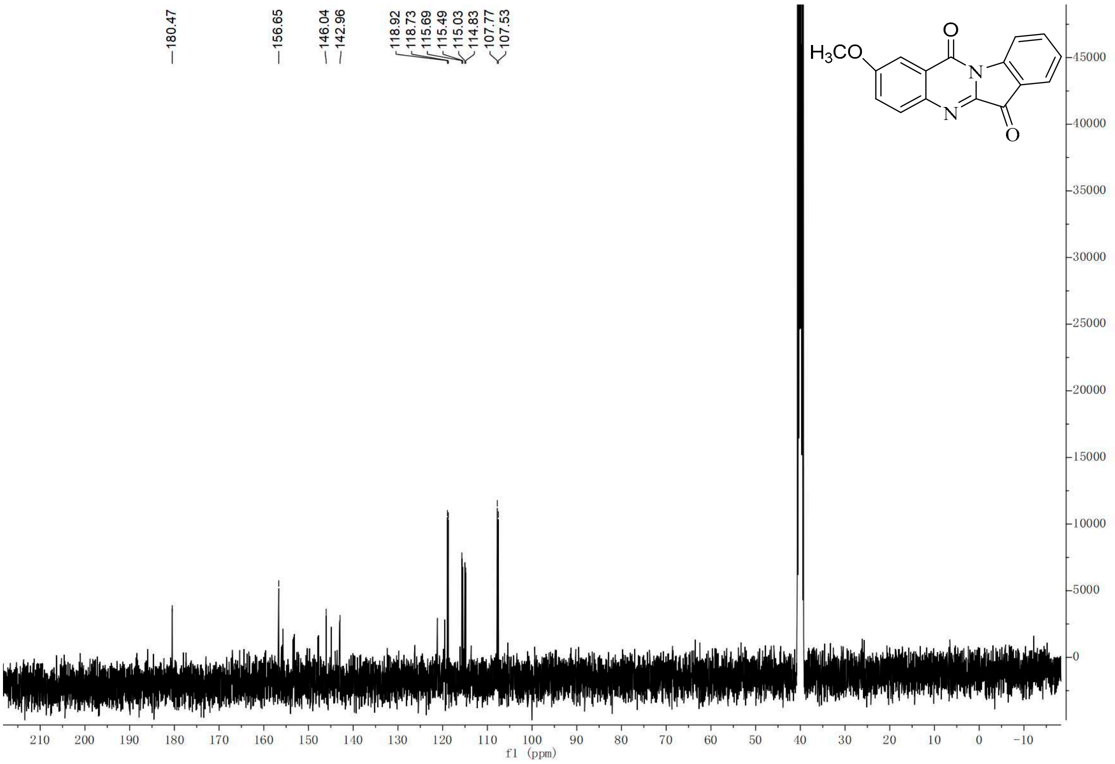

Figure S19. ^{13}C NMR Spectrum (DMSO-d_6, 101 MHz) of **A6**.

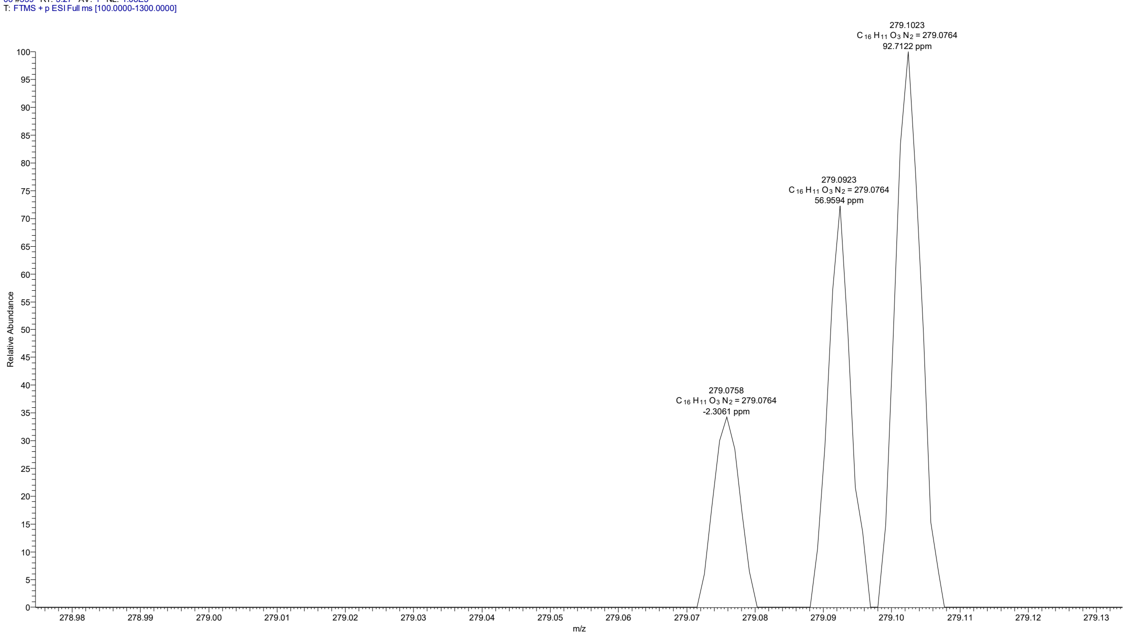

Figure S20. **A6** HRMS for $C_{16}H_{10}O_3N_2$ [M+H]$^+$. Calcd. 279.0764. Found 279.0758.

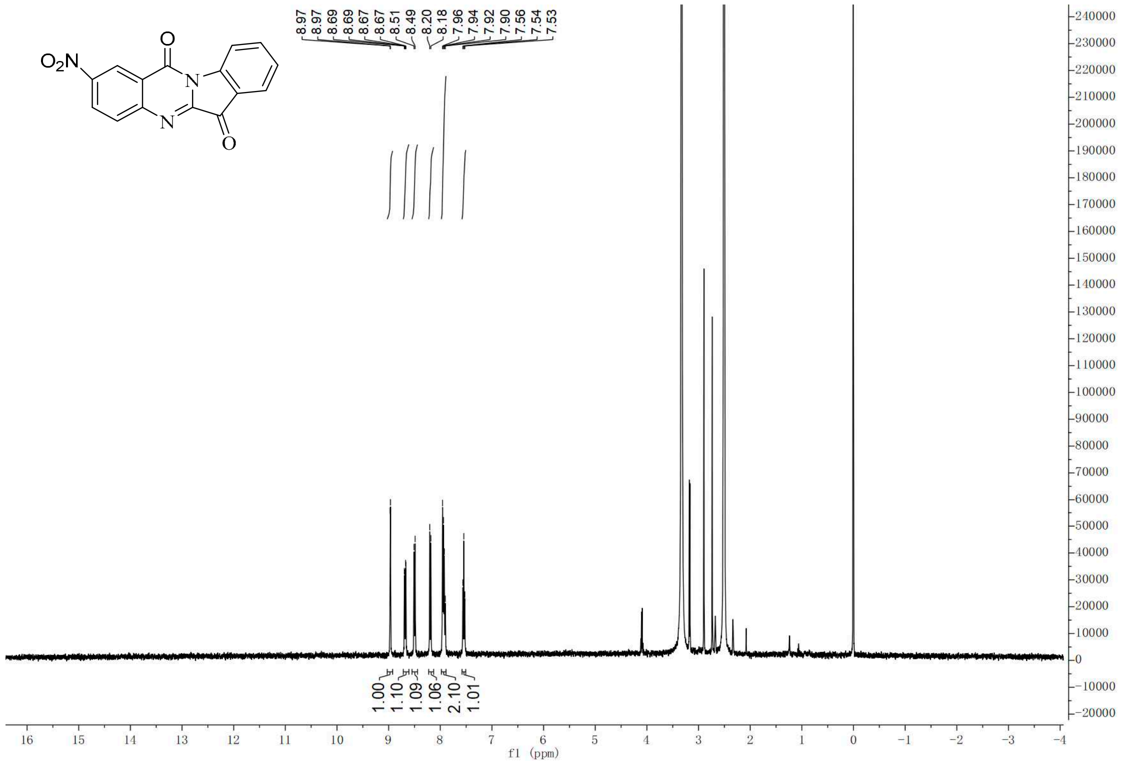

Figure S21. ^{1}H NMR Spectrum (DMSO-d_6, 400 MHz) of **A7**.

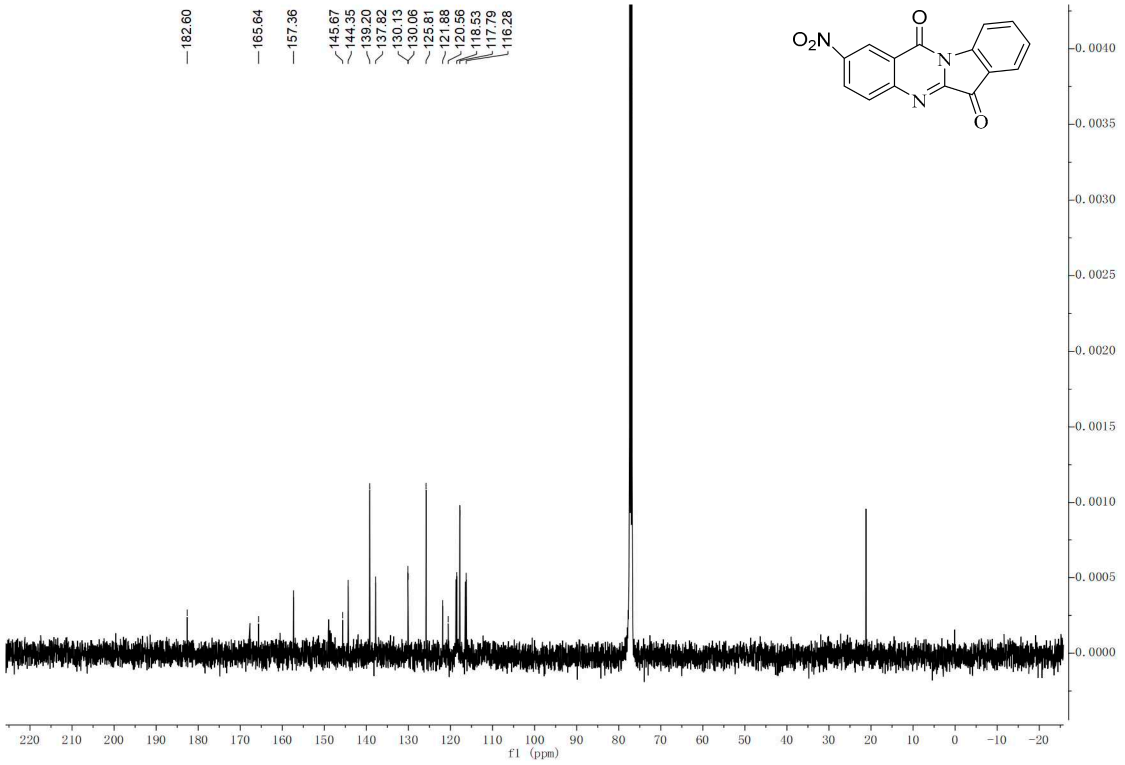

Figure S22. ^{13}C NMR Spectrum (DMSO-d_6, 101 MHz) of **A7**.

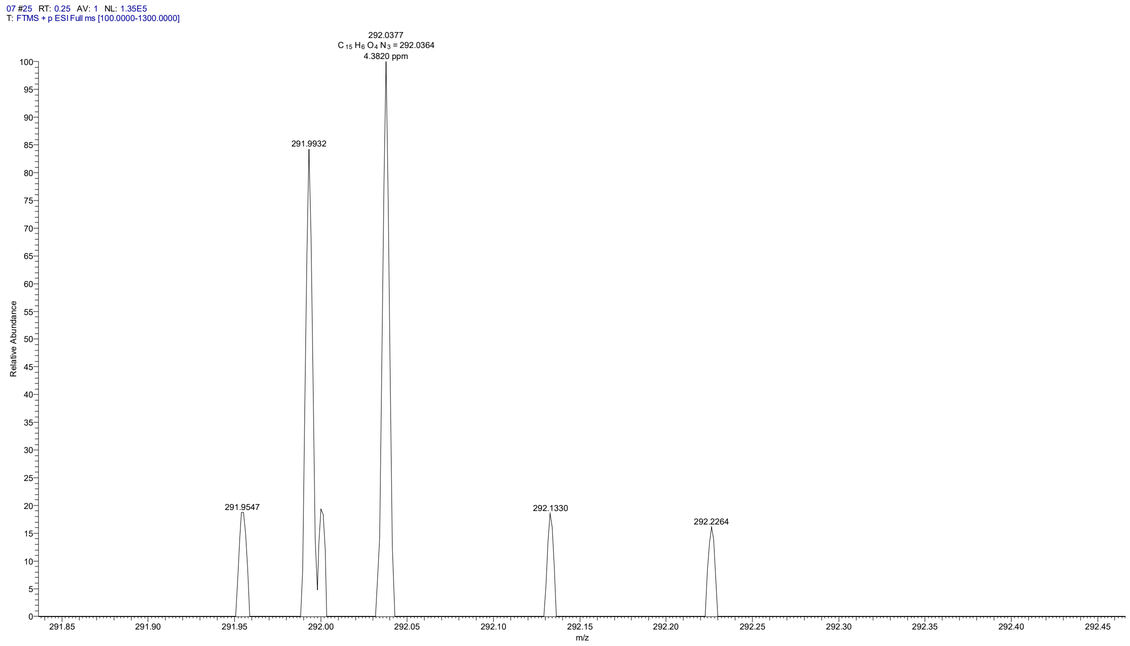

Figure S23. A7 HRMS for $C_{15}H_7O_4N_3$ $[M-H]^-$. Calcd. 292.0364. Found 292.0367.

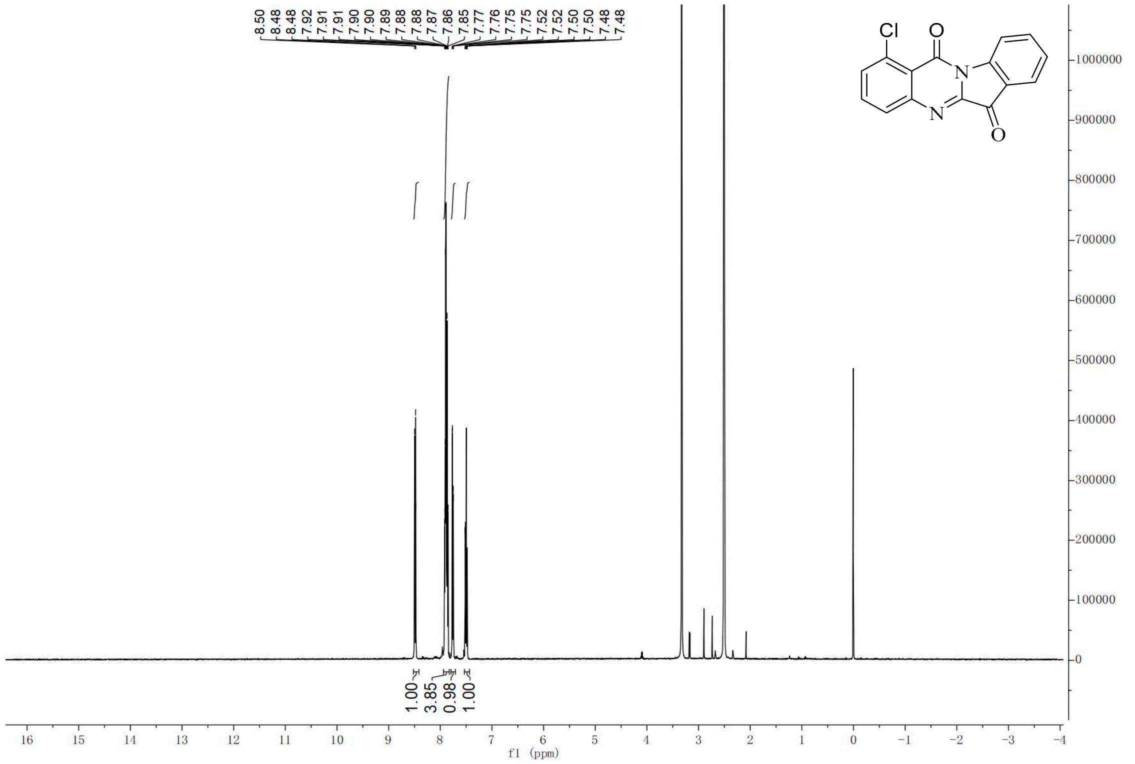

Figure S24. ^{1}H NMR Spectrum (DMSO-d_6, 500 MHz) of **A8**.

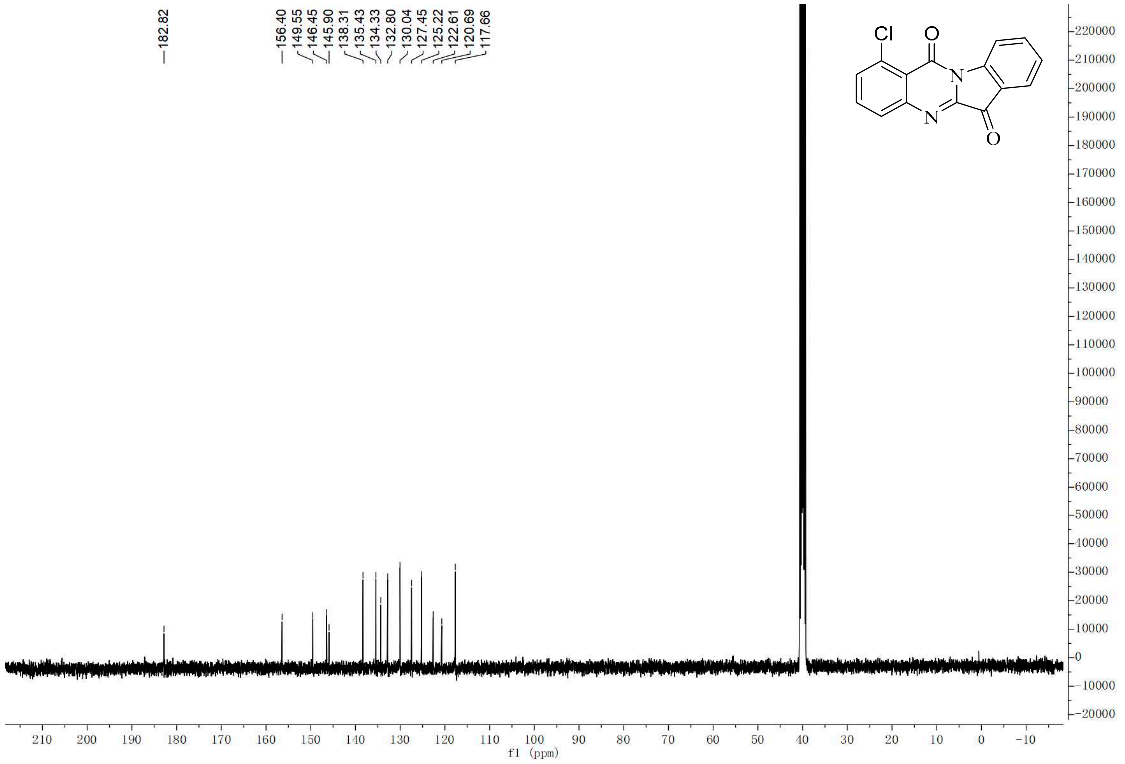

Figure S25. ^{13}C NMR Spectrum (DMSO-d_6, 101 MHz) of **A8**.

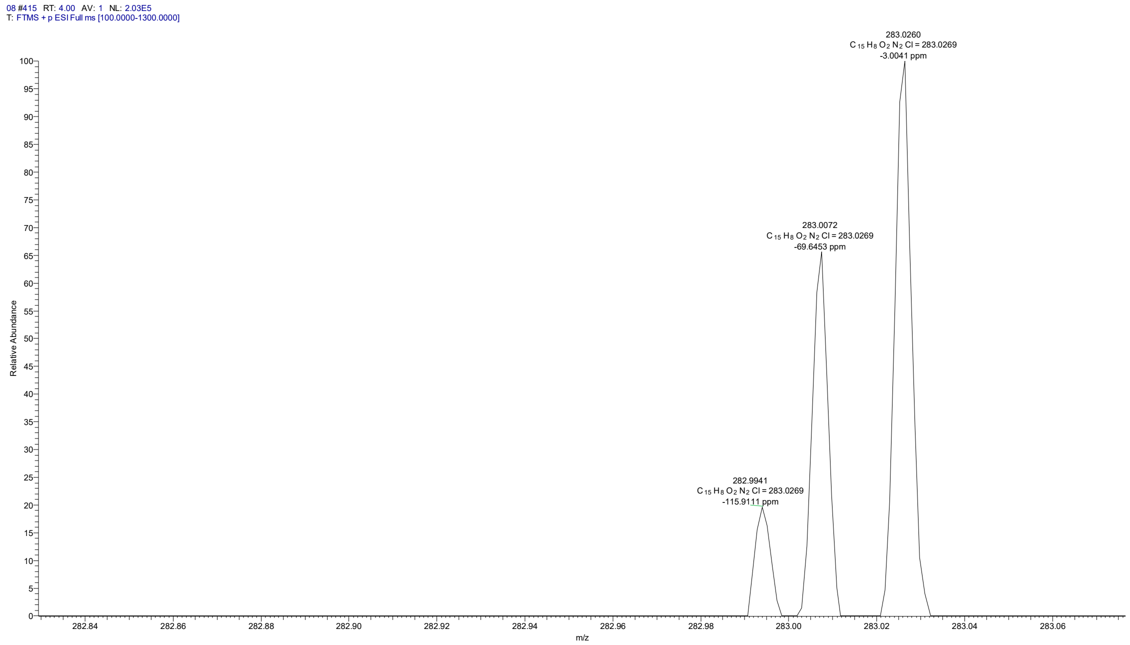

Figure S26. **A8** HRMS for $C_{15}H_7ClN_2O_2$ $[M+H]^+$. Calcd. 283.0269. Found 283.0260.

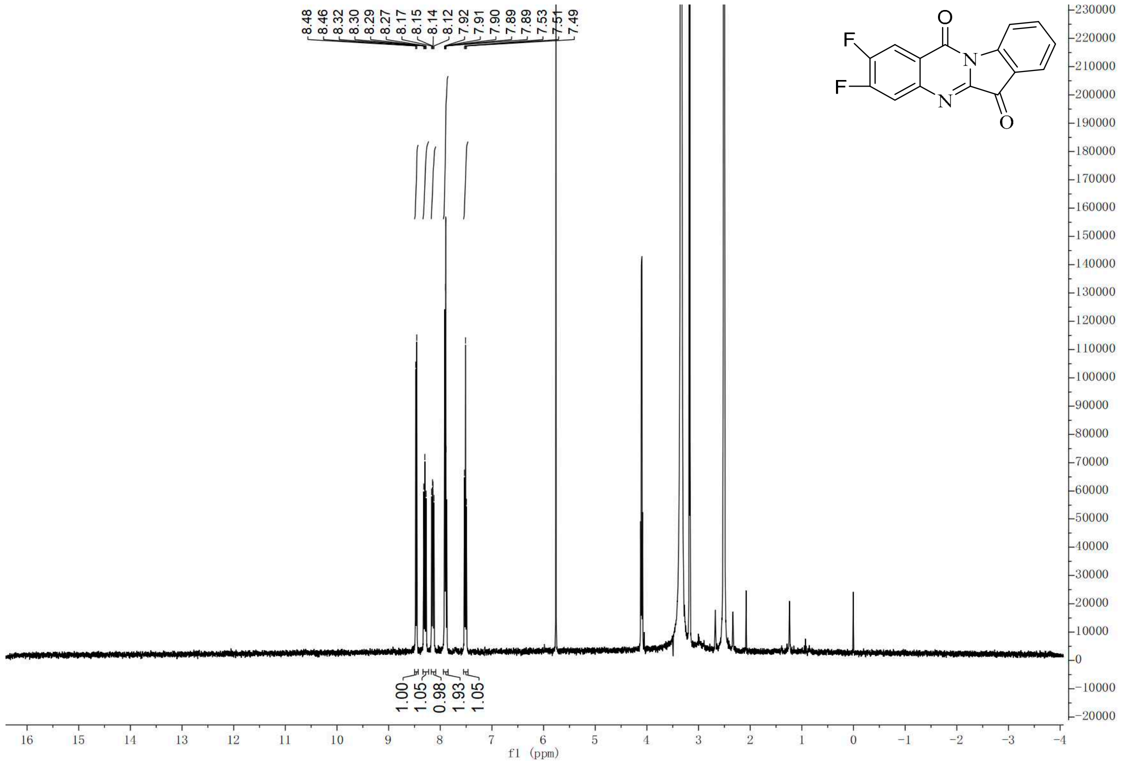

Figure S27. ^{1}H NMR Spectrum (DMSO-*d6*, 400 MHz) of **A9**.

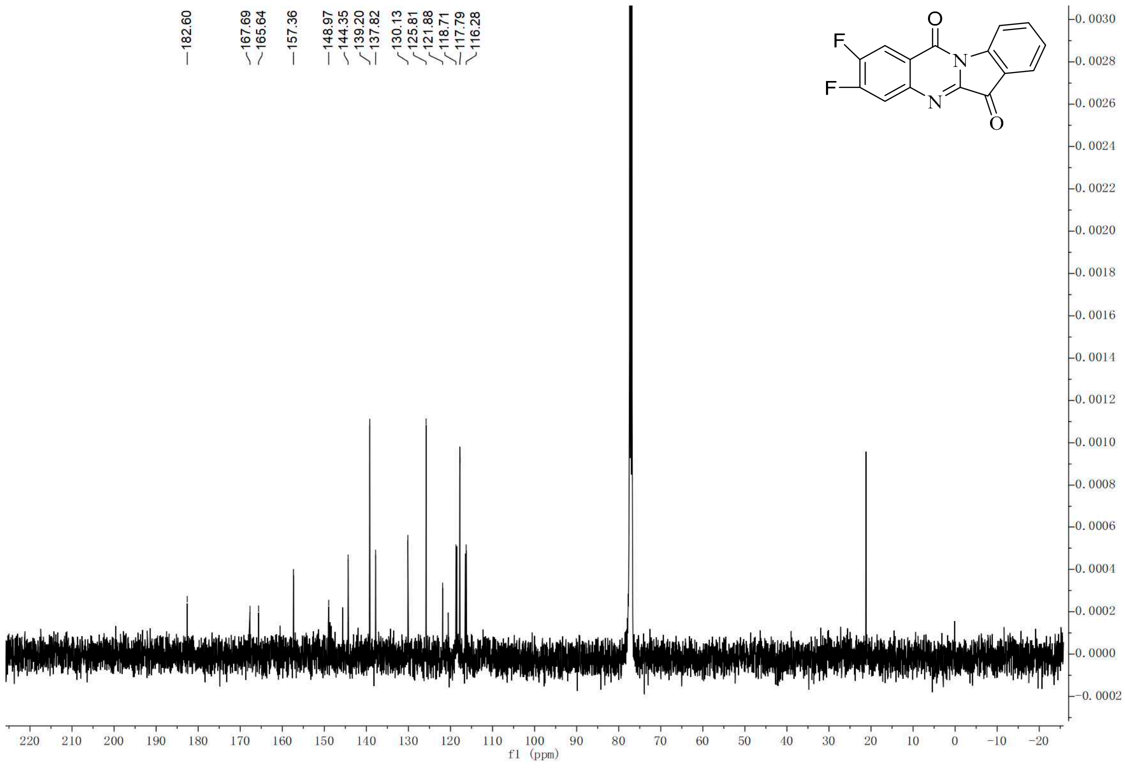

Figure S28. ^{13}C NMR Spectrum (DMSO-*d6*, 101 MHz) of **A9**.

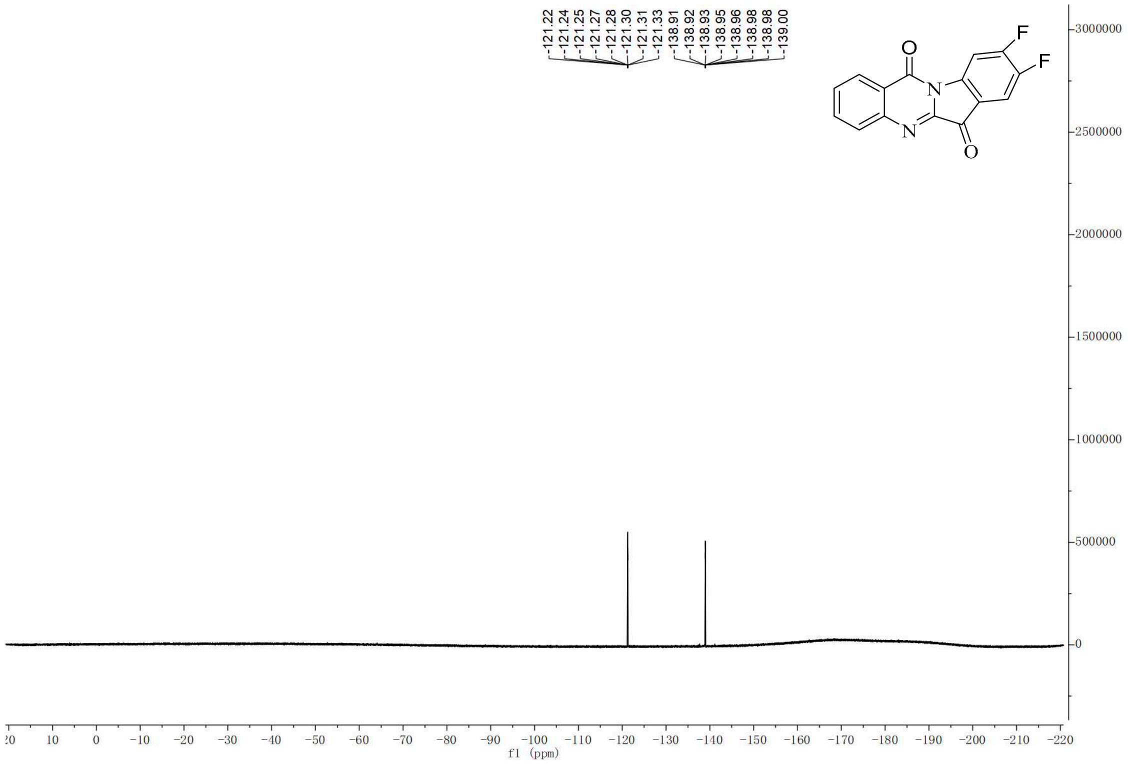

Figure S29. ^{19}F NMR Spectrum (DMSO-*d*$_6$, 376 MHz) of **A9**.

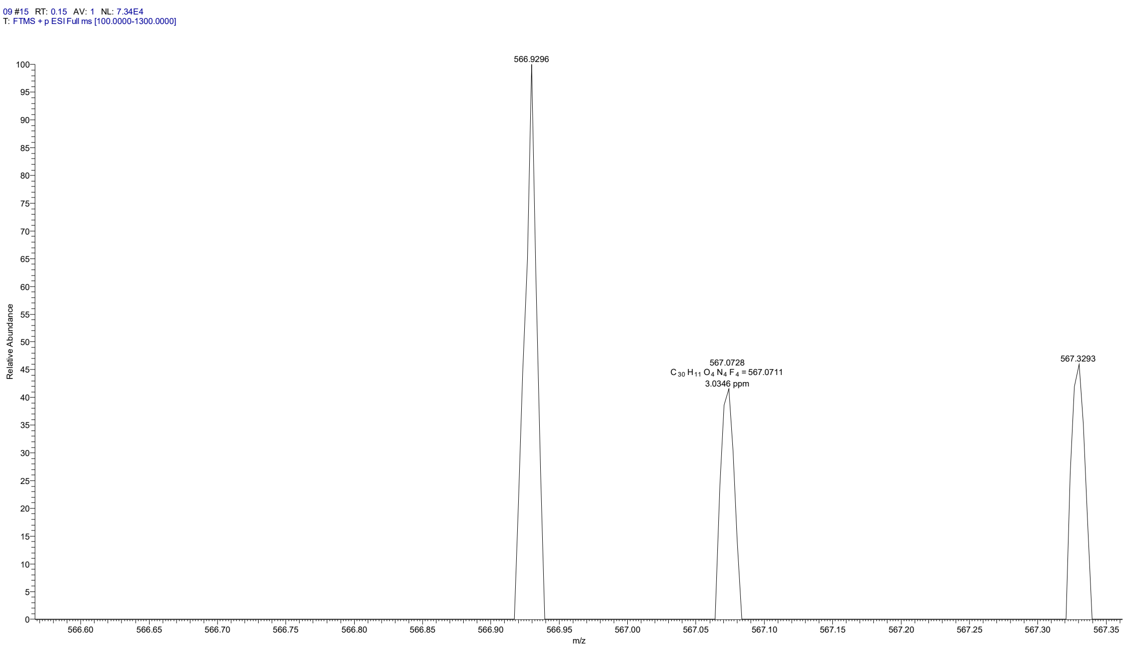

Figure S30. **A9** HRMS for $C_{15}H_6F_2N_2O_2$ [2M-H]$^-$. Calcd. 567.0711. Found 567.0728.

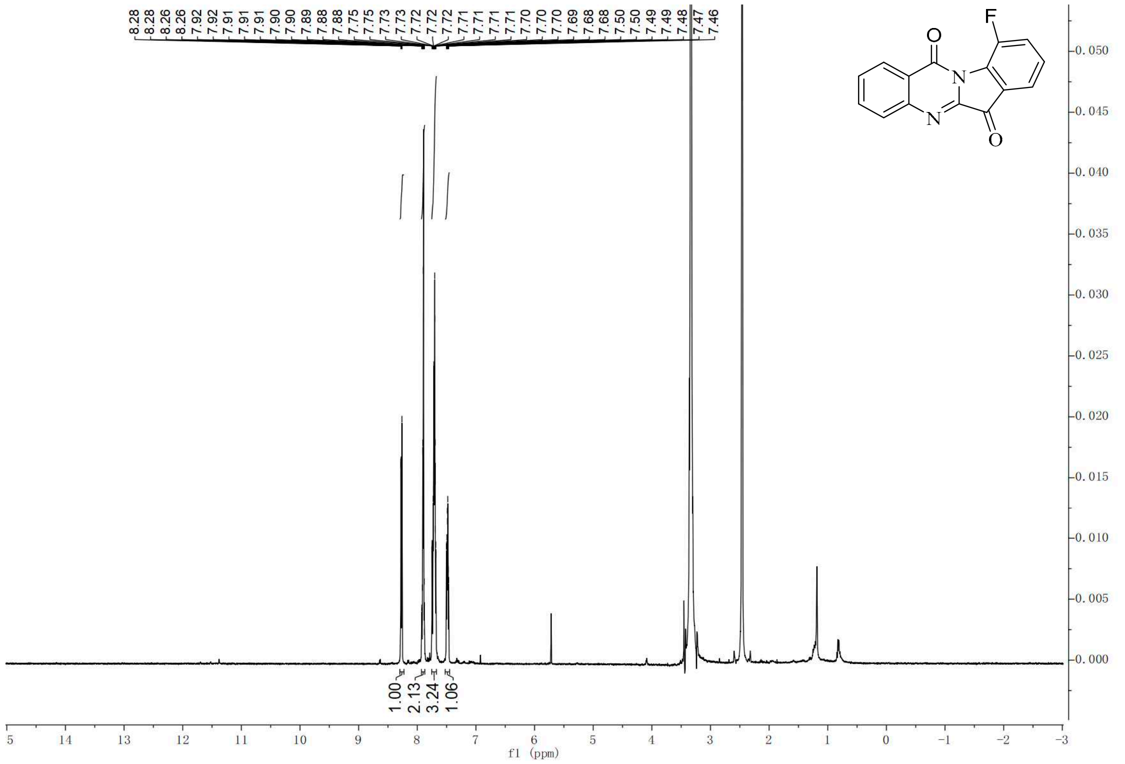

Figure S31. ^{1}H NMR Spectrum (DMSO-d_6, 400 MHz) of **B1**.

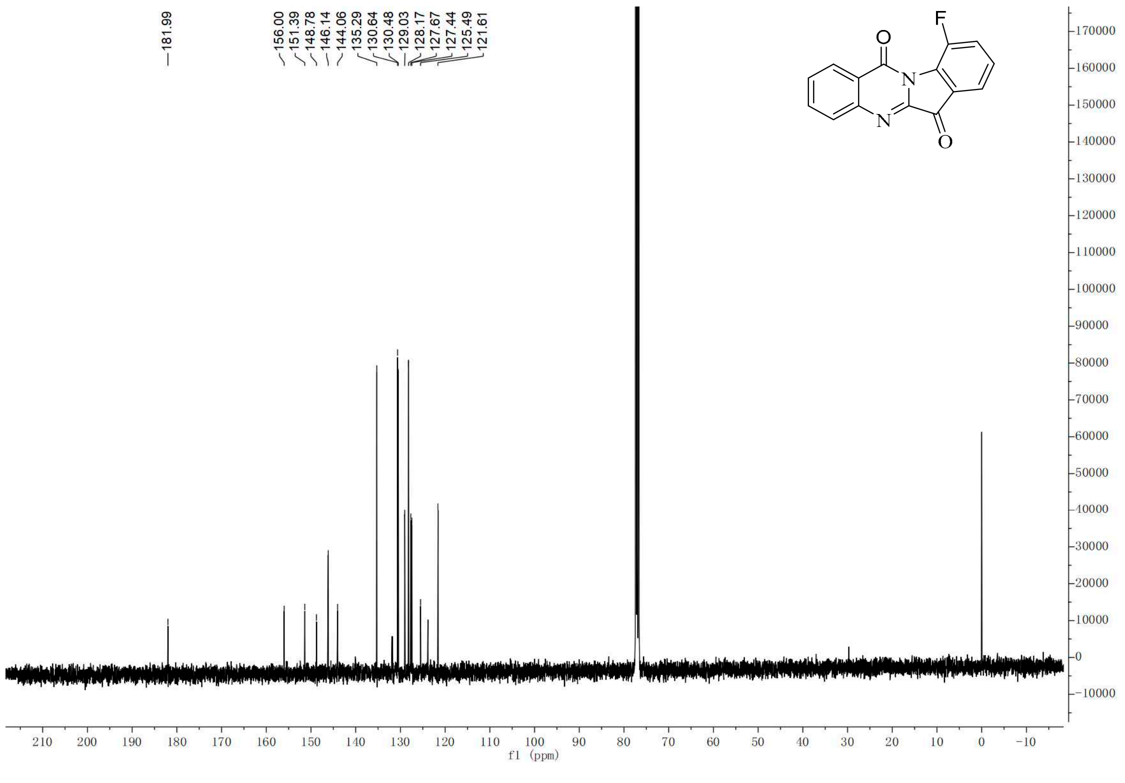

Figure S32. ^{13}C NMR Spectrum (Chloroform-d, 101 MHz) of **B1**.

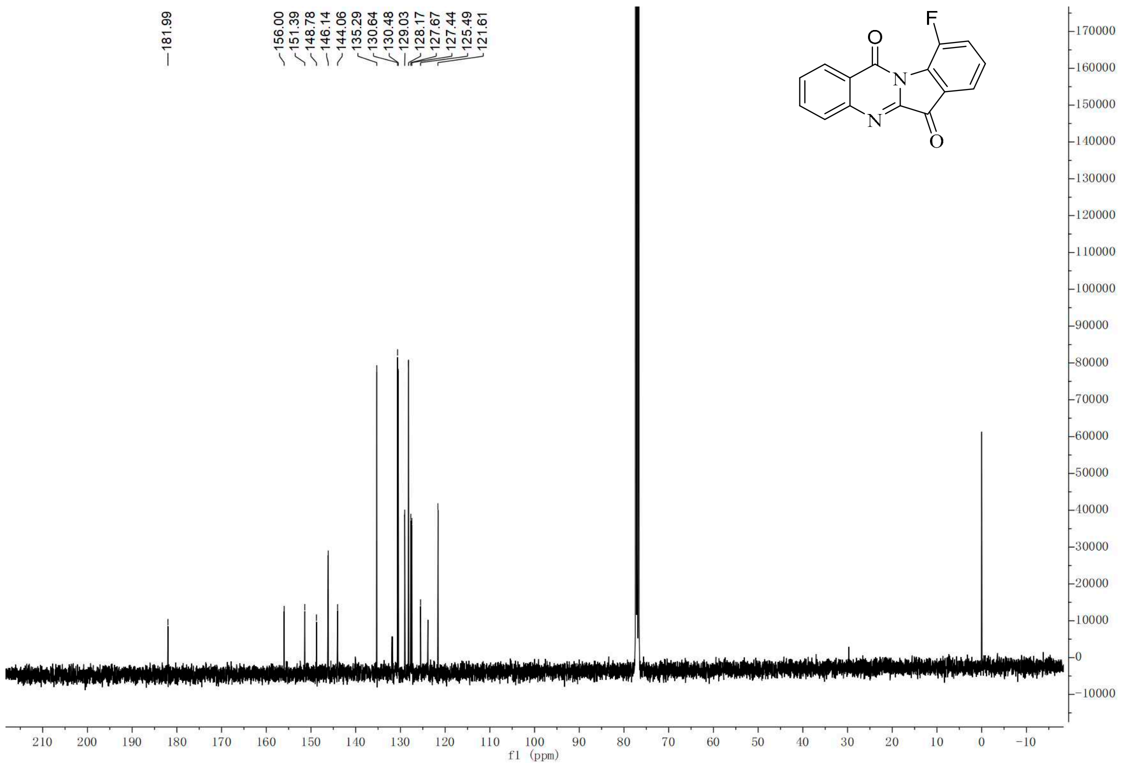

Figure S33. ^{19}F NMR Spectrum (DMSO-d_6, 376 MHz) of **B1**.

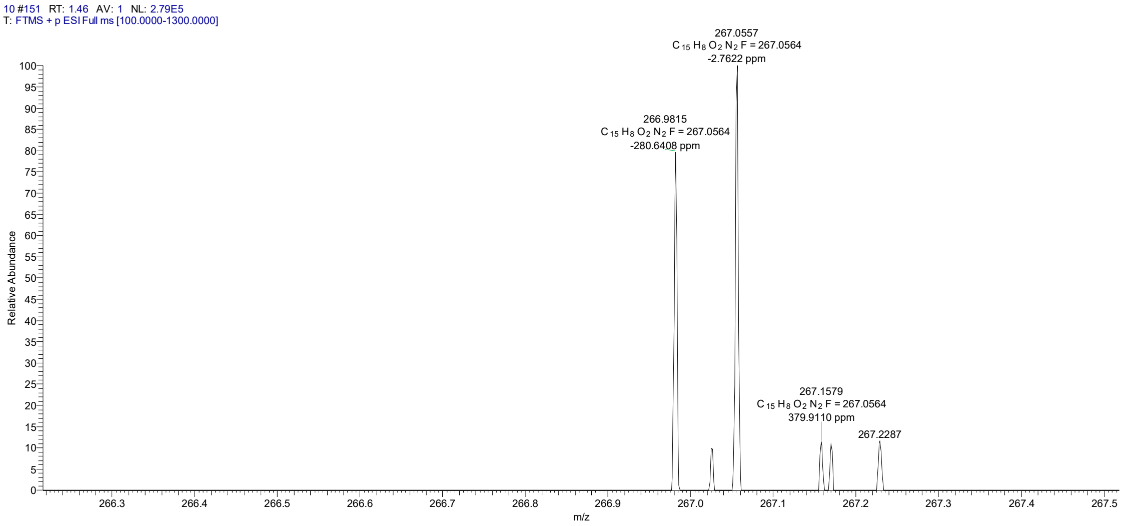

Figure S34. **B1** HRMS for $C_{15}H_7O_2N_2F$ $[M+H]^+$. Calcd. 267.0564. Found 267.0557.

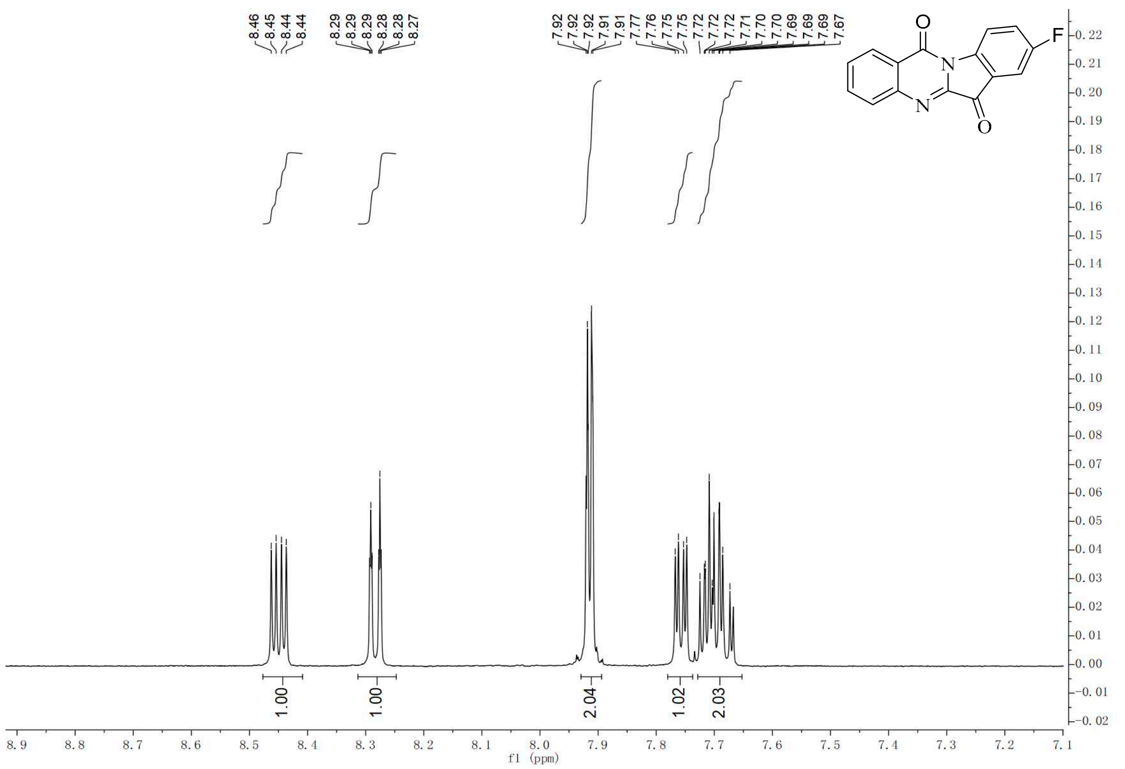

Figure S35. ^{1}H NMR Spectrum (DMSO-d_6, 400 MHz) of **B2**.

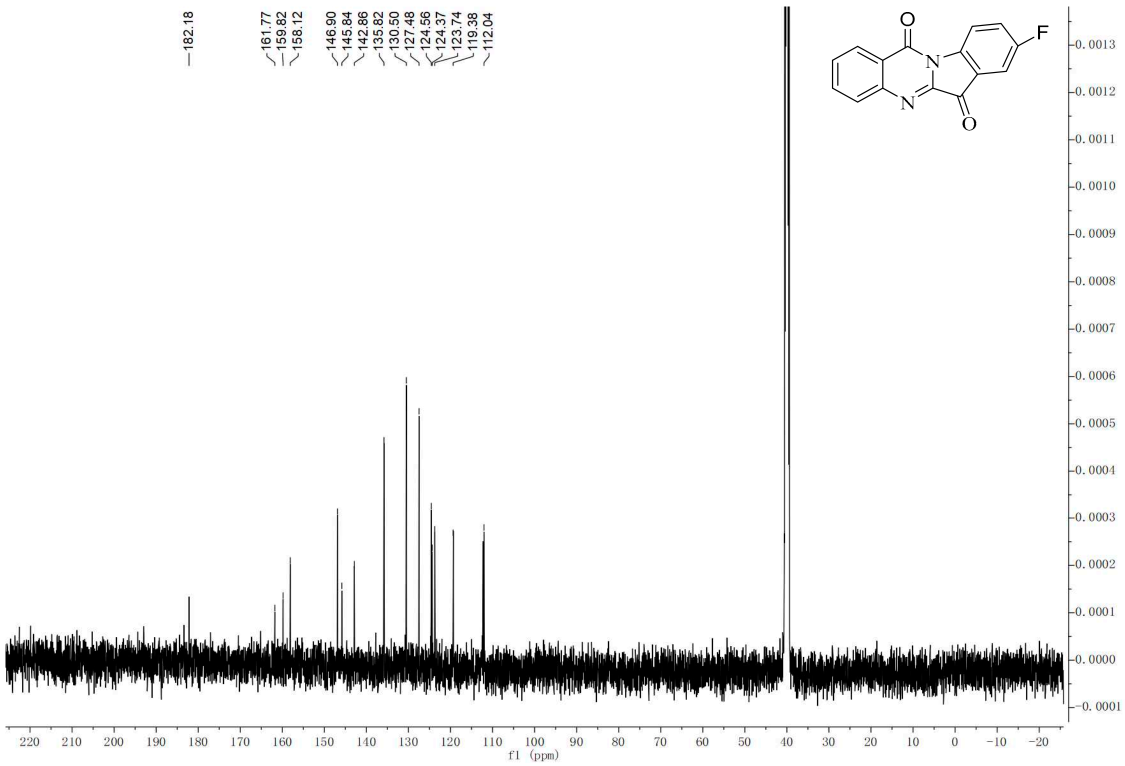

Figure S36. ^{13}C NMR Spectrum (DMSO-d_6, 101 MHz) of **B2**.

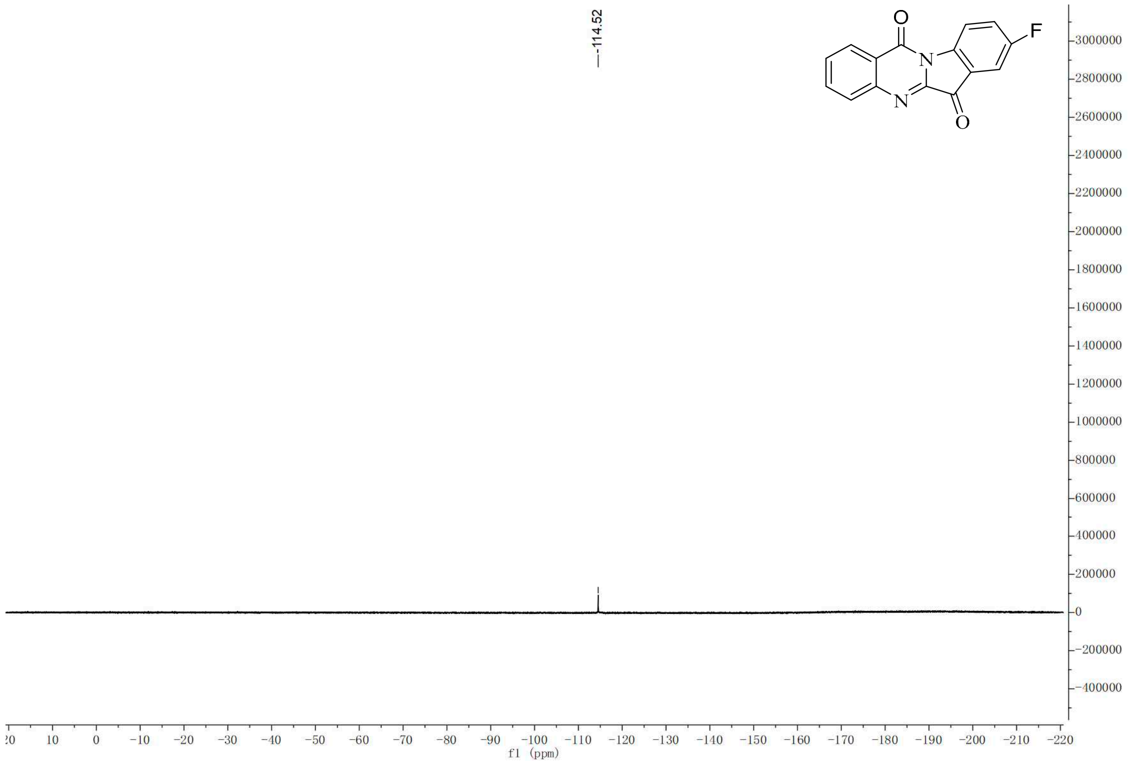

Figure S37. ^{19}F NMR Spectrum (DMSO-d_6, 376 MHz) of **B2**.

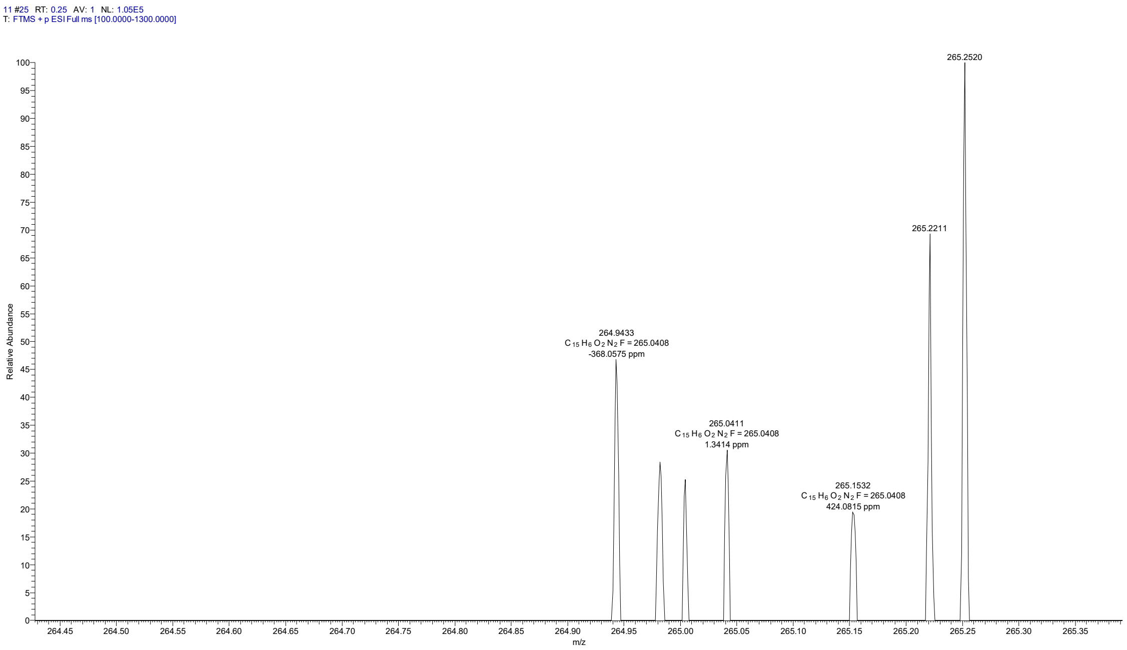

Figure S38. **B2** HRMS for $C_{15}H_7O_2N_2F$ [M-H]$^-$. Calcd. 265.0408. Found 265.0411.

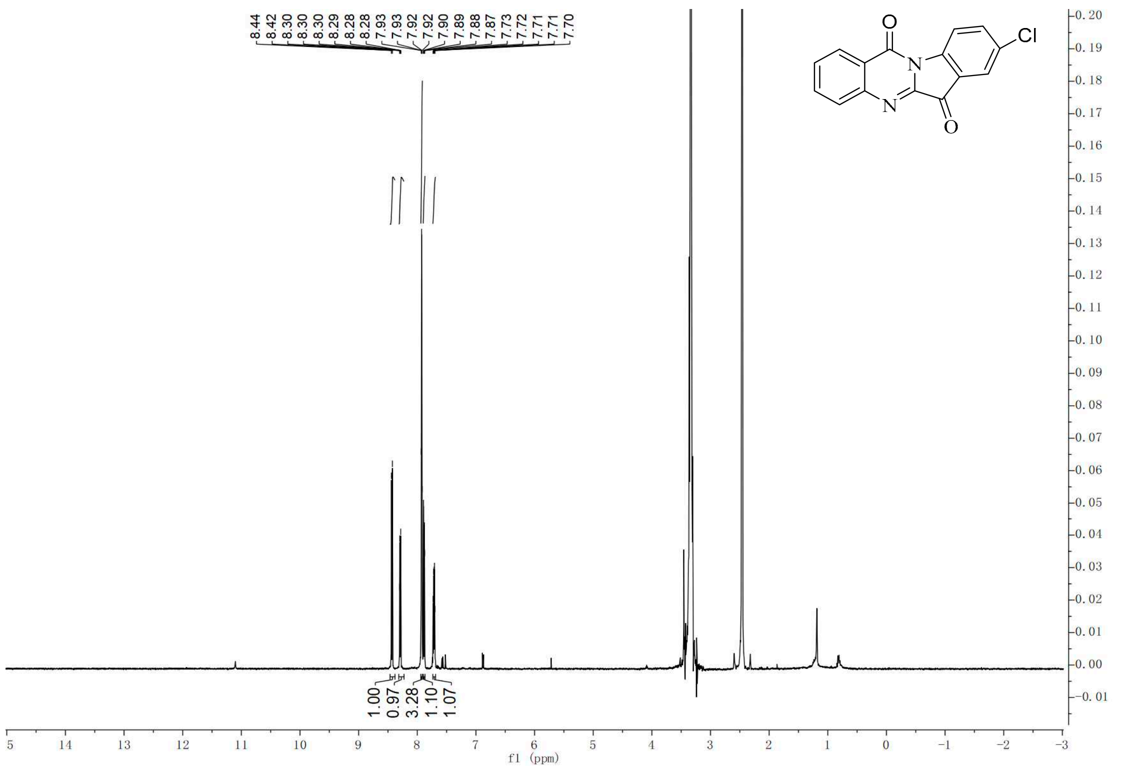

Figure S39. ^{1}H NMR Spectrum (DMSO-d_6, 400 MHz) of **B3**.

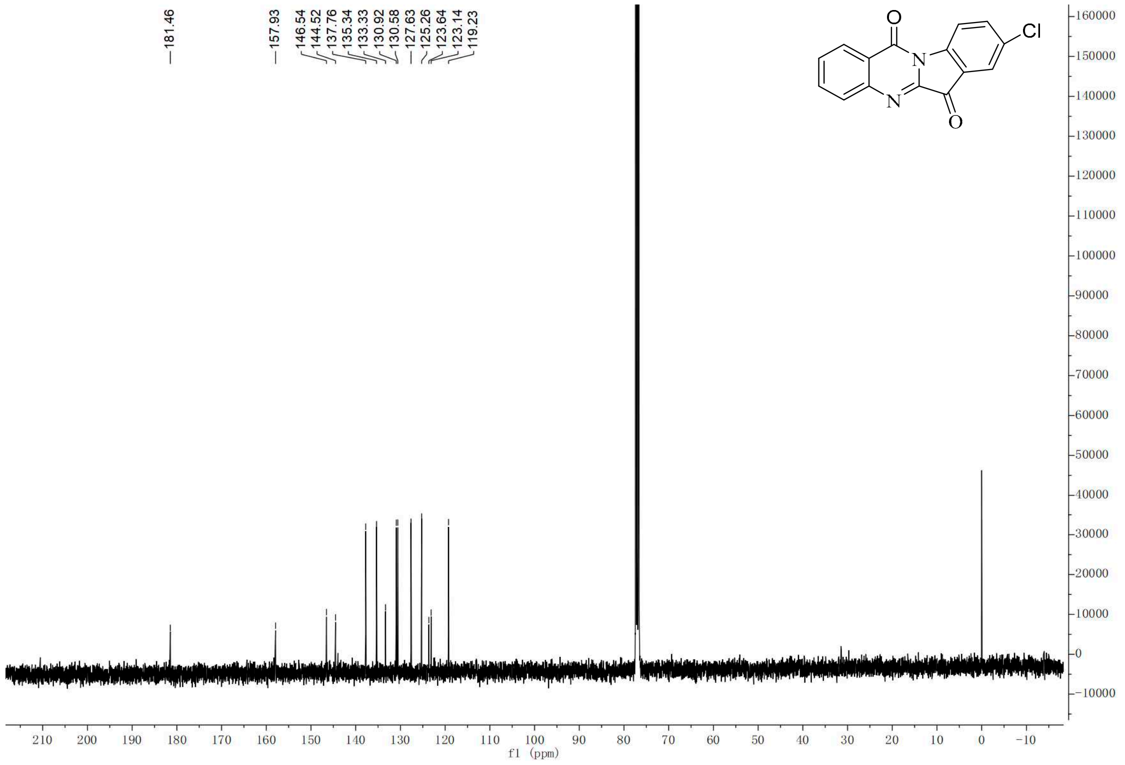

Figure S40. ^{13}C NMR Spectrum (Chloroform-*d*, 101 MHz) of **B3**.

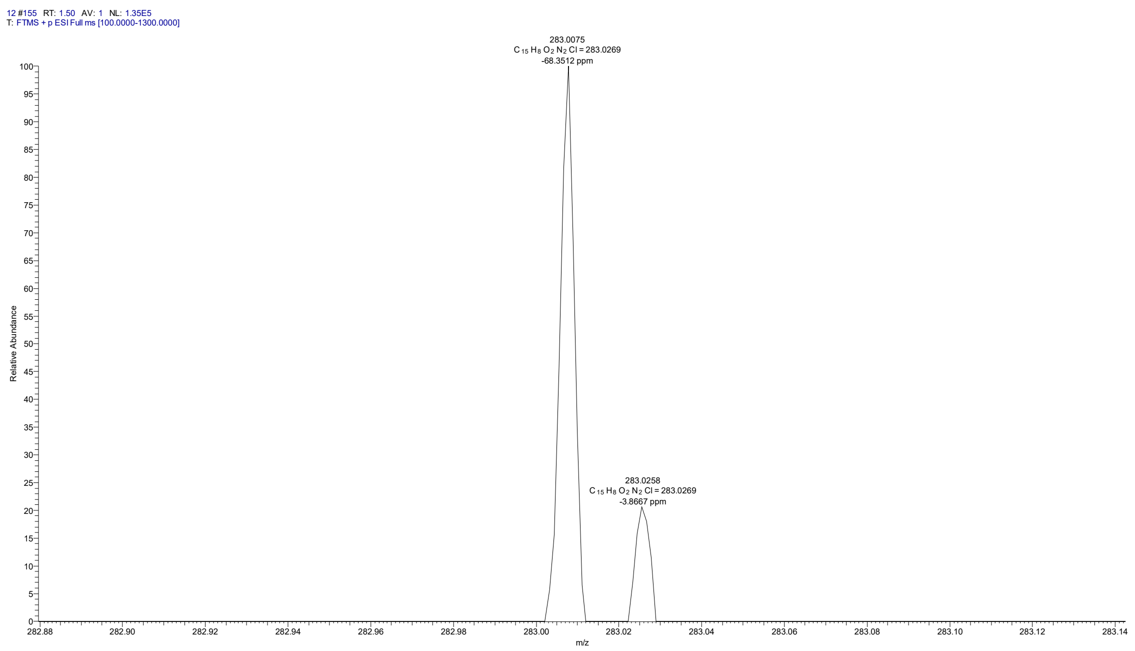

Figure S41. **B3** HRMS for $C_{15}H_7O_2N_2Cl$ $[M+H]^+$. Calcd. 283.0269. Found 283.0258.

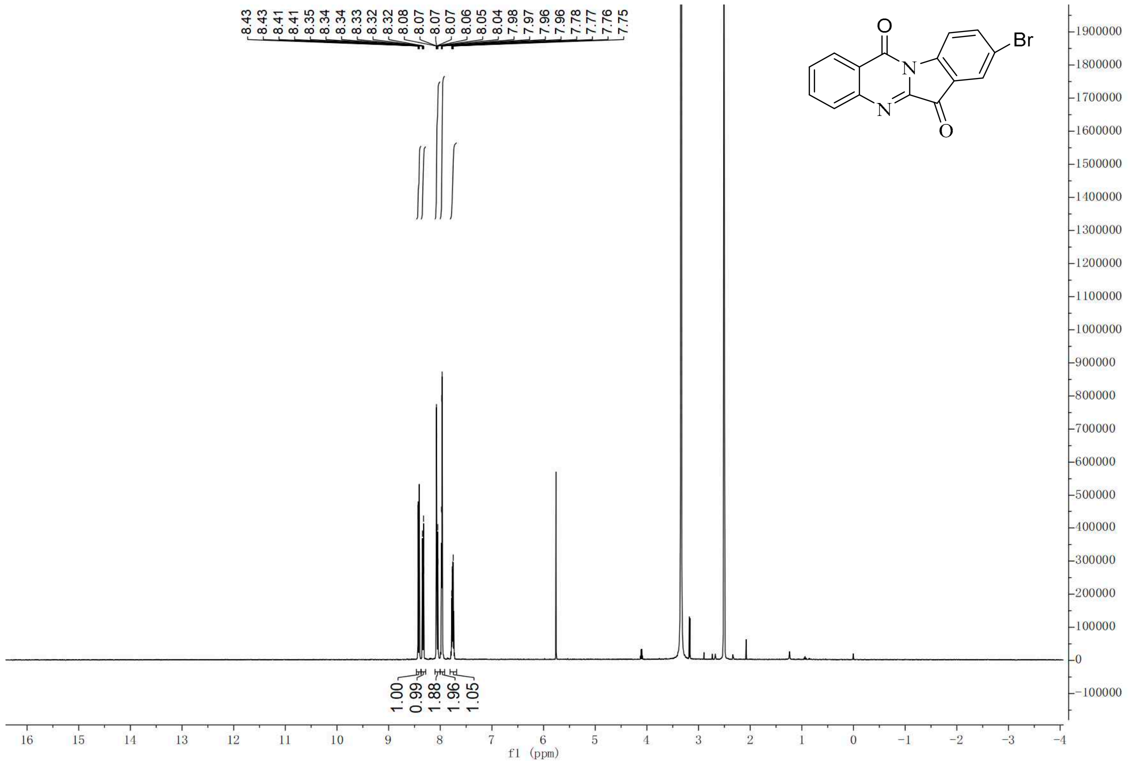

Figure S42. ^1H NMR Spectrum (DMSO-d_6, 400 MHz) of **B4**.

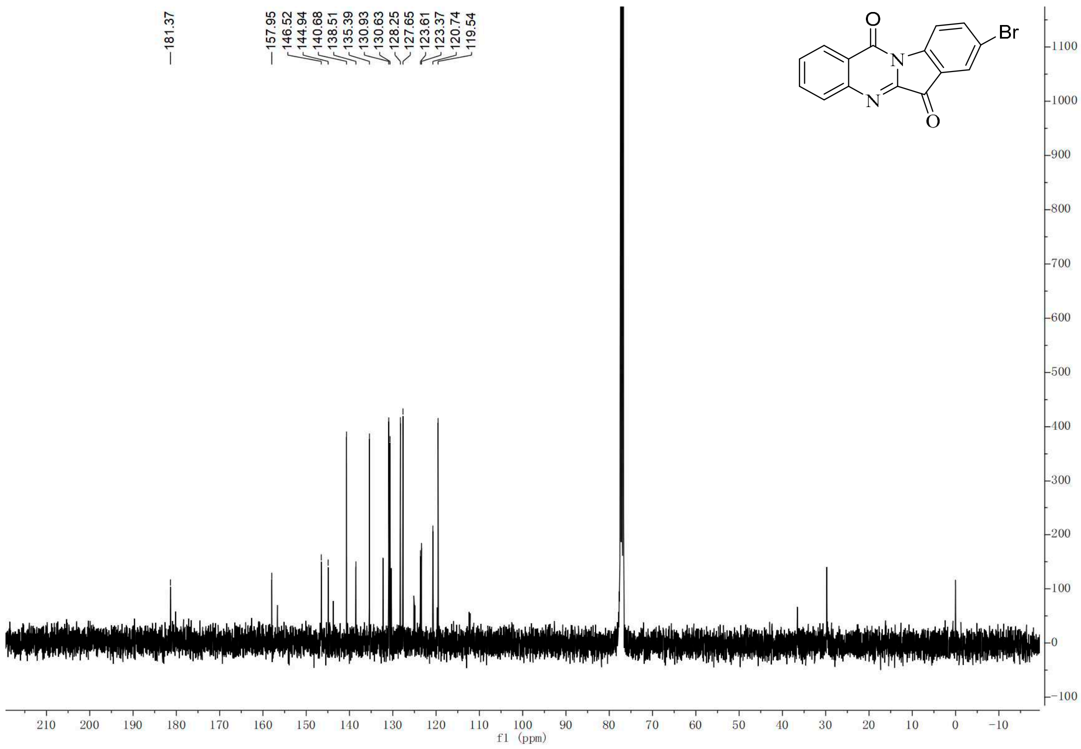

Figure S43. ^{13}C NMR Spectrum (CDCl3-*d*6, 101 MHz) of **B4**.

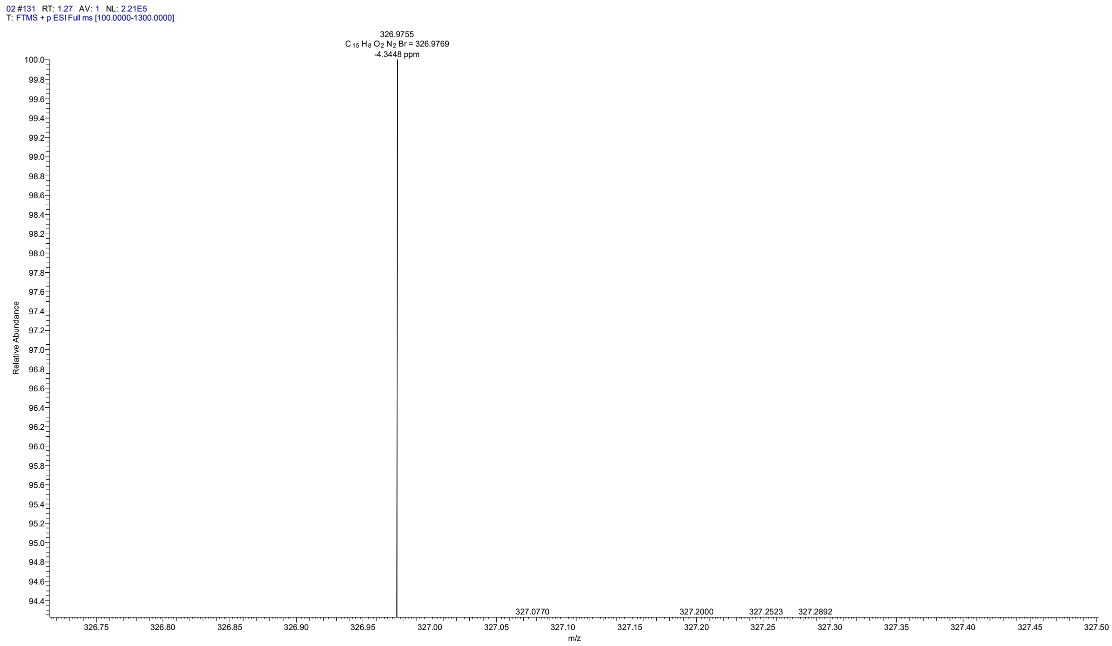

Figure S44. **B4** HRMS for $C_{15}H_7O_2N_2Br$ $[M+H]^+$. Calcd. 326.9769. Found 326.9755.

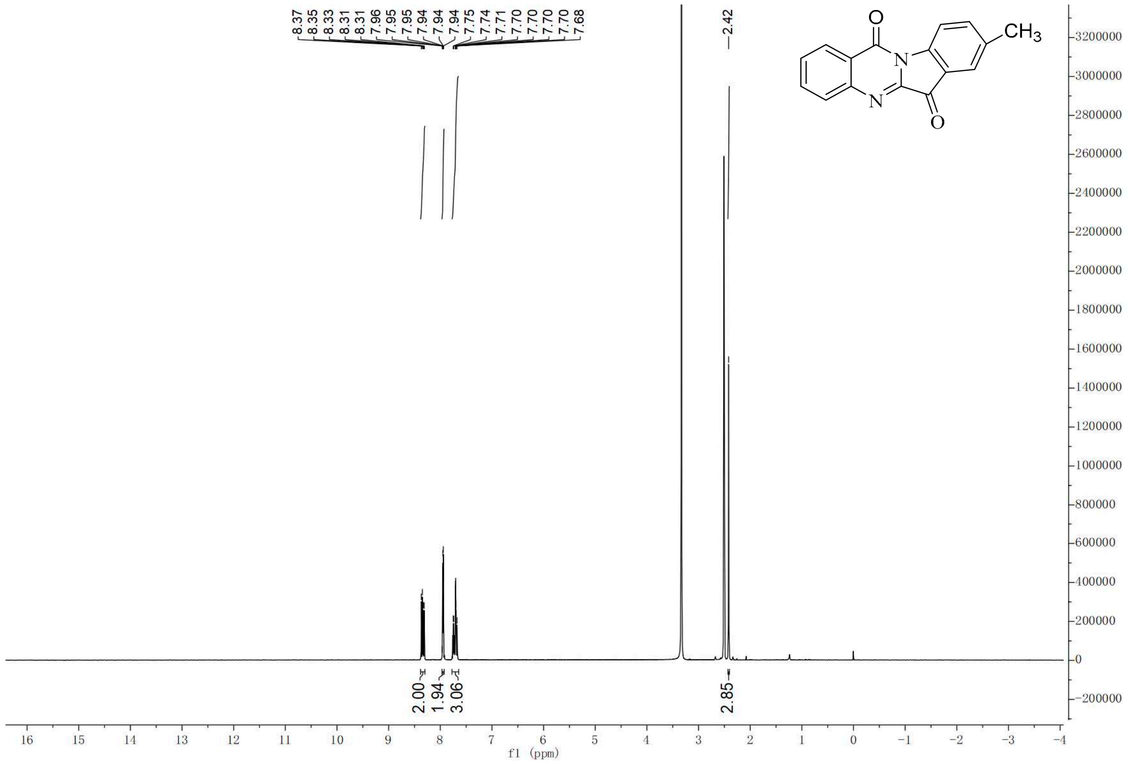

Figure S45. ^{1}H NMR Spectrum (DMSO-d_6, 400 MHz) of **B5**.

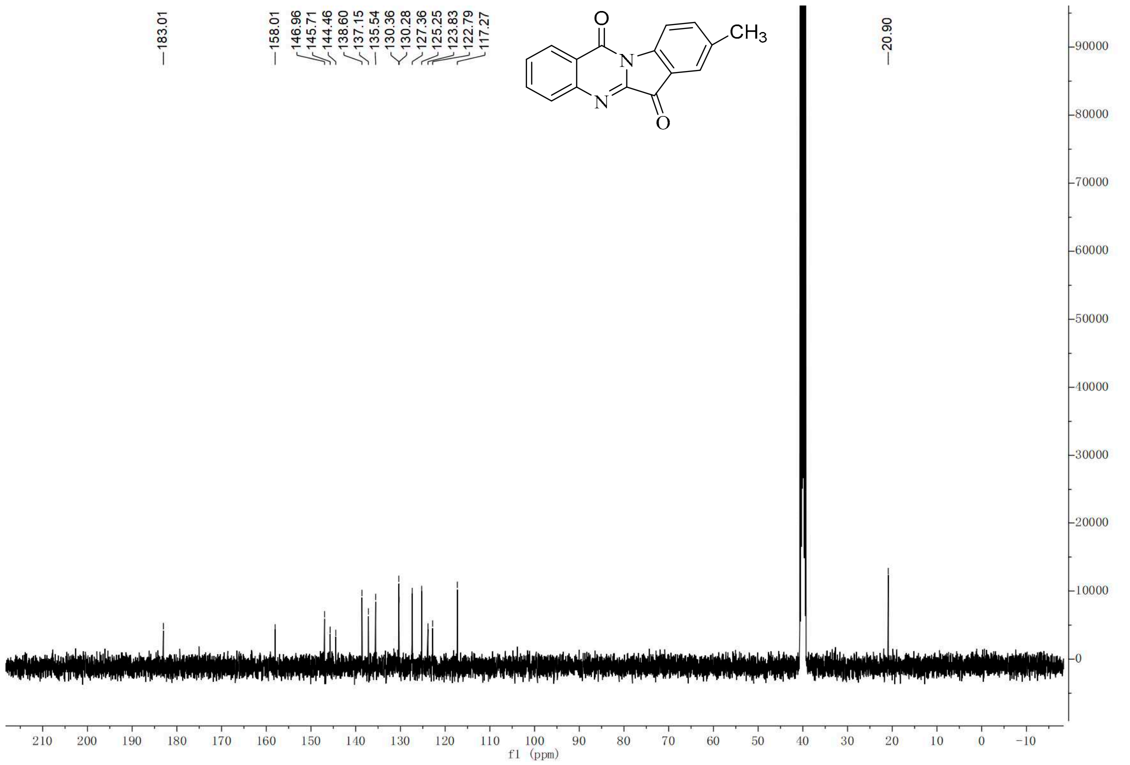

Figure S46. ^{13}C NMR Spectrum (DMSO-d_6, 101 MHz) of **B5**.

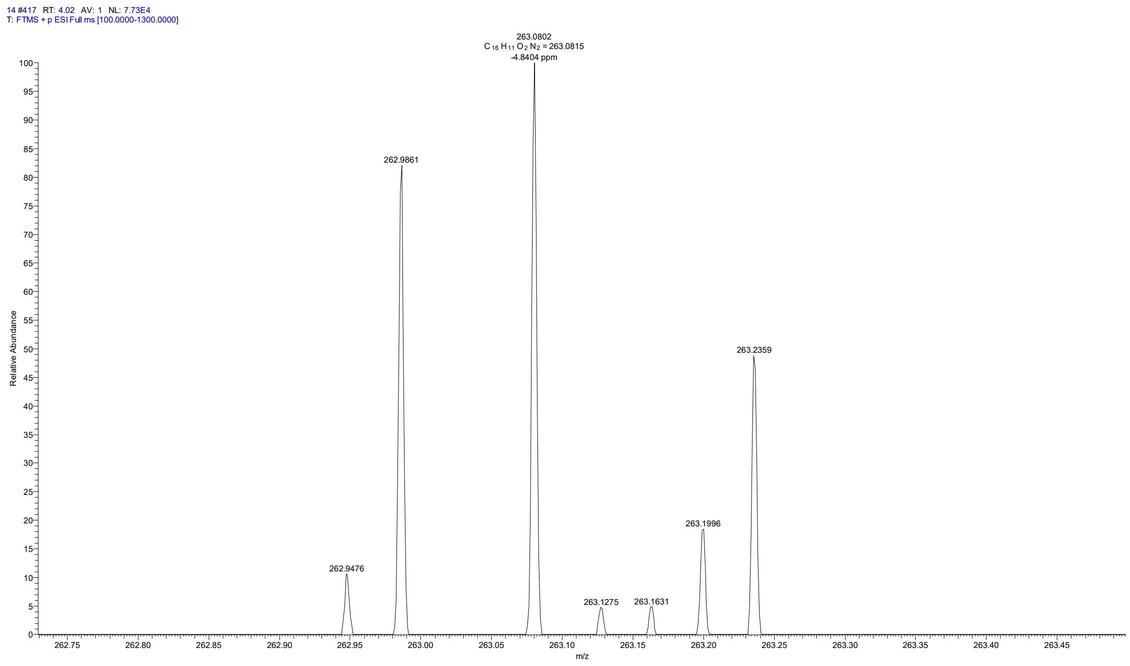

Figure S47. **B5** HRMS for $C_{16}H_{10}O_2N_2$ $[M+H]^+$. Calcd. 263.0815. Found 263.0802.

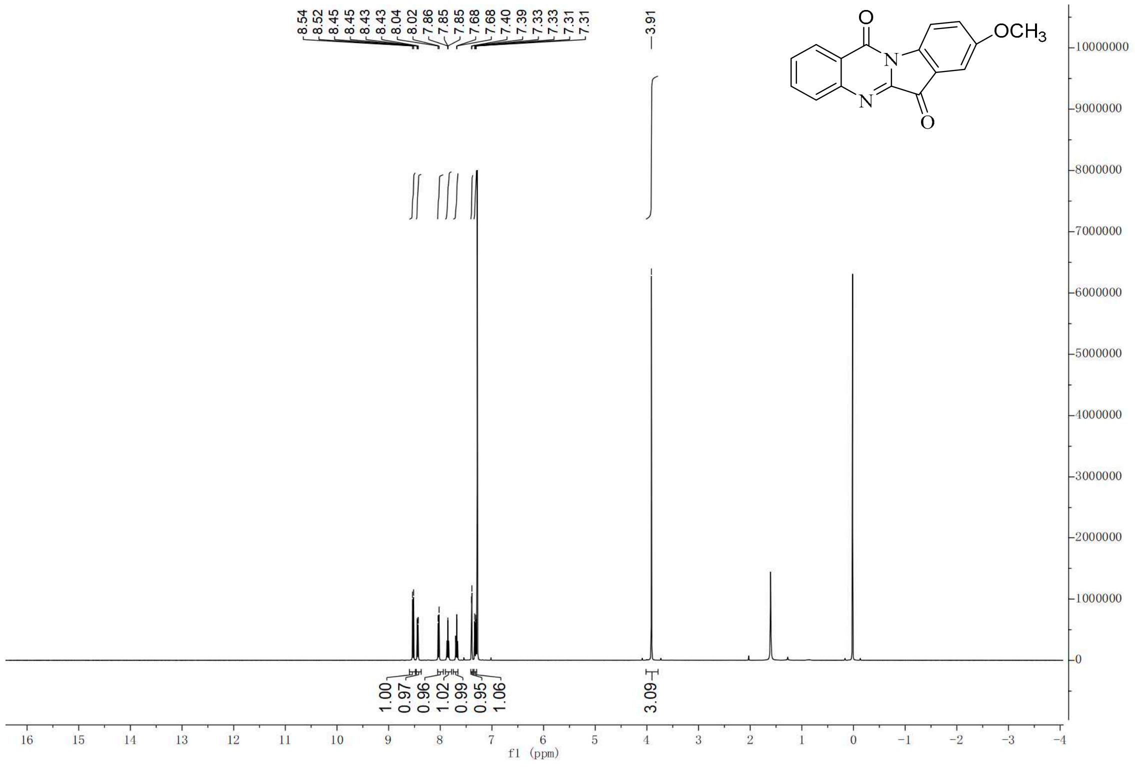

Figure S48. ^{1}H NMR Spectrum (CDCl3-*d*6, 400 MHz) of **B6**.

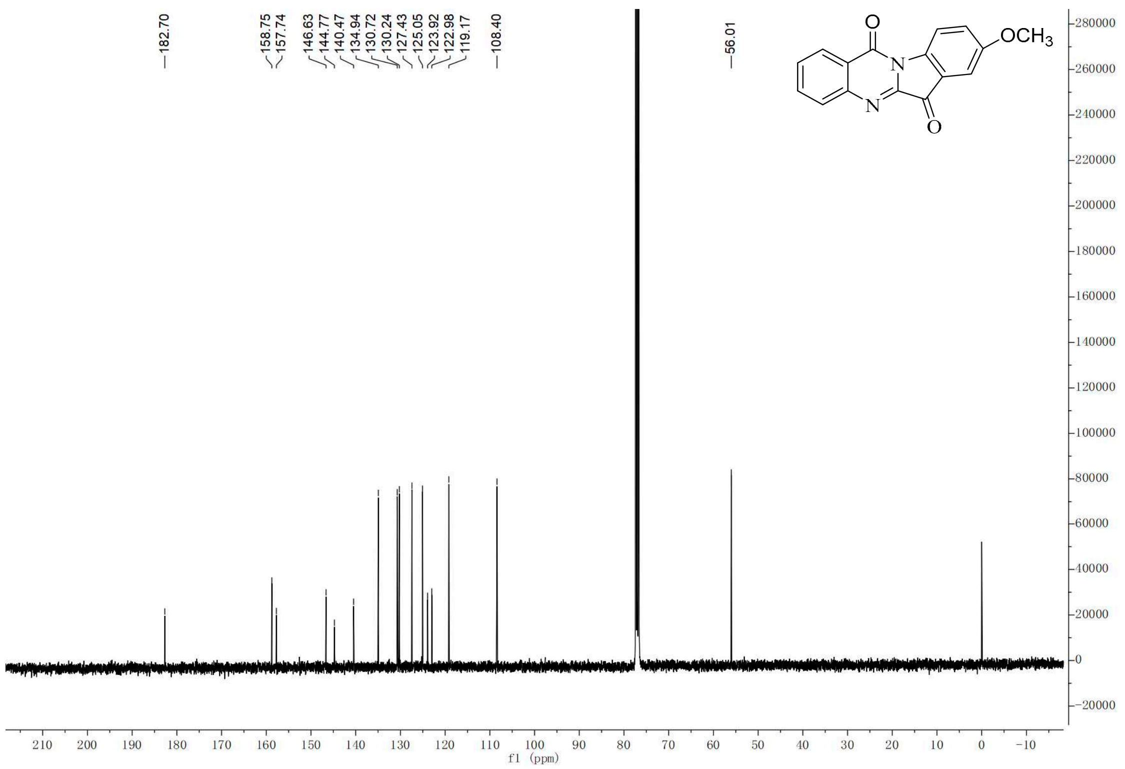

Figure S49. ^{13}C NMR Spectrum (CDCl3-*d6*, 101 MHz) of **B6**.

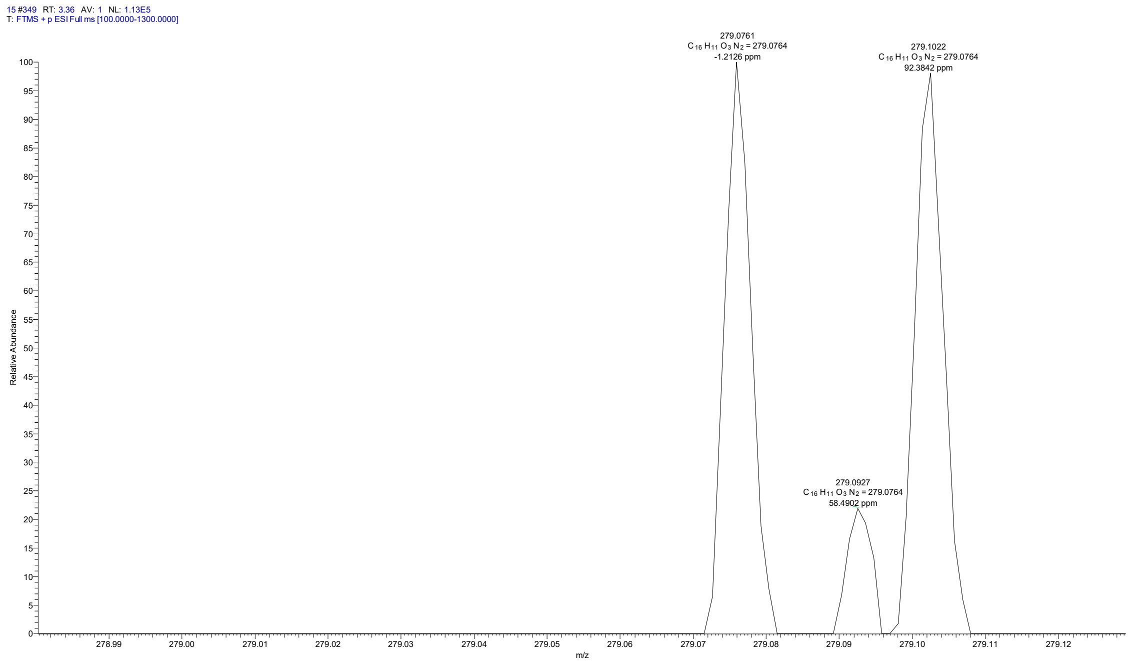

Figure S50. **B6** HRMS for $C_{16}H_{10}N_2O_3$ $[M+H]^+$. Calcd. 279.0764. Found 279.0761.

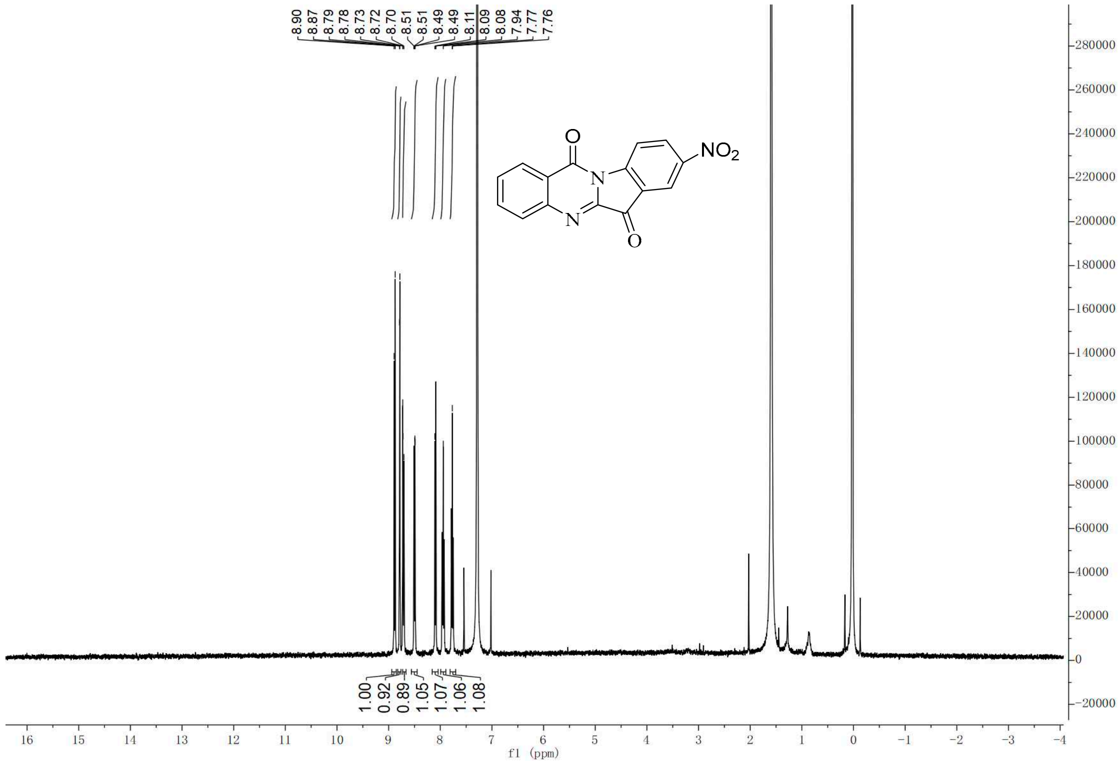

Figure S51. ^{1}H NMR Spectrum (DMSO-d_6, 400 MHz) of **B7**.

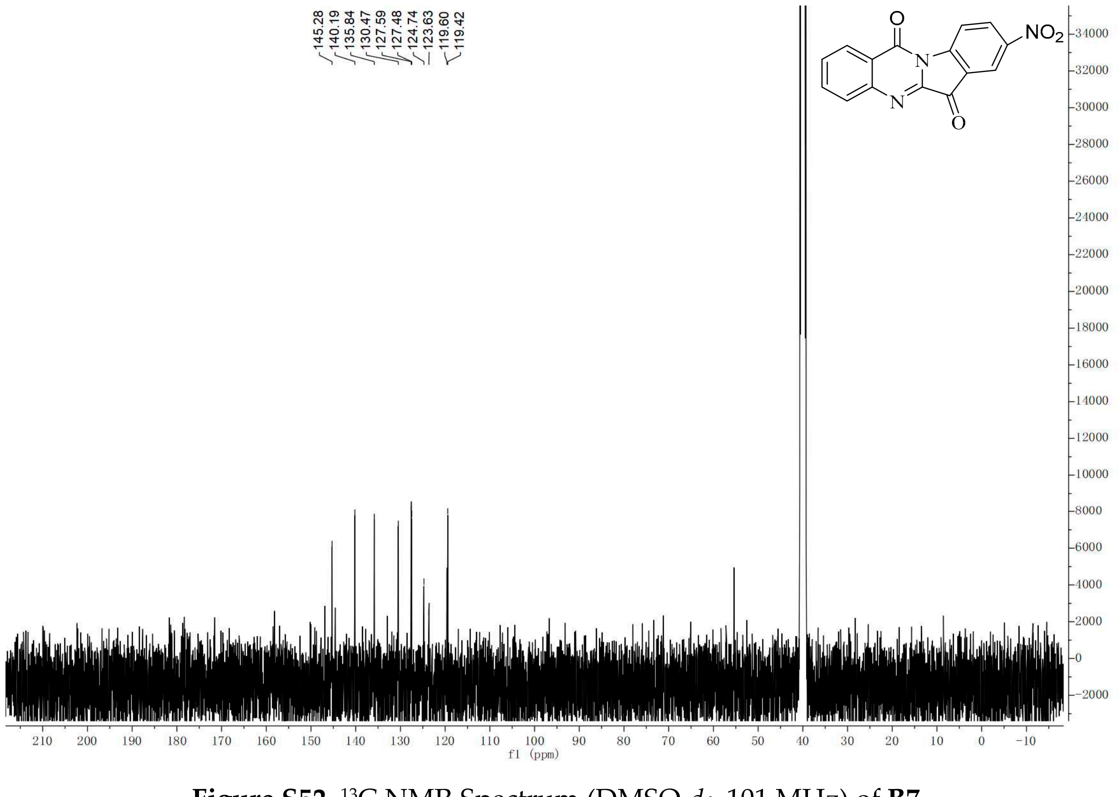

Figure S52. ^{13}C NMR Spectrum (DMSO-d_6, 101 MHz) of **B7**.

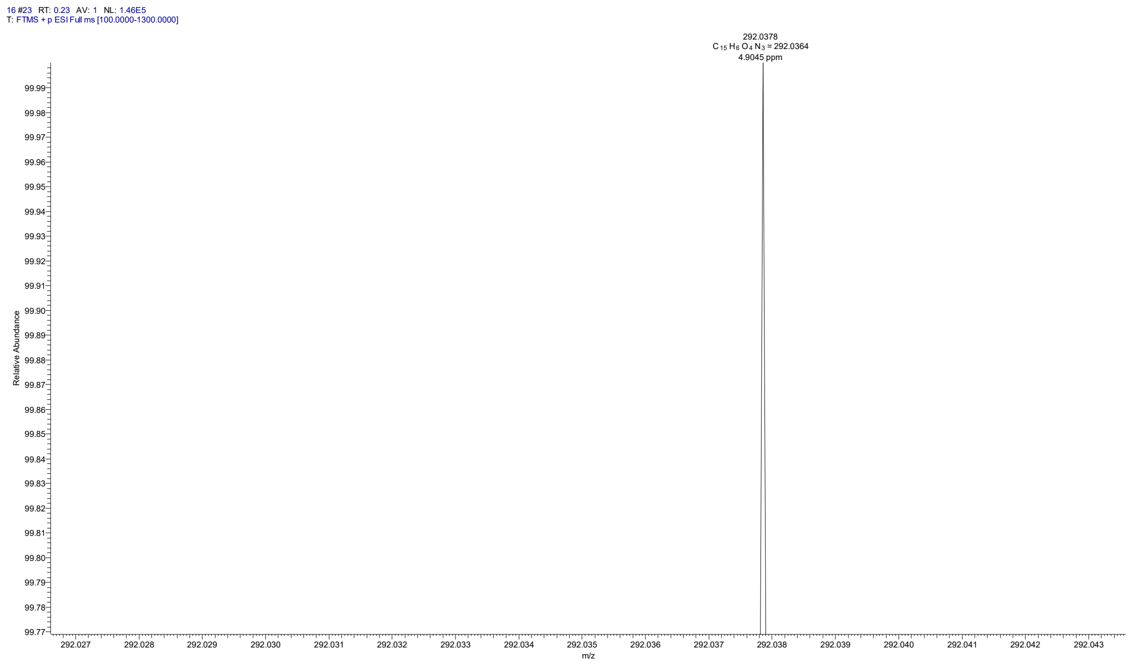

Figure S53. **B7** HRMS for $C_{15}H_7O_4N_3$ $[M-H]^-$. Calcd. 292.0364. Found 292.0378.

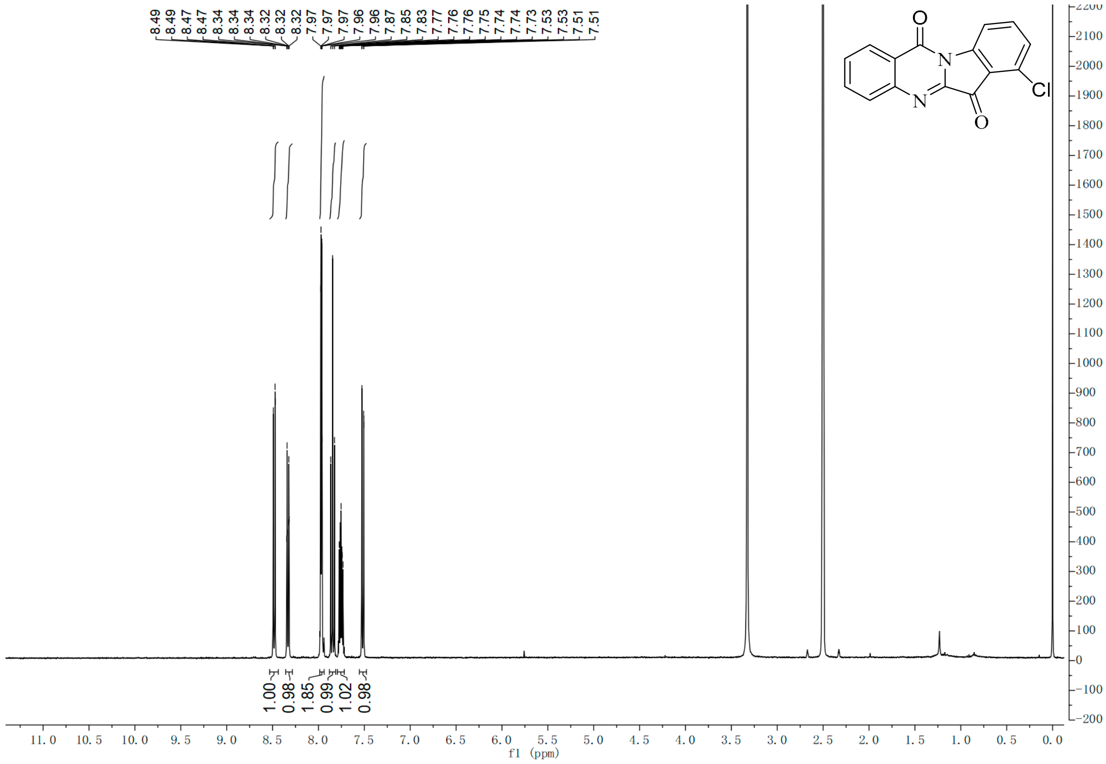

Figure S54. ^1H NMR Spectrum (DMSO-d_6, 400 MHz) of **B8**.

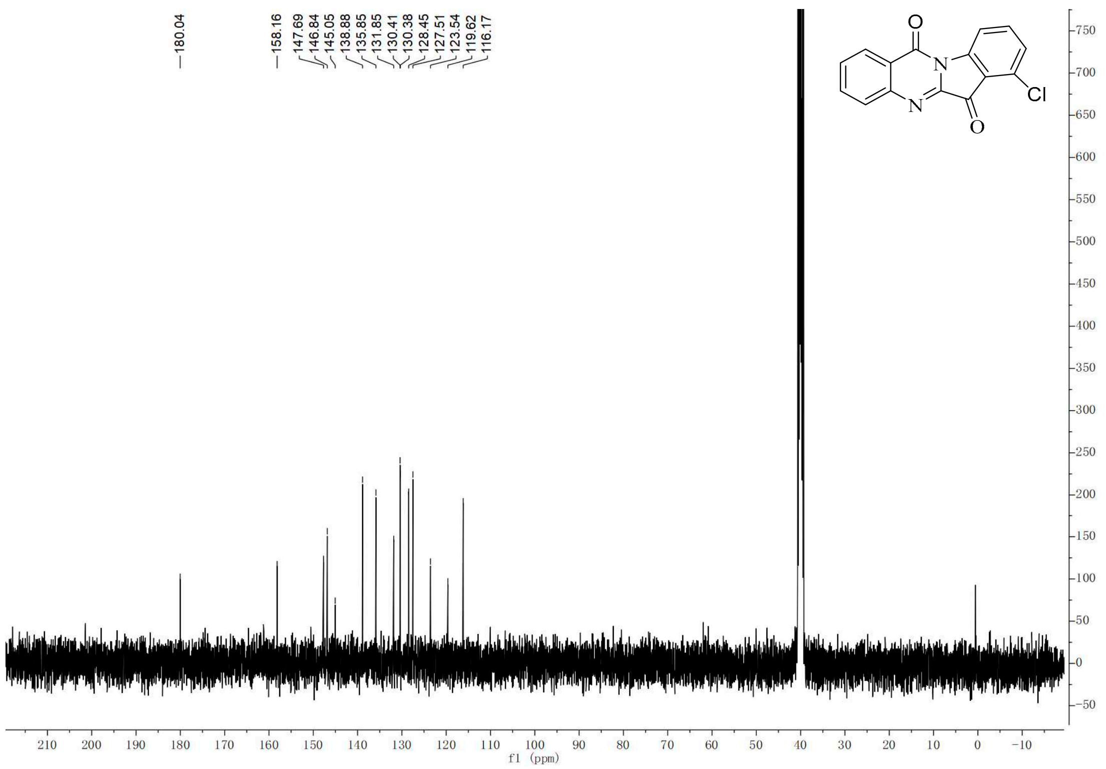

Figure S55. ^{13}C NMR Spectrum (DMSO-d_6, 101 MHz) of **B8**.

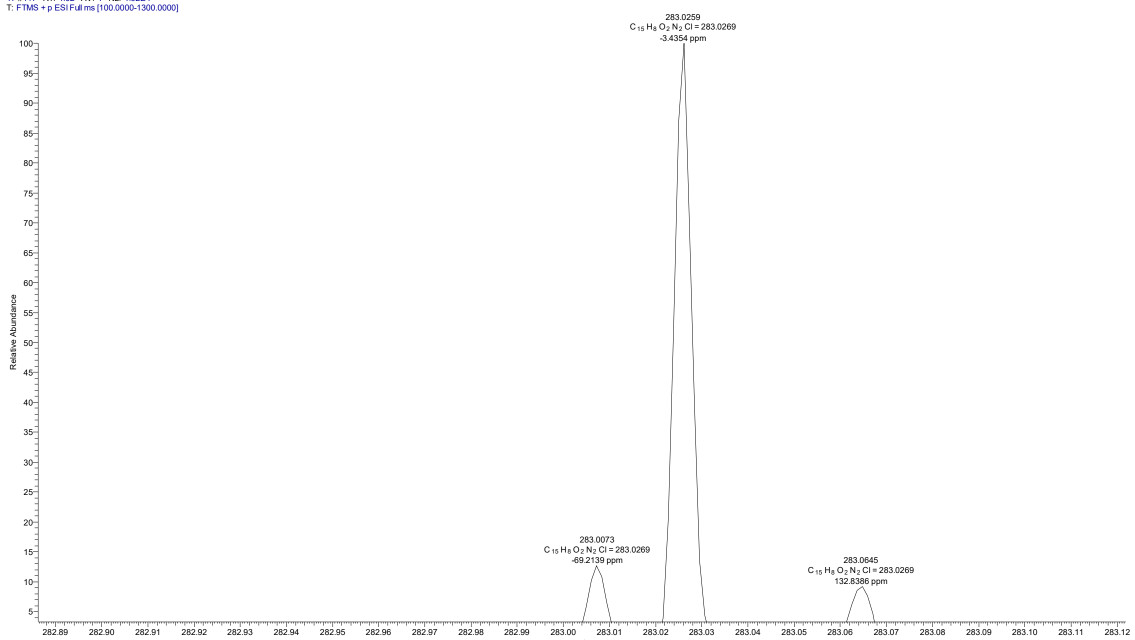

Figure S56. **B8** HRMS for $C_{15}H_7O_2N_2Cl$ $[M+H]^+$. Calcd. 283.0269. Found 283.0259.

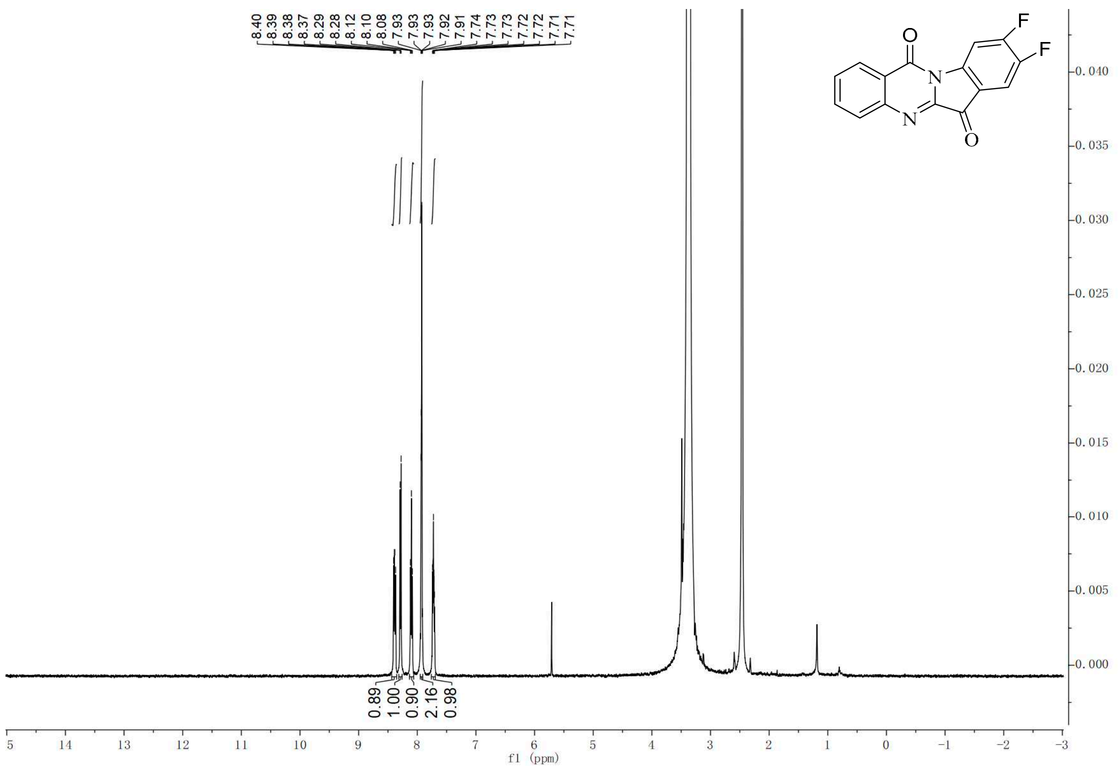

Figure S57. ^{1}H NMR Spectrum (DMSO-d_6, 400 MHz) of **B9**.

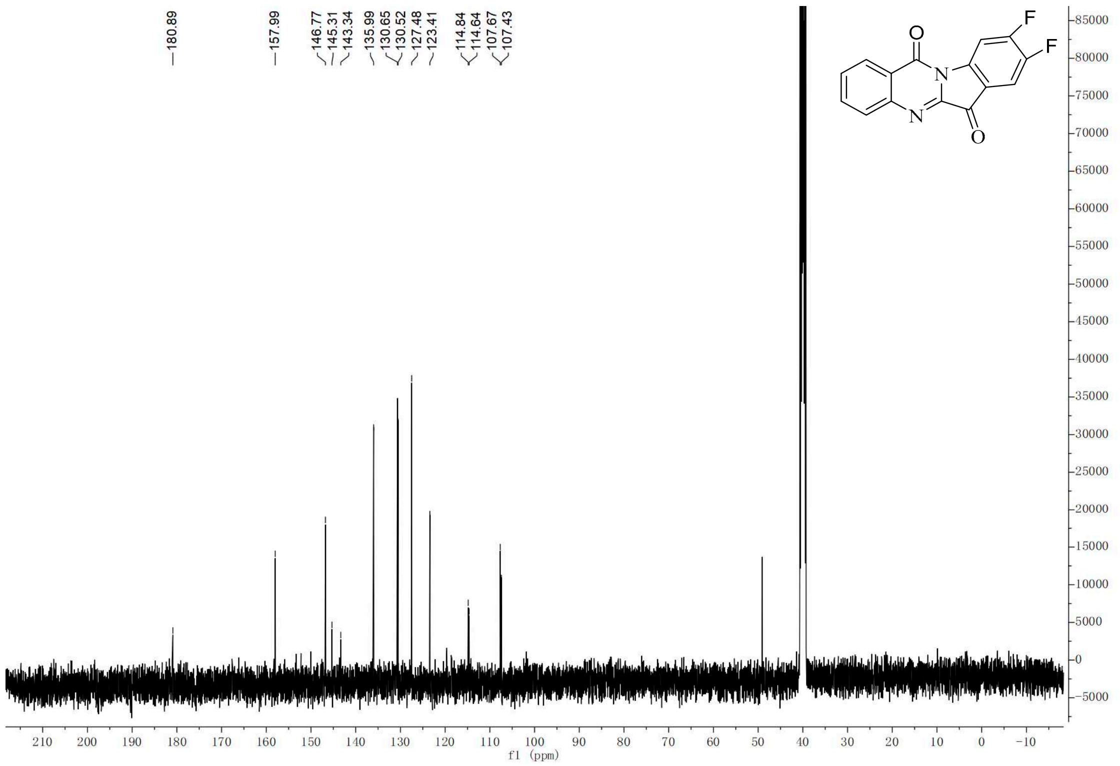

Figure S58. ^{13}C NMR Spectrum (DMSO-d_6, 101 MHz) of **B9**.

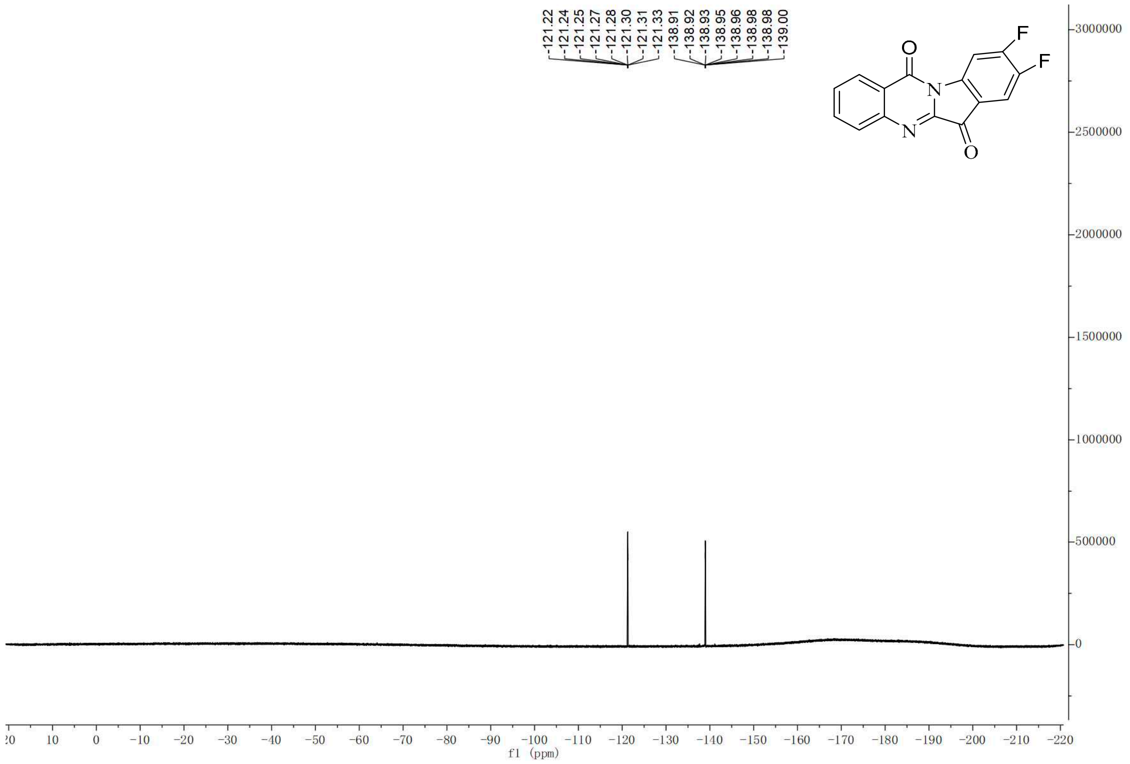

Figure S59. ¹⁹F NMR Spectrum (DMSO-d_6, 376 MHz) of **B9**.

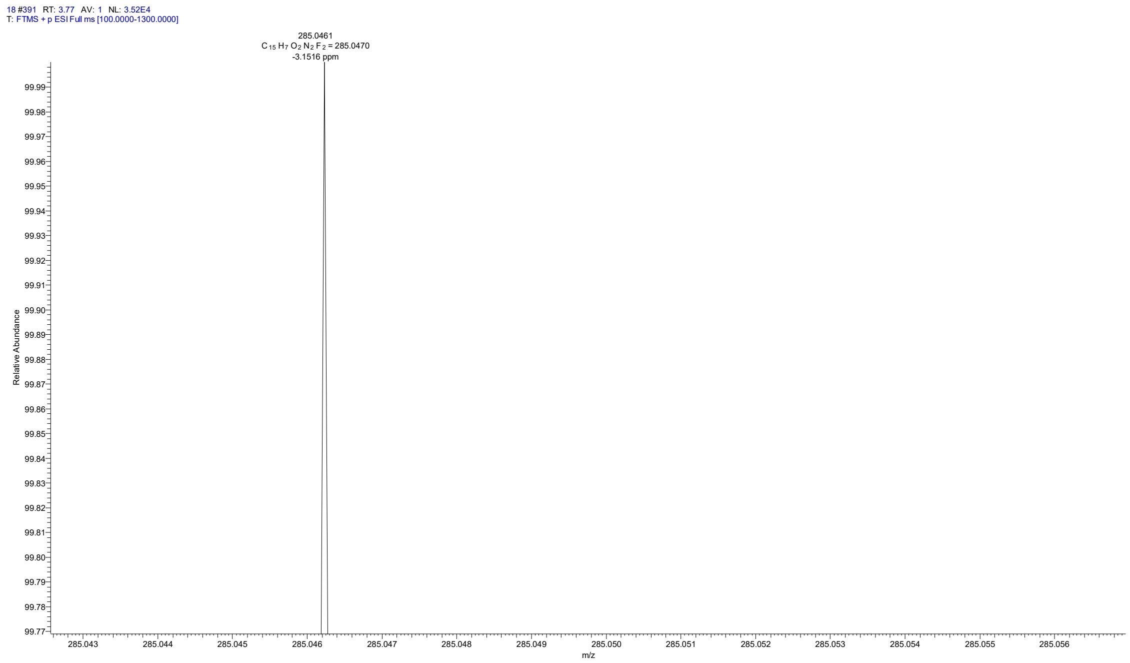

Figure S60. **B9** HRMS for $C_{15}H_6O_2N_2F_2$ $[M+H]^+$. Calcd. 285.0470. Found 285.0461.

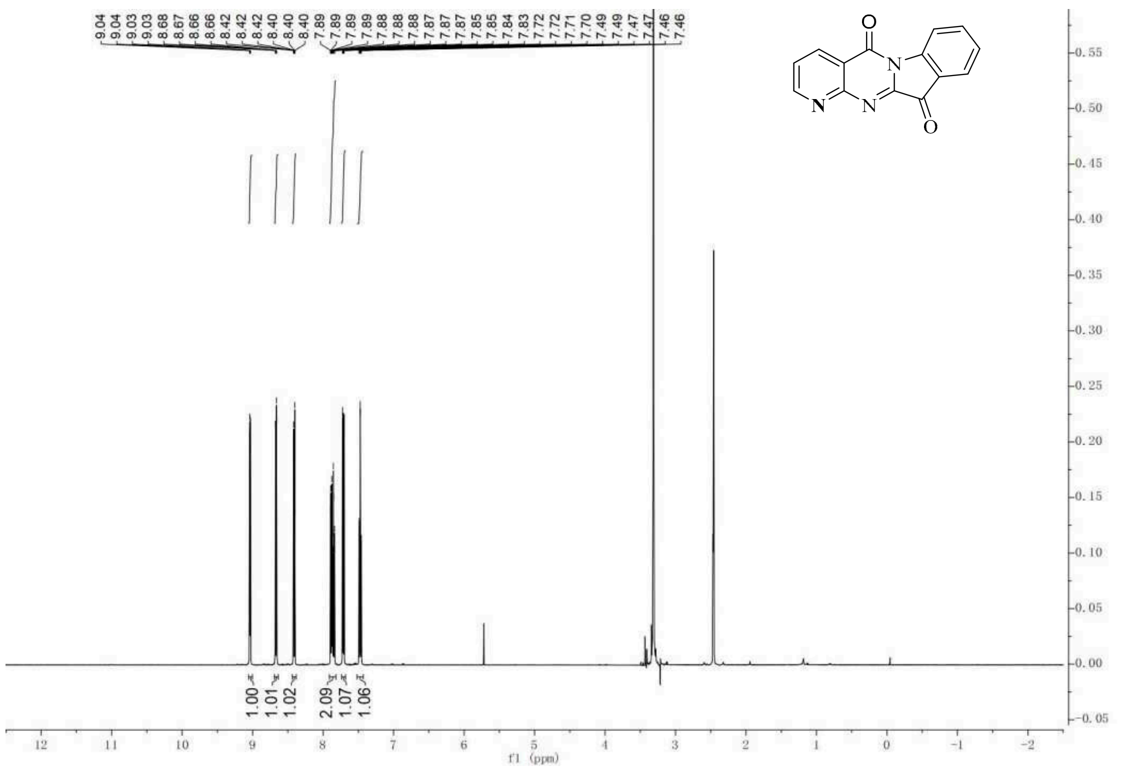

Figure S61. ^{1}H NMR Spectrum (DMSO-d_6, 500 MHz) of **C1**.

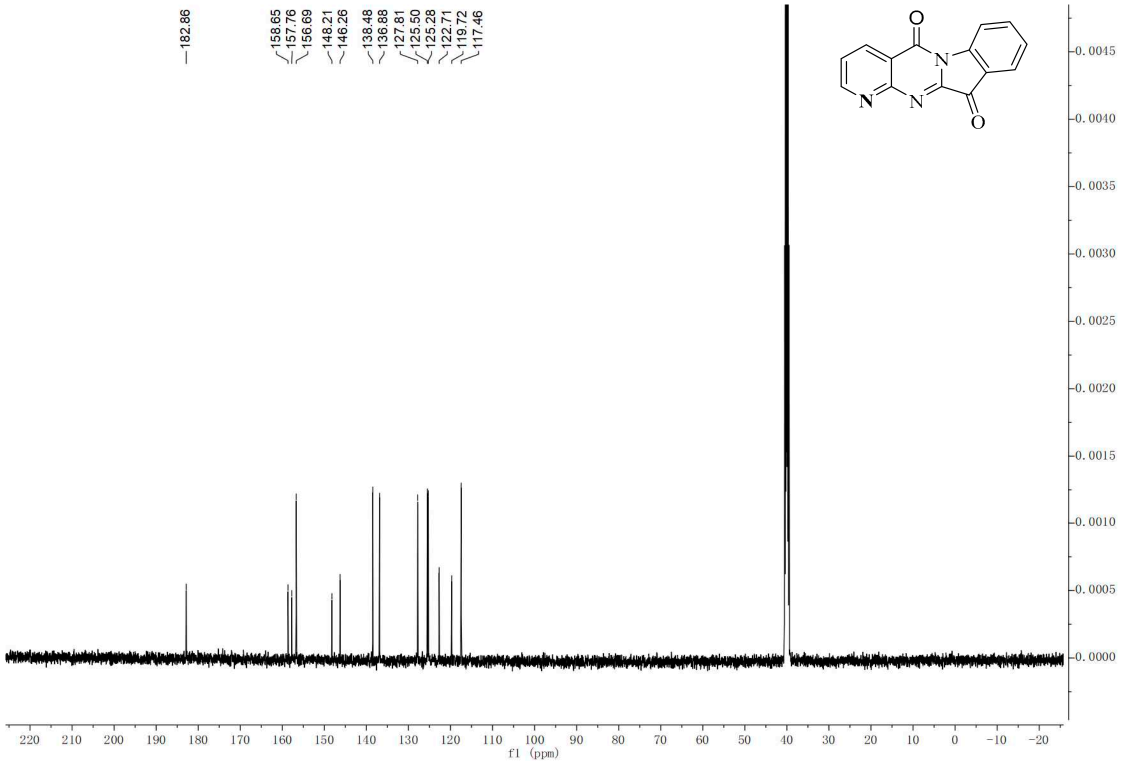

Figure S62. ^{13}C NMR Spectrum (DMSO-d_6, 101 MHz) of **C1**.

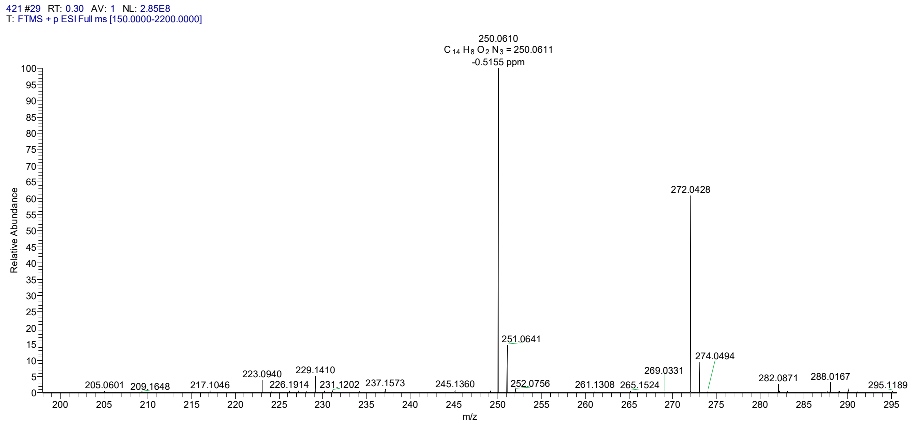

Figure S63. **C1** HRMS for $C_{14}H_{7}O_{2}N_{3}$ $[M+H]^{+}$. Calcd. 250.0611. Found 250.0610.

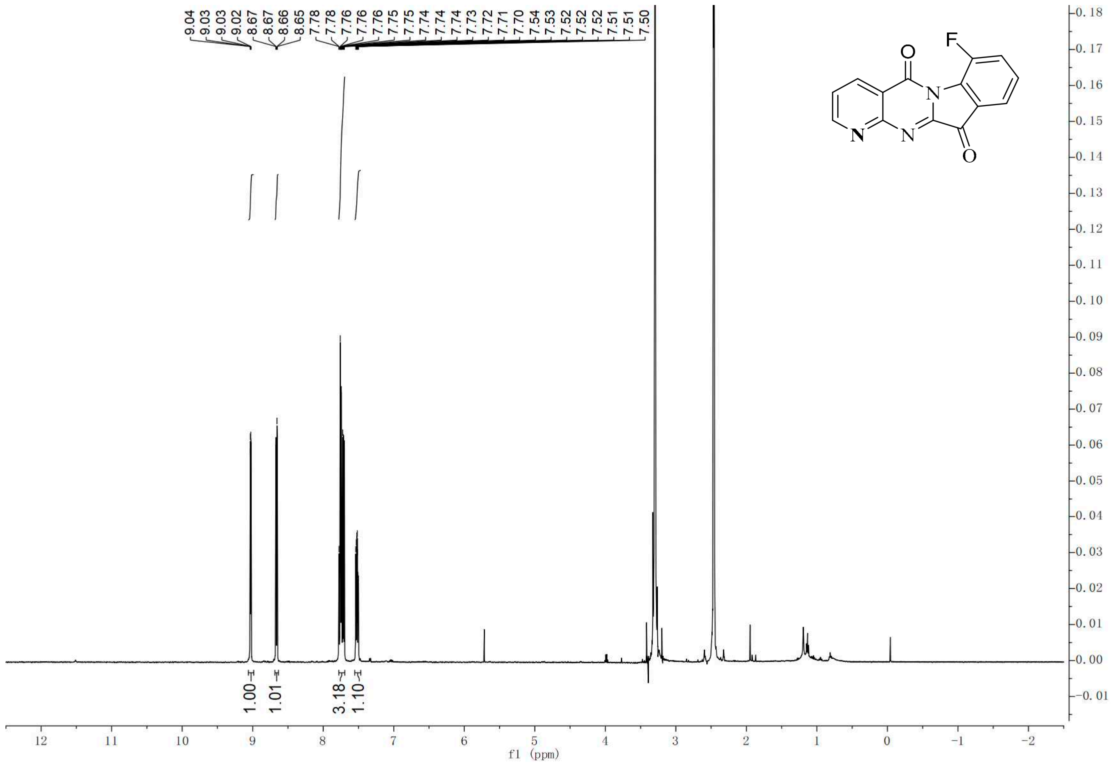

Figure S64. ^{1}H NMR Spectrum (DMSO-d_6, 400 MHz) of **C2**.

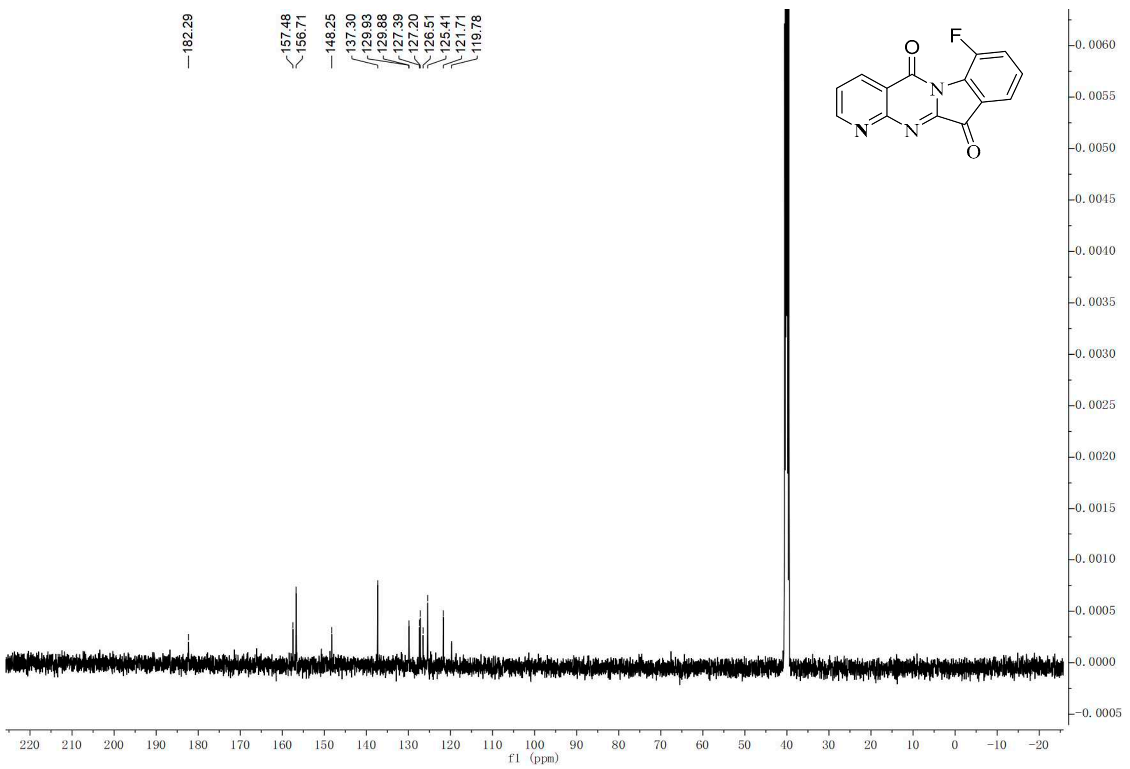

Figure S65. ^{13}C NMR Spectrum (DMSO-d_6, 101 MHz) of **C2**.

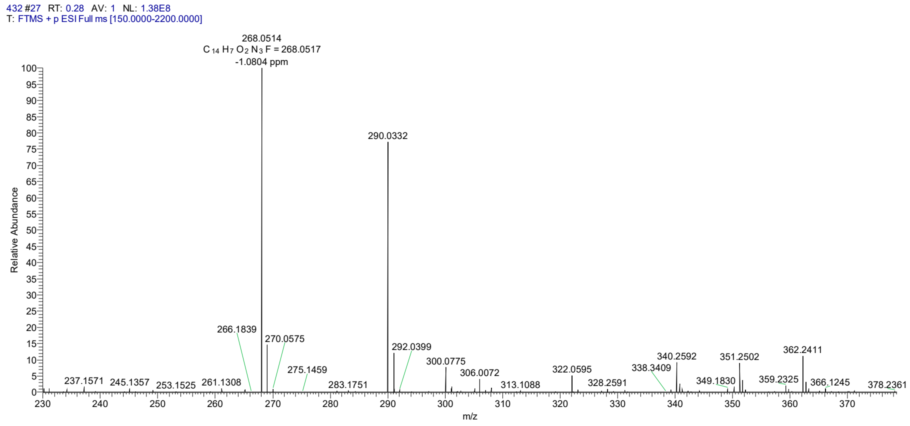

Figure S66. **C2** HRMS for $C_{14}H_6O_2N_3F$ $[M+H]^+$. Calcd.268.0517. Found 268.0514.

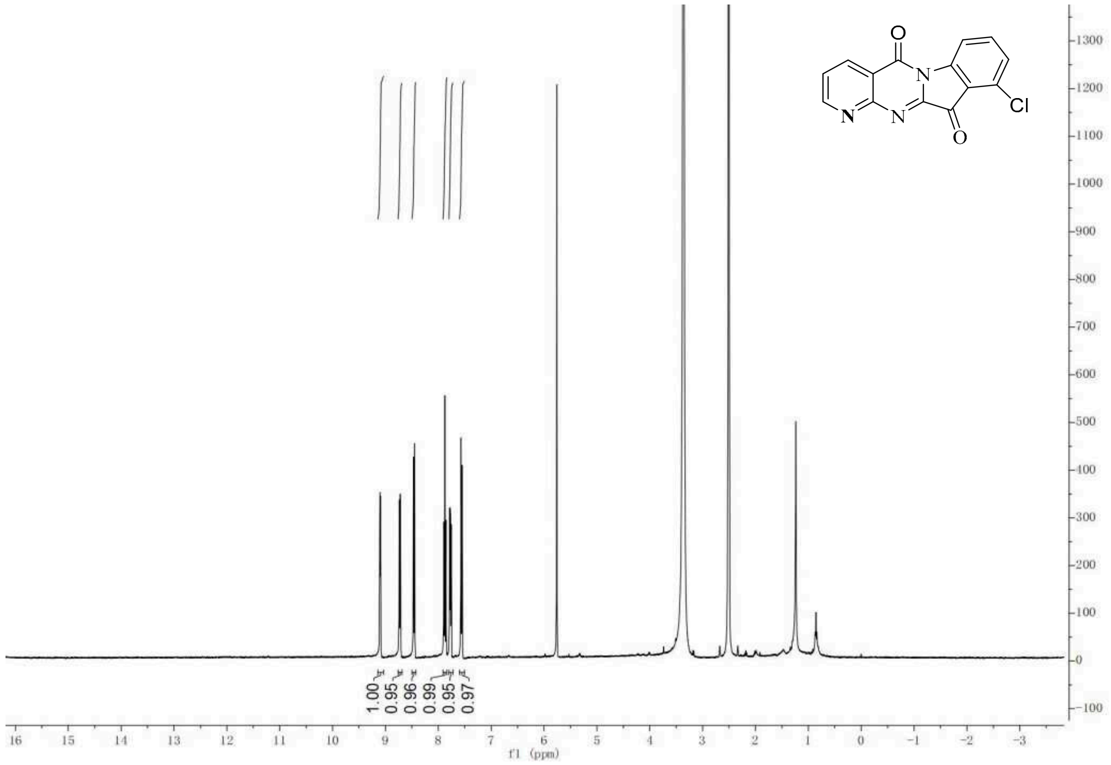

Figure S67. ^{1}H NMR Spectrum (DMSO-d_6, 400 MHz) of **C3**.

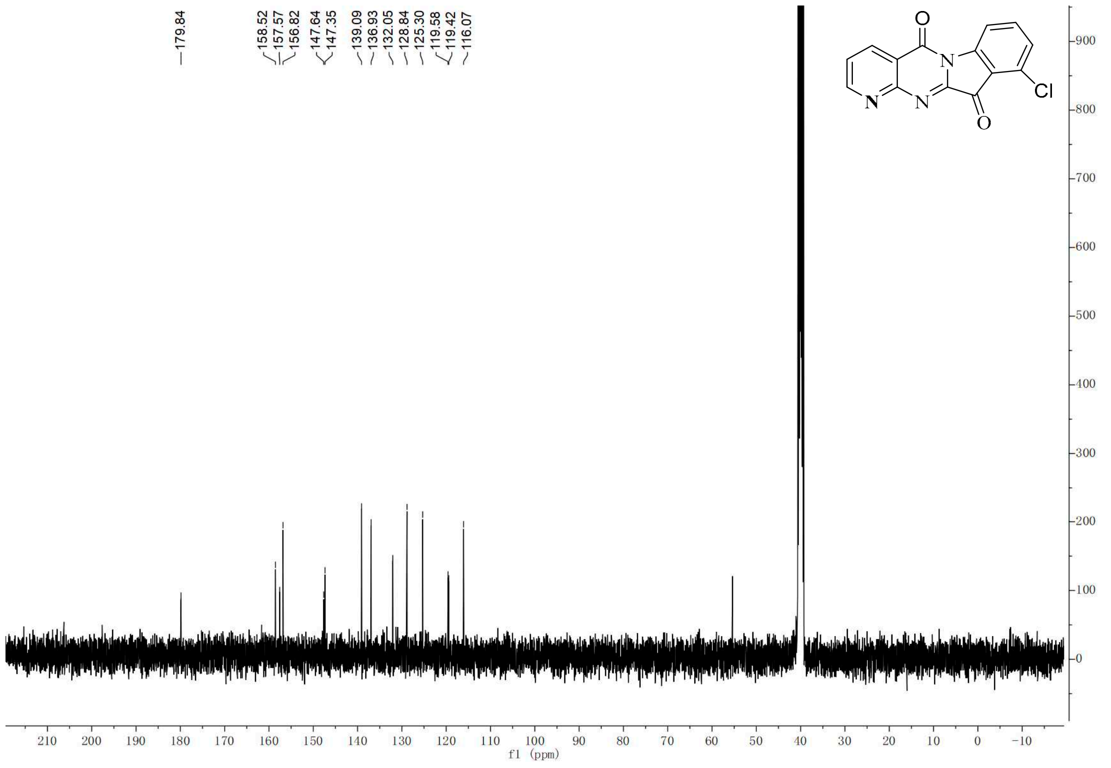

Figure S68. ^{13}C NMR Spectrum (DMSO-d_6, 101 MHz) of **C3**.

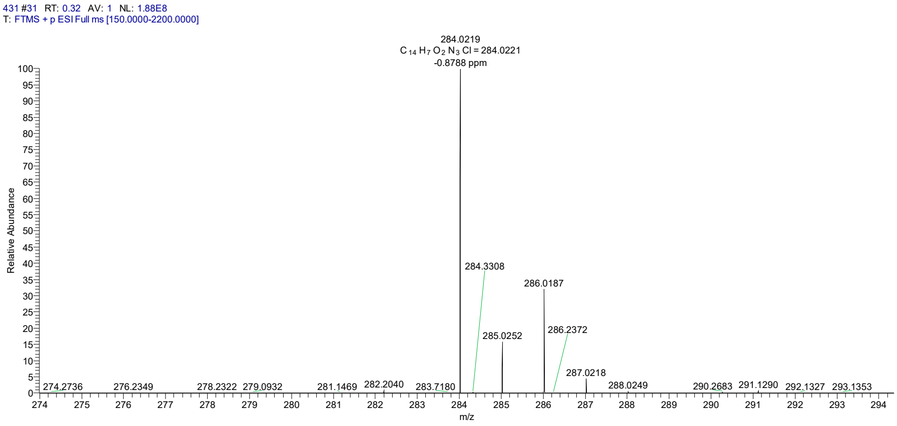

Figure S69. **C3** HRMS for $C_{14}H_6O_2N_3Cl$ $[M+H]^+$. Calcd.284.0221. Found 284.0219.

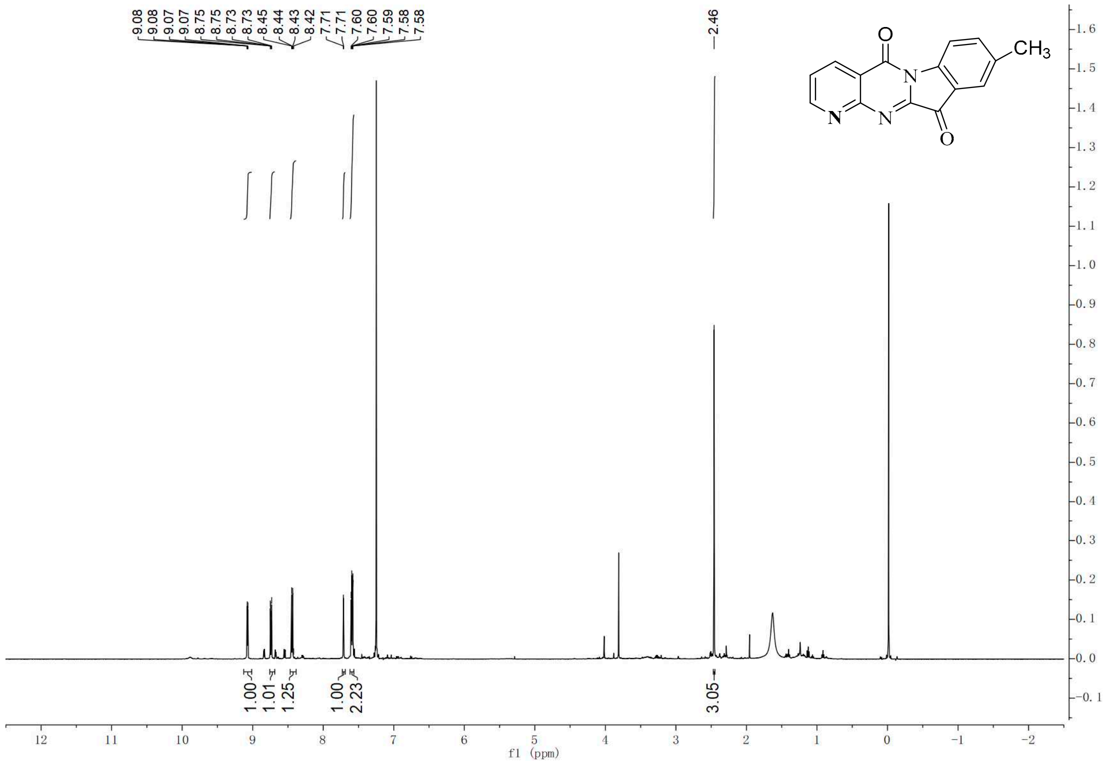

Figure S70. ^{1}H NMR Spectrum (Chloroform-*d*, 400 MHz) of **C4**.

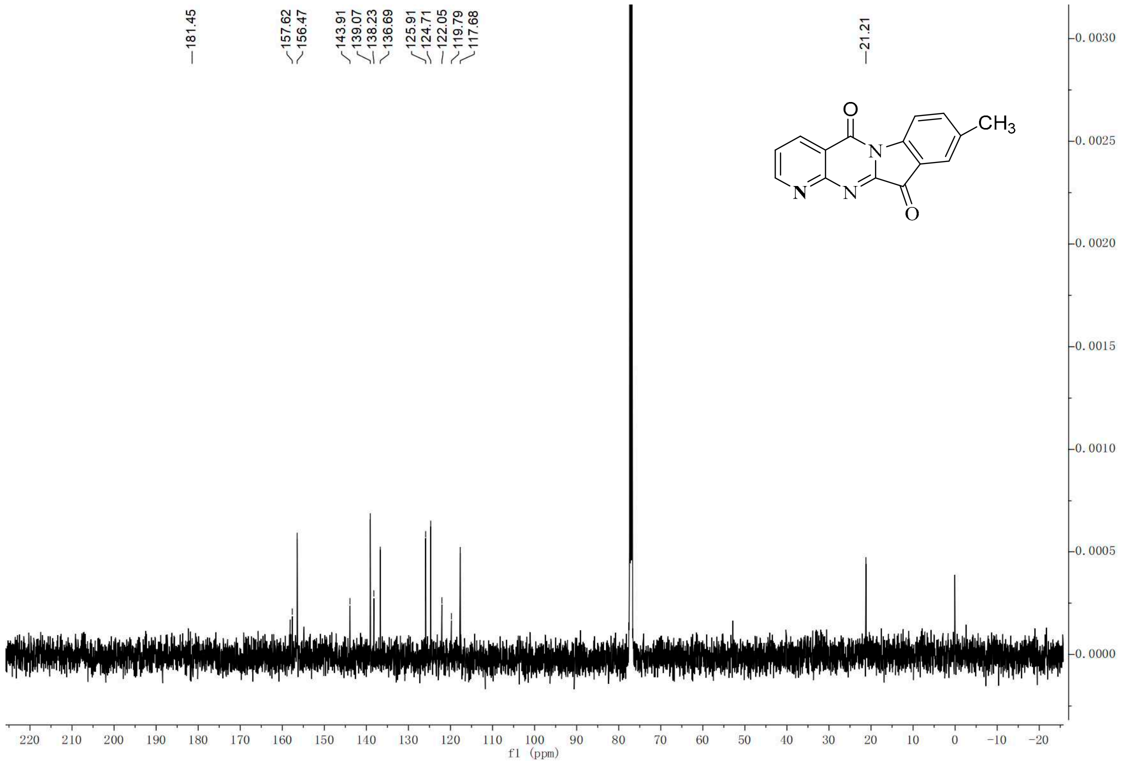

Figure S71. ^{13}C NMR Spectrum (Chloroform-*d*, 101 MHz) of **C4**.

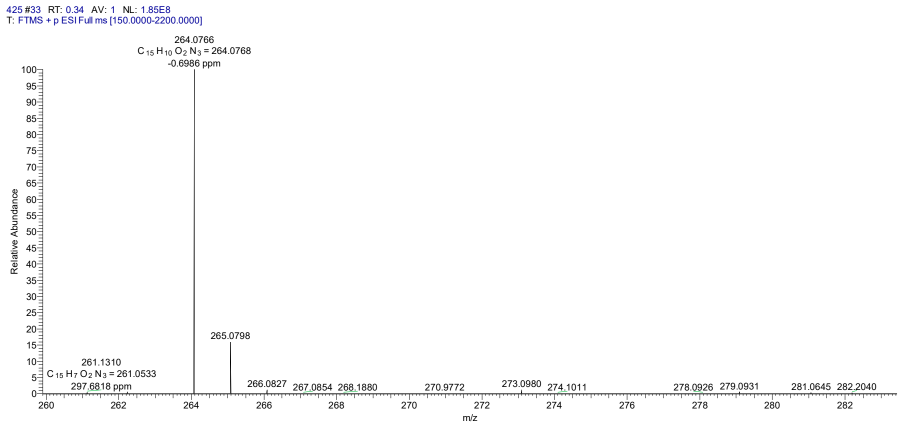

Figure S72. **C4** HRMS for $C_{15}H_9N_3O_2$ $[M+H]^+$. Calcd.264.0768. Found 264.0766

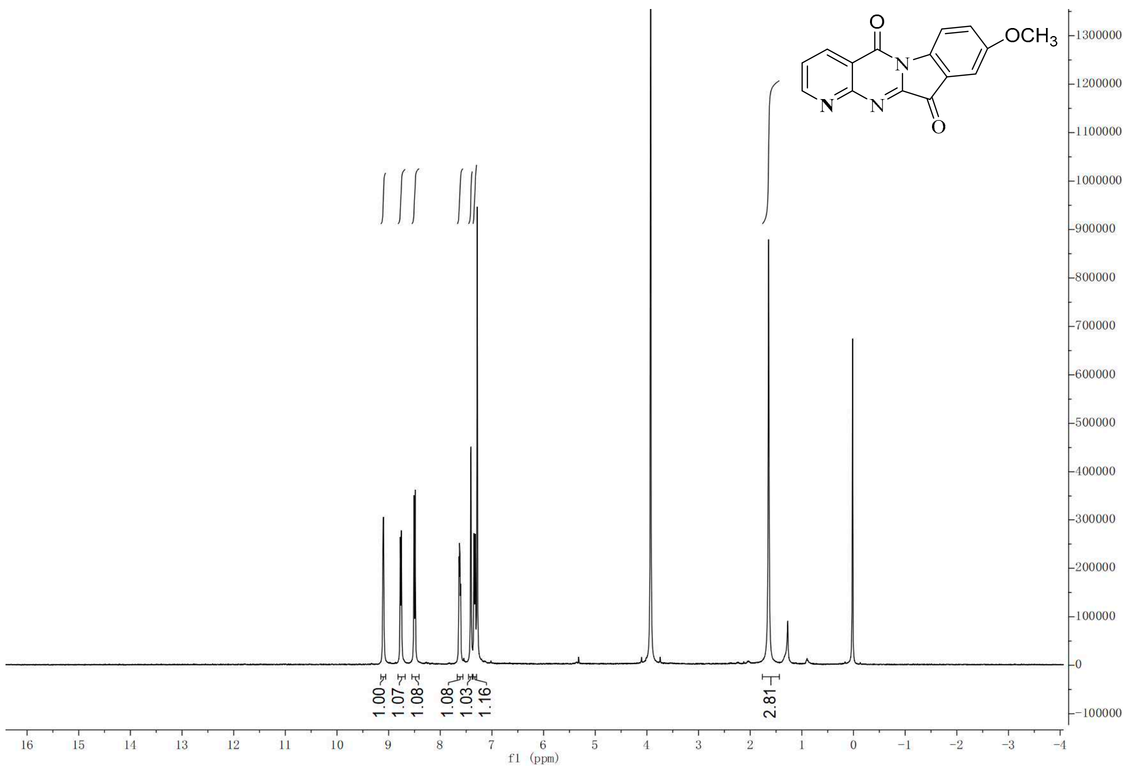

Figure S73. ^{1}H NMR Spectrum (DMSO-d_6, 400 MHz) of **C5**.

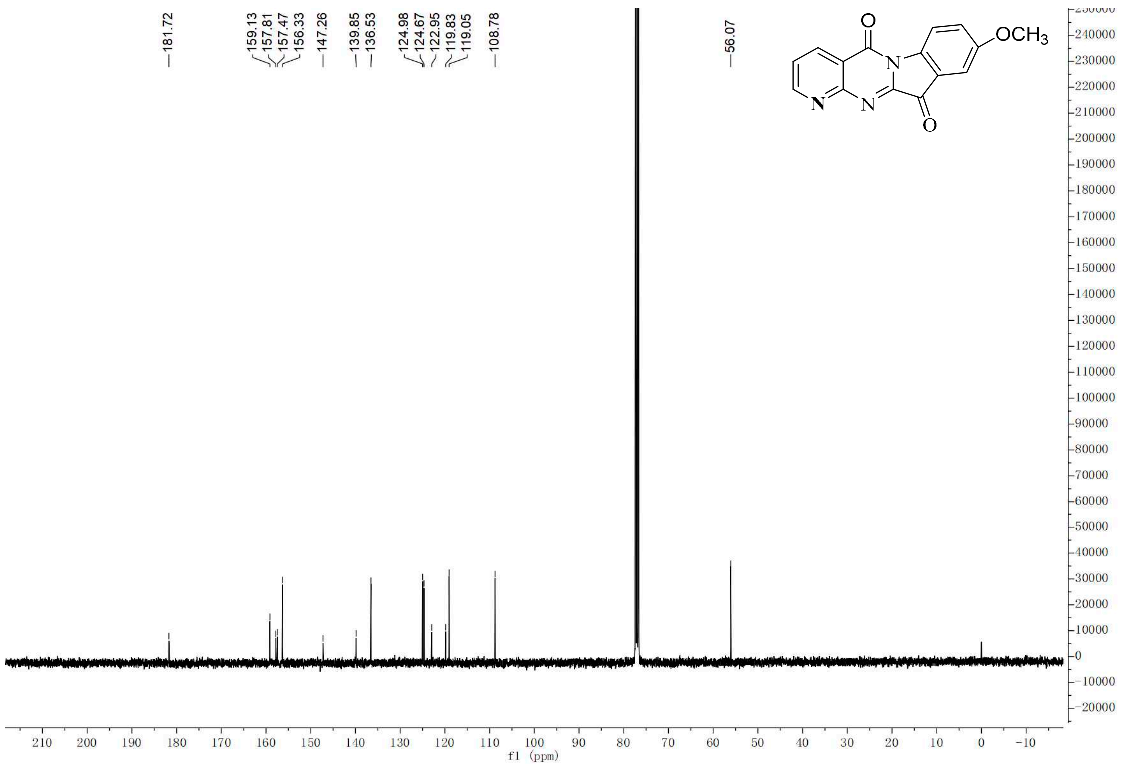

Figure S74. ^{13}C NMR Spectrum (Chloroform-*d*, 101 MHz) of **C5**.

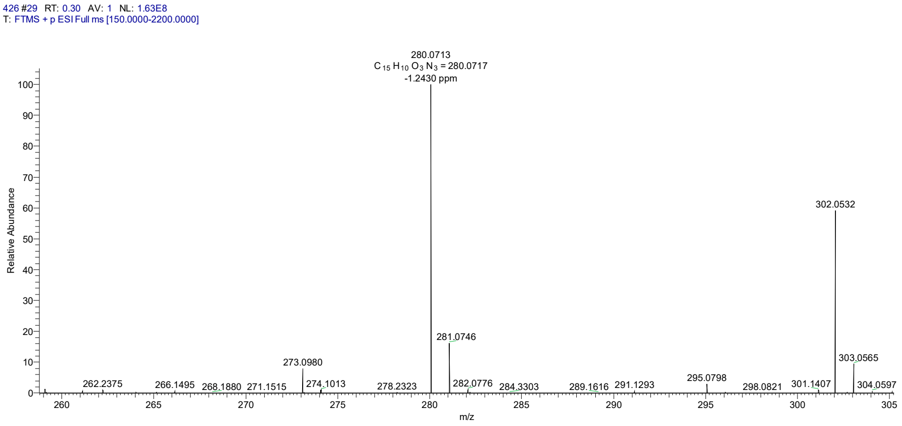

Figure S75. **C5** HRMS for $C_{15}H_9N_3O_3$ $[M+H]^+$. Calcd.280.0717. Found 280.0713.

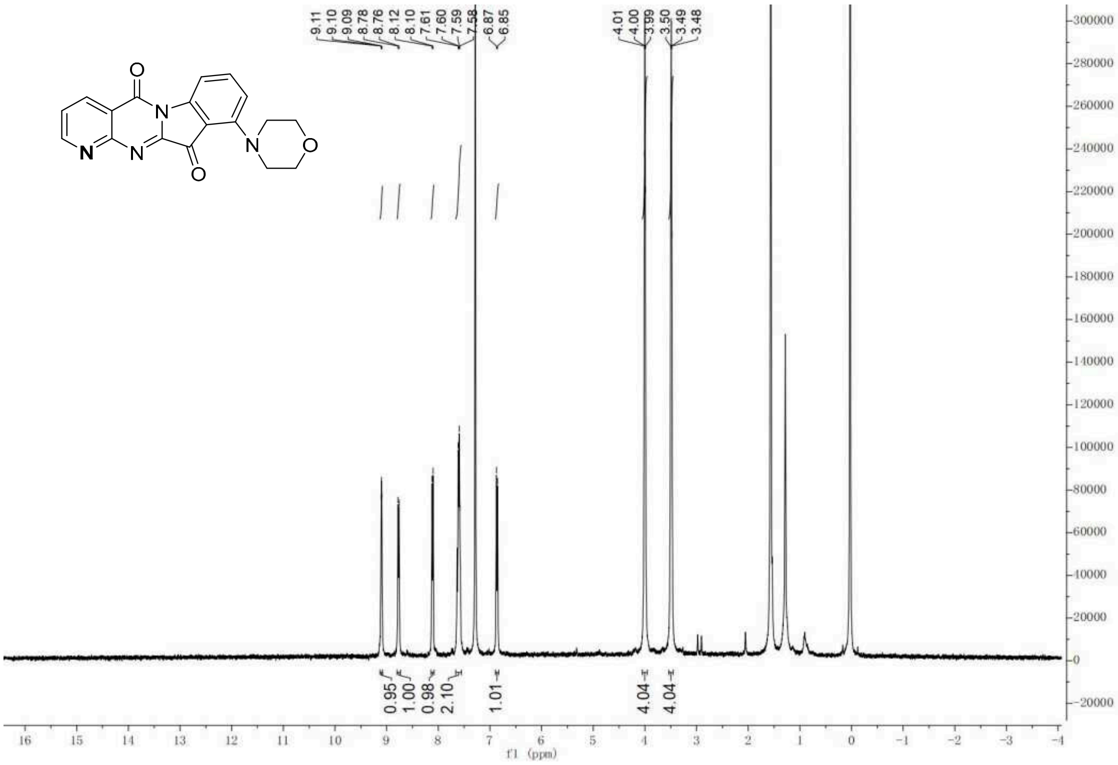

Figure S76. ^{1}H NMR Spectrum (DMSO-d6, 400 MHz) of **D1**.

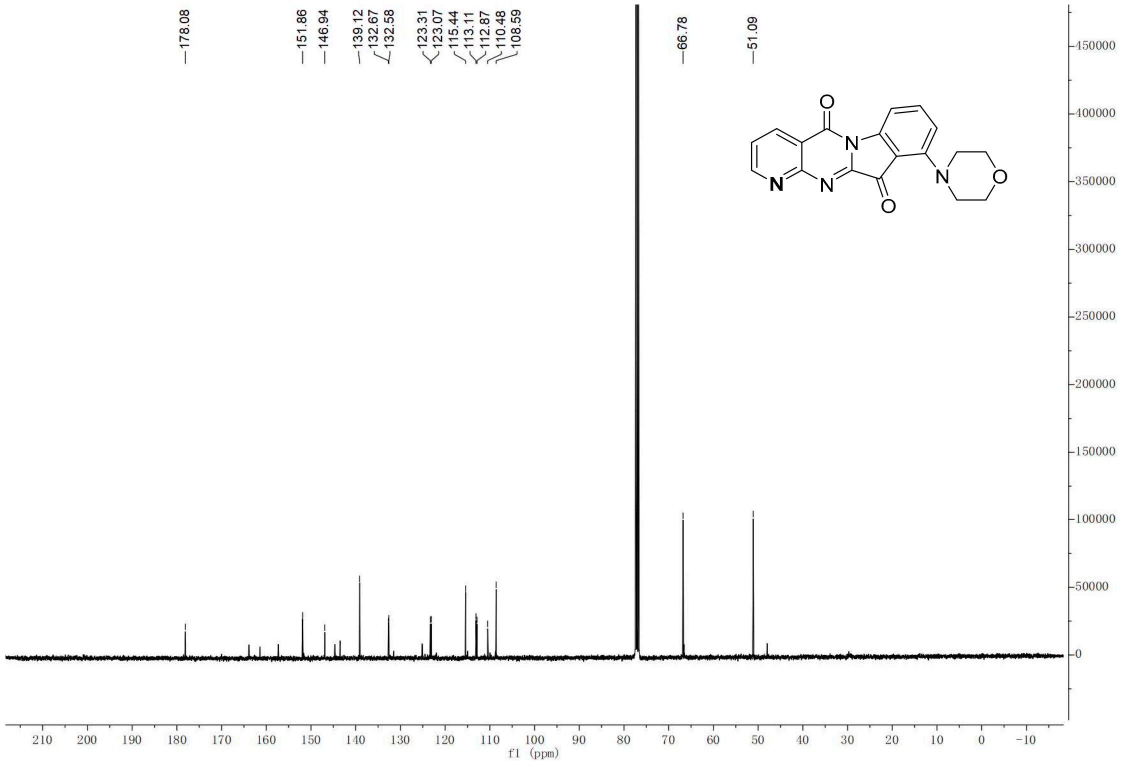

Figure S77. ^{13}C NMR Spectrum (DMSO-d6, 101 MHz) of **D1**.

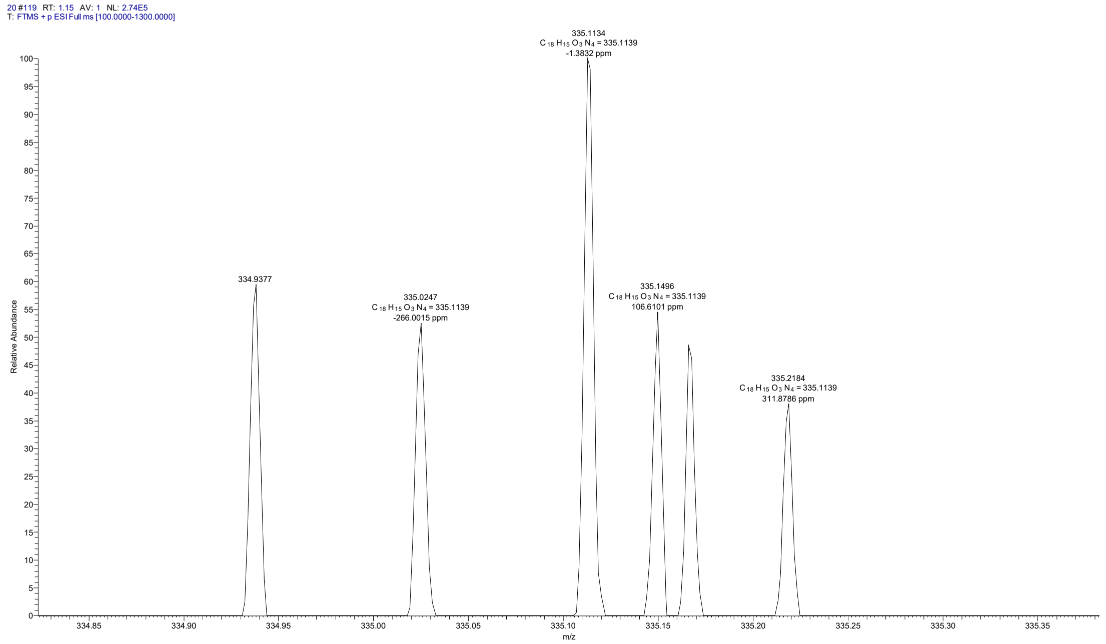

Figure S78. **D1** HRMS for $C_{18}H_{15}N_4O_3$[M+H]$^+$. Calcd. 335.1139. Found 335.1134.

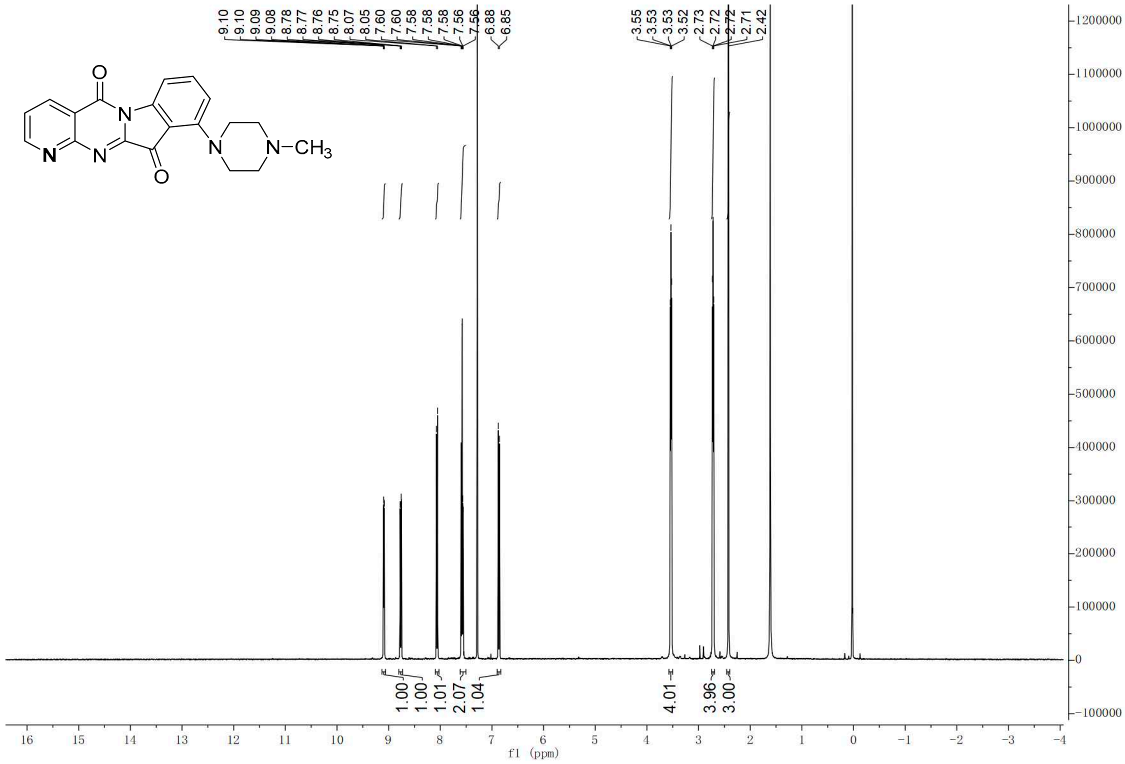

Figure S79. ^{1}H NMR Spectrum (Chloroform-*d*, 400 MHz) of **D2**.

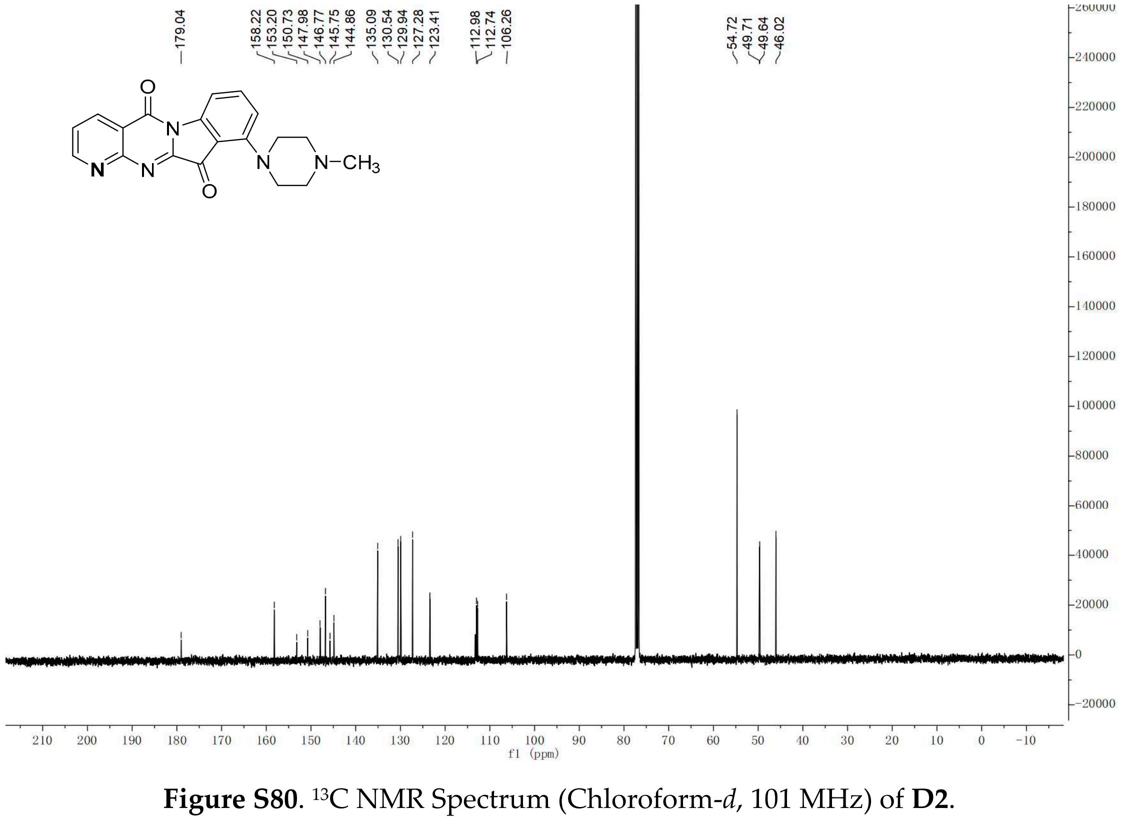

Figure S80. ^{13}C NMR Spectrum (Chloroform-*d*, 101 MHz) of **D2**.

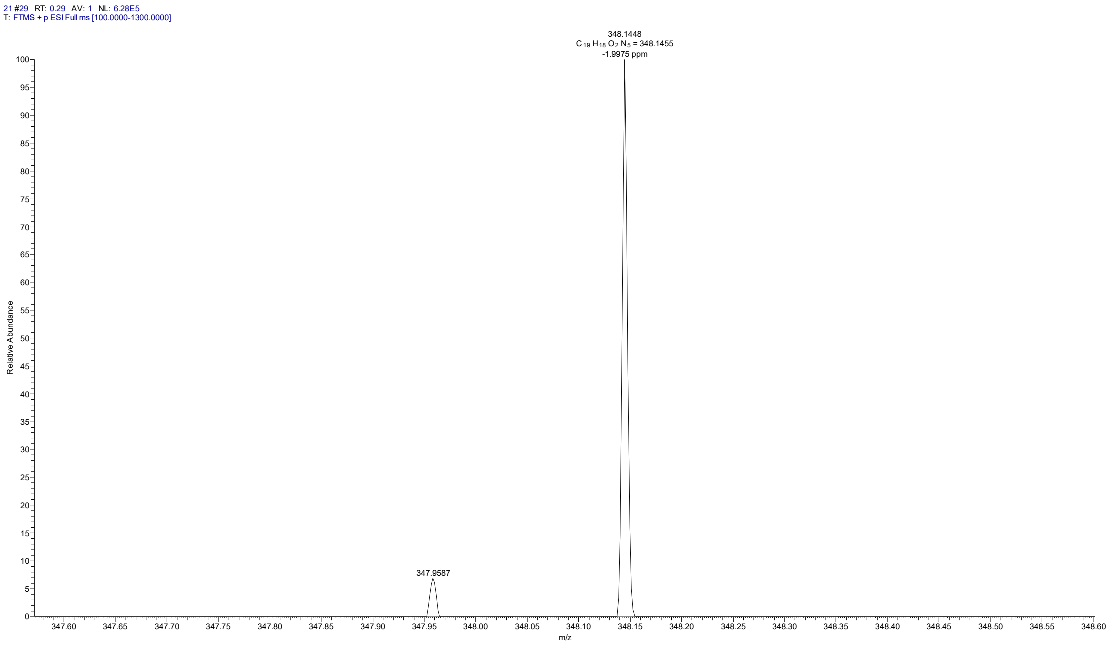

Figure S81. **D2** HRMS for $C_{19}H_{18}N_5O_2[M+H]^+$. Calcd. 348.1455. Found 348.1448.

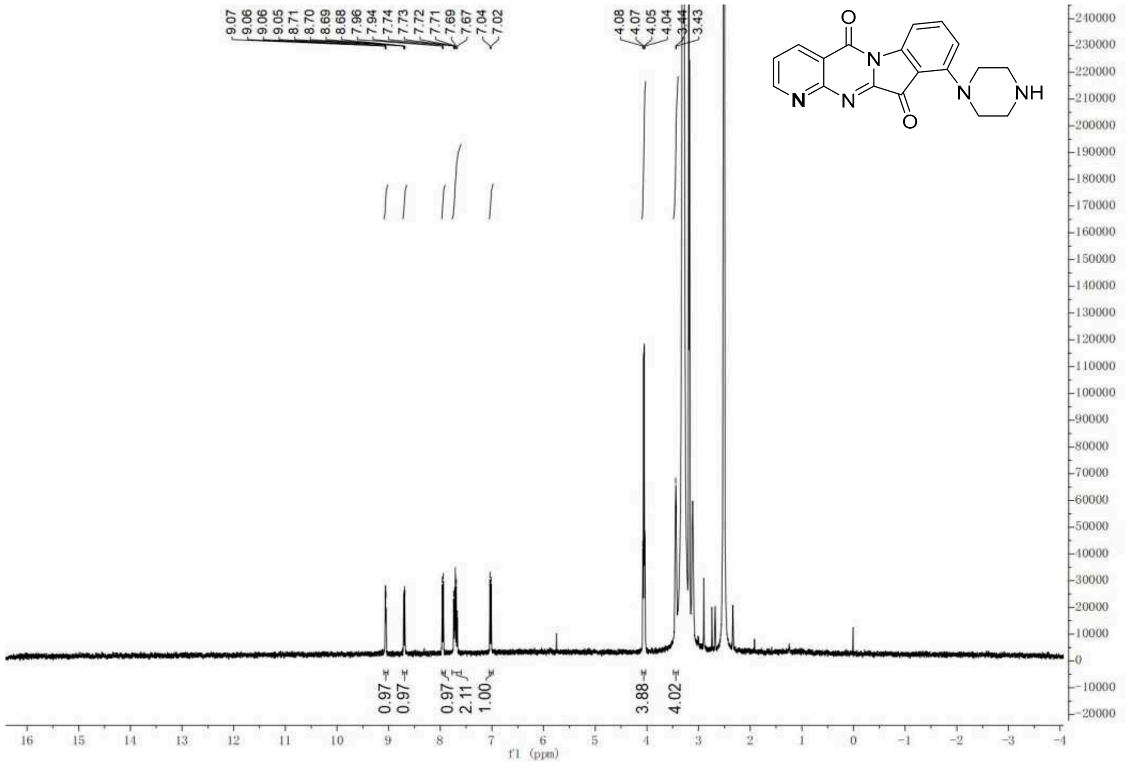

Figure S82. ^{1}H NMR Spectrum (DMSO-d6, 400 MHz) of **D3**.

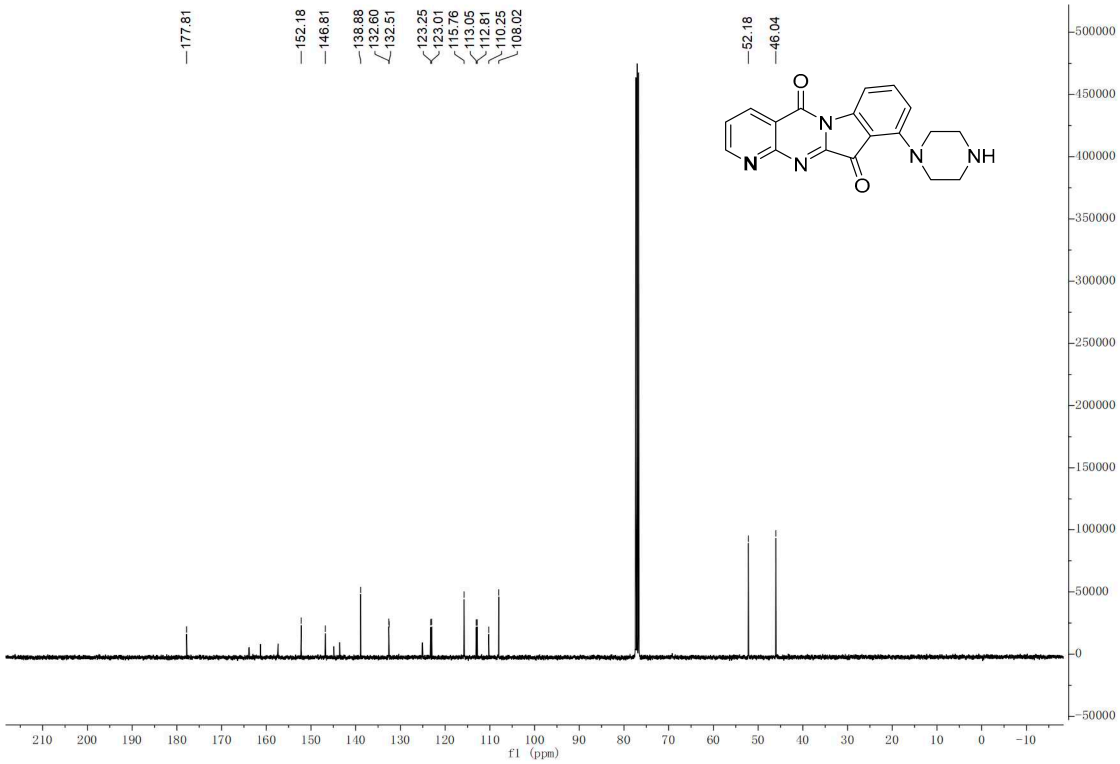

Figure S83. ${}^{13}C$ NMR Spectrum (Chloroform-*d*, 101 MHz) of **D3**.

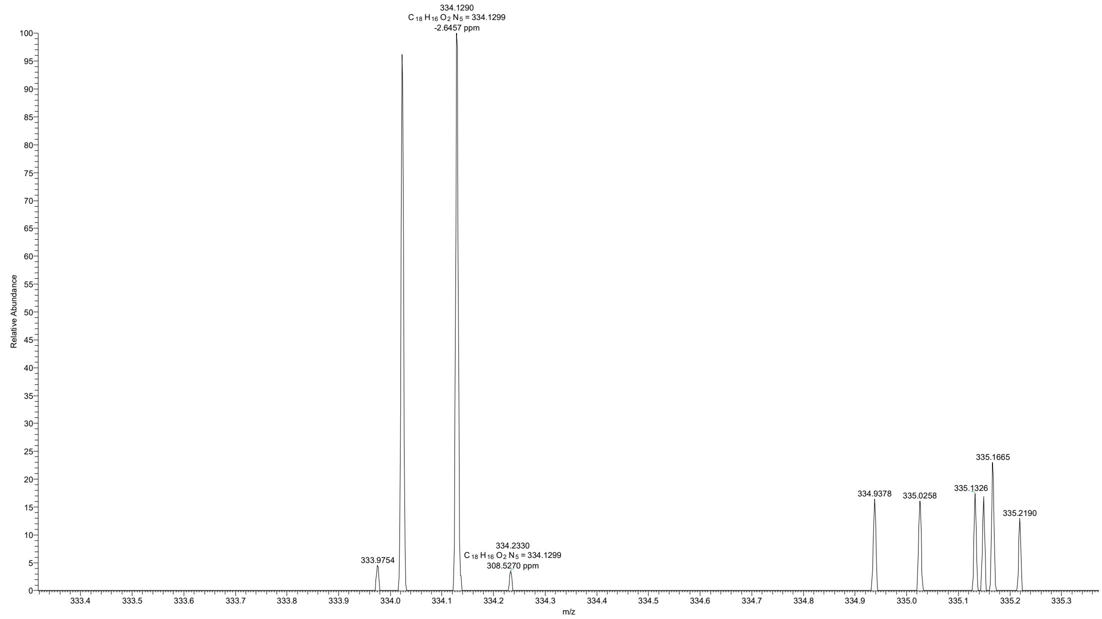

Figure S84. **D3** HRMS for $C_{18}H_{15}N_5O_2[M+H]^+$. Calcd. 334.1299. Found 334.1290.

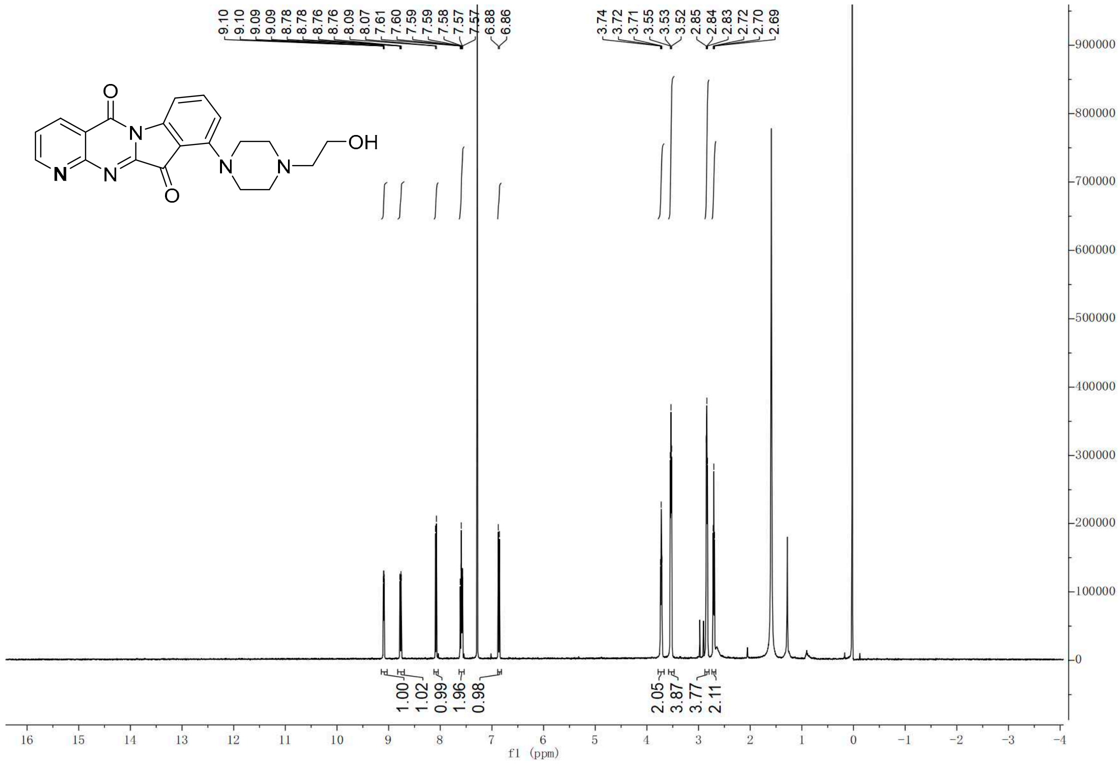

Figure S85. ^{1}H NMR Spectrum (Chloroform-*d*, 400 MHz) of **D4**.

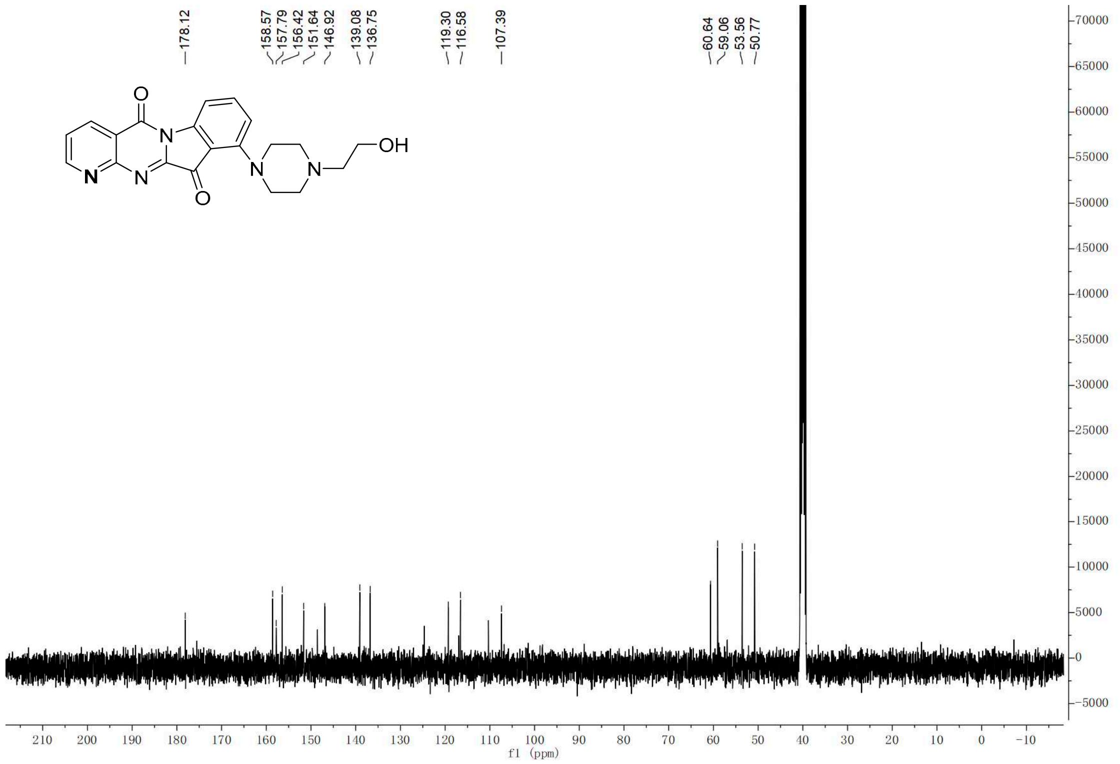

Figure S86. ^{13}C NMR Spectrum (DMSO-d6, 101 MHz) of **D4**.

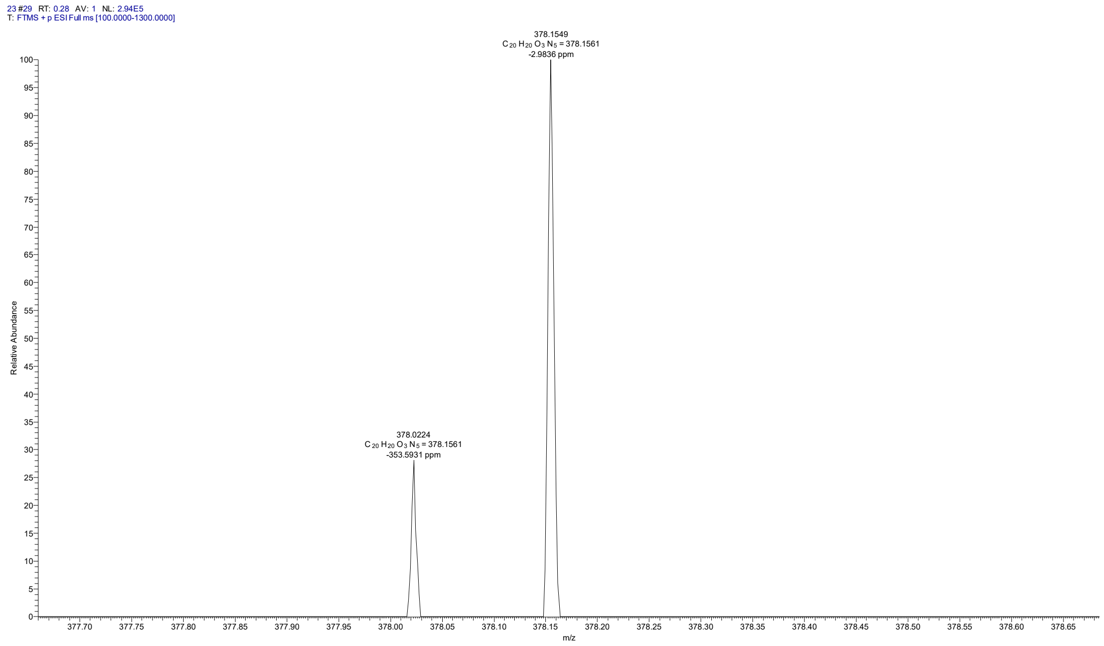

Figure S87. **D4** HRMS for $C_{20}H_{19}N_5O_3$ [M+H]$^+$. Calcd. 378.1561. Found 378.1549.

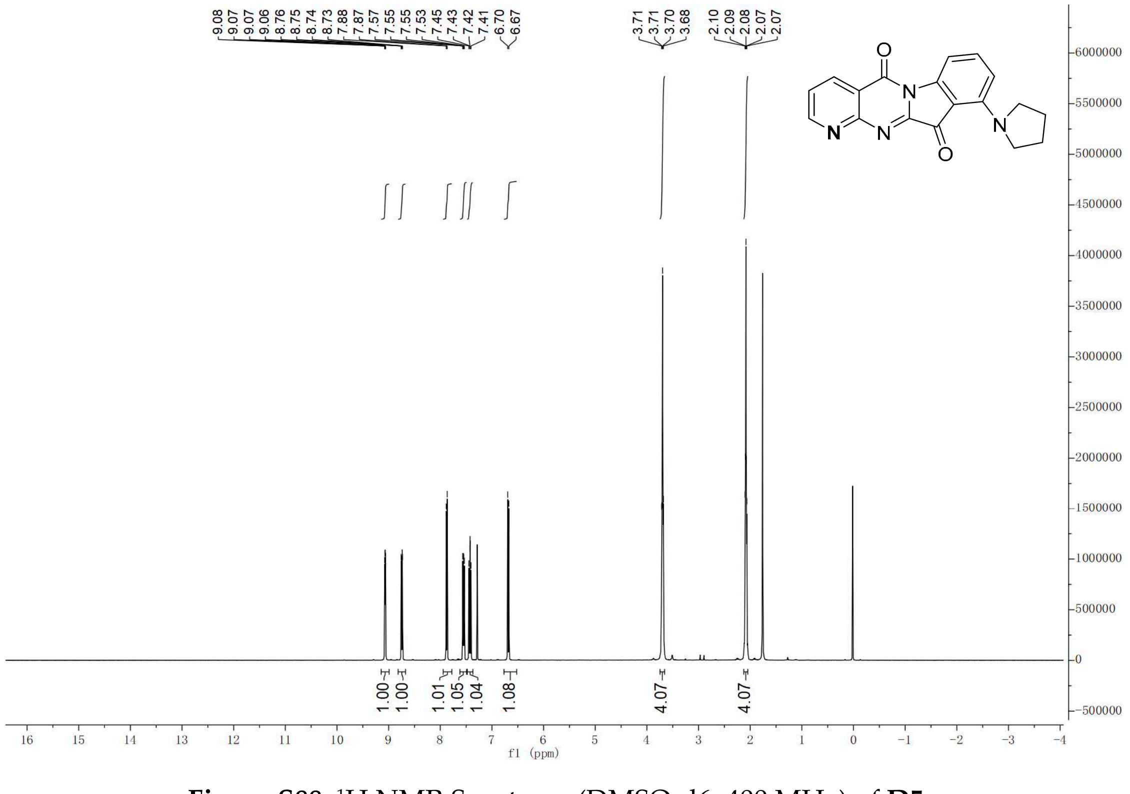

Figure S88. ^{1}H NMR Spectrum (DMSO-d6, 400 MHz) of **D5**.

Compound	IC50±SD[1] (μM)
	HEK[2]-293
C1	2.1±0.22

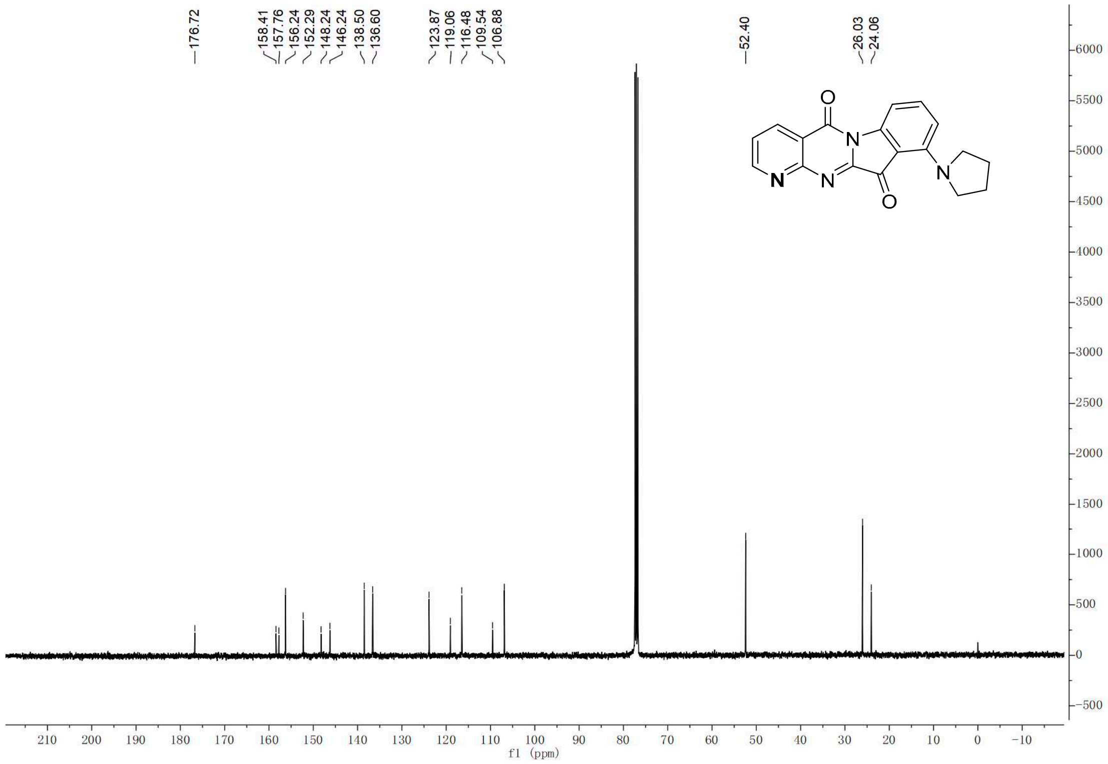

Figure S89. ^{13}C NMR Spectrum (Chloroform-*d*, 101 MHz) of **D5**.

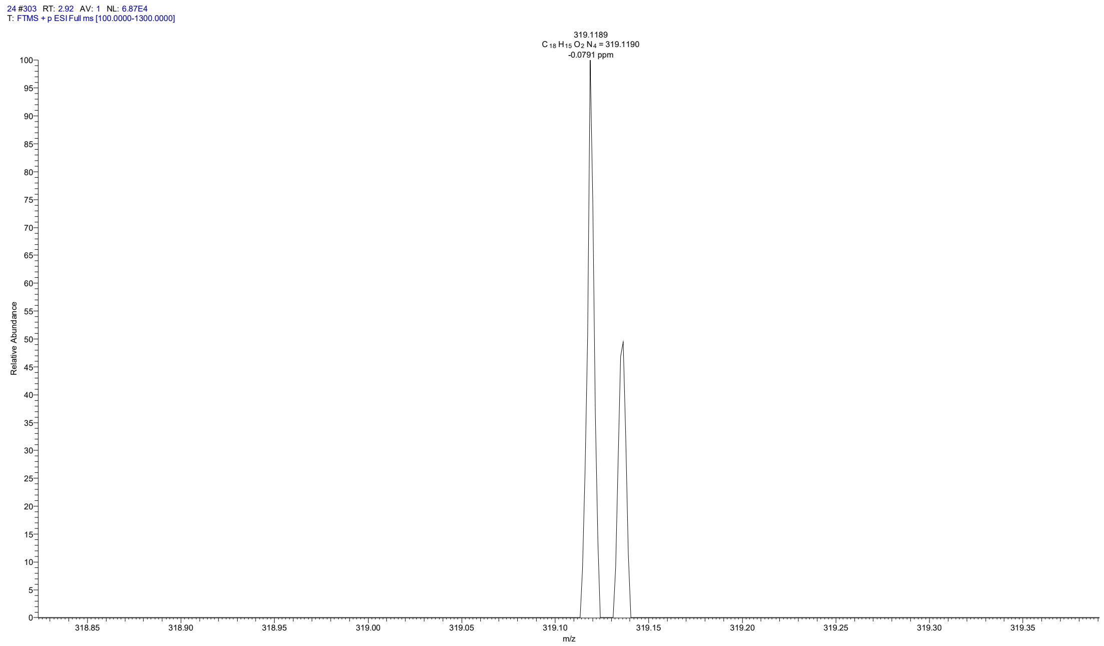

Figure S90. **D5** HRMS for $C_{18}H_{14}N_4O_2[M+H]^+$. Calcd. 319.1190. Found 319.1189.

7. Antiproliferative activity of compound C1 against normal cell lines.

Table S1. Antiproliferative activity of compound **C1** against normal cell lines.

Compound	IC_{50} ± SD[1] (μM)
	HEK[2]-293
**C1**	2.1 ± 0.22

[1] The inhibitory effect of the compound **C1** on the proliferation of the four cell lines was determined by the MTT assay. Results are expressed as the means ± SD. All experiments were independently performed at least three times.

[2] Human Embryonic Kidney cell.

8. Effects of C1 on the normal cell line HEK-293

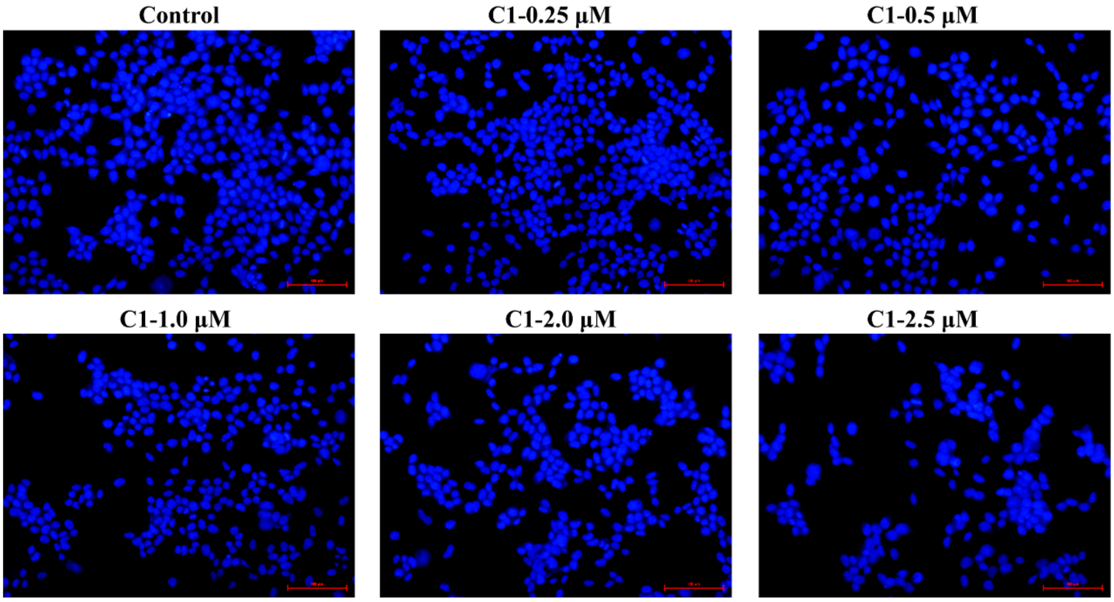

Figure S91. Effect of C1 on the normal cell line HEK-293. The cell morphology was observed under the fluorescence microscope (20x). Scale bars are 100 μm.

9. Effects of C1 on expressions of EGFR, p-EGFR, Akt, p-Akt

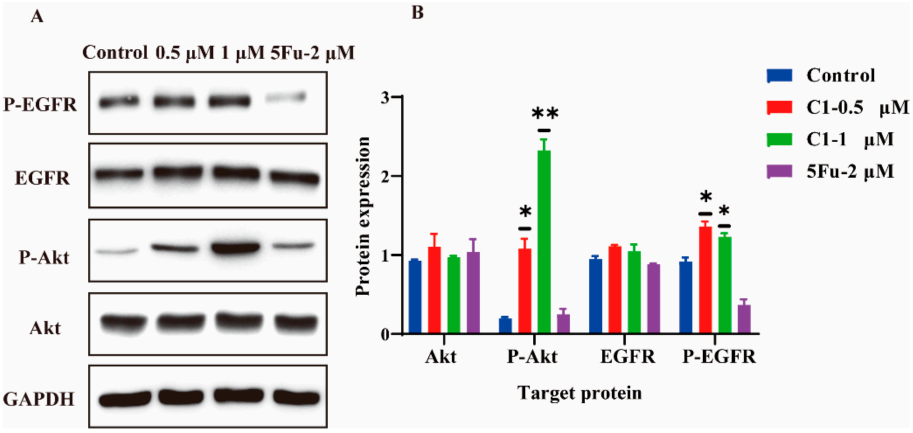

Figure S92. The effects of **C1** on the EGFR protein kinase signaling pathways. (A) A549 cells were incubated with different concentrations of **C1** for 48 h. The expression of EGFR, p-EGFR, Akt and p-Akt was detected by western blot. (B) Quantification of relative protein ex-pression levels of EGFR, p-EGFR, Akt and p-Akt. GAPDH was used as a loading control. The values are expressed as mean SD from three individual experiments. * Statistically significant (n = 3, $p < 0.05$). ** Statistically extremely significant (n = 3, $p < 0.01$).

10. ADME properties

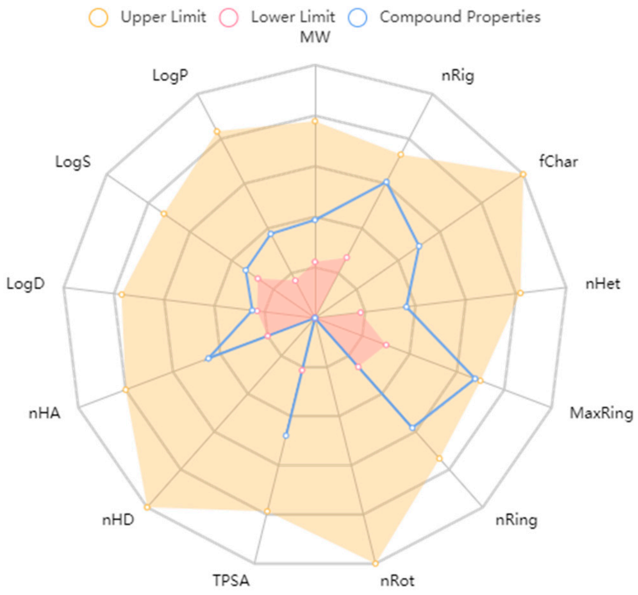

Figure S93. Predicted ADME properties of the target compound **C1** .

Table S2. Pharmacokinetic (ADME) profiles of compound **C1.**

**Compound**	**Absorption**			**Distribution**						
	**Log S**	**PCaco**	**%IA**	**P-gp substrate**	**Log VDss**	**%Fu**	**LogBB**	**BBB perm**	**Metabolism**	**Excretion tatal clearance log (ml/min/kg)**
**C1**	-4.72	-4.741	<30%	Yes	0.668	9.4	/	Yes	CYP1A2 inhibitor	0.484

Note: The pharmacokinetic profiles are calculated in silico using SwissADME and ADMETlab 2.0 online websites. Abbreviations: % IA, predicted human intestinal absorption percent; BBB perm., BLOOD-brain barrier permeability; Fu: fraction unbound in plasma; logBB, predicted blood – brain partition coefficient; LogS, predicted aqueous solubility coefficient of a compound (log mol/L); PCaco, predicted apparent Caco-2 cell perme ability (log Papp in 10^{-6} cm/s).

11. Reference

1. Yang, D.; Zhang, S.; Fang, X.; Guo, L.; Hu, N.; Guo, Z.; Li, X.; Yang, S.; He, J. C.; Kuang, C.; Yang, Q. N-Benzyl/Aryl Substituted Tryptanthrin as Dual Inhibitors of Indoleamine 2,3-Dioxygenase and Tryptophan 2,3-Dioxygenase. *J. Med. Chem.* **2019,** *62*, 9161-9174.
2. Wang, J.; Yun, D.; Yao, J.; Fu, W.; Huang, F.; Chen, L.; Wei, T.; Yu, C.; Xu, H.; Zhou, X.; Huang, Y.; Wu, J.; Qiu, P.; Li, W. Design, synthesis and QSAR study of novel isatin analogues inspired Michael acceptor as potential anticancer compounds. *Eur. J. Med. Chem.* **2018,** *144*, 493-503.
3. Klein, L. L.; Tufano, M. D. Synthesis of Substituted Isatins. *Tetrahedron Lett.* **2013,** *54*, 1008-1011.
